# Magnetic resonance imaging of the hand and wrist in a randomized, double-blind, multicenter, placebo-controlled trial of infliximab for rheumatoid arthritis: Comparison of dynamic contrast enhanced assessments with semi-quantitative scoring

**DOI:** 10.1371/journal.pone.0187397

**Published:** 2017-12-13

**Authors:** Chan Beals, Richard Baumgartner, Charles Peterfy, Andra Balanescu, Gavrila Mirea, Alexandru Harabagiu, Serghei Popa, Amy Cheng, Dai Feng, Edward Ashton, Julie DiCarlo, Marie-Helene Vallee, Bernard J. Dardzinski

**Affiliations:** 1 Department of Clinical Research, Merck & Co., Inc., Kenilworth, New Jersey, United States of America; 2 Spire Sciences, Inc., Boca Raton, Florida, United States of America; 3 Department of Immunology, U of Med and Pharm Carol Davila, Bucharest, Romania; 4 Department of Rheumatology, Tractorul County Hospital, Brasov, Romania; 5 German Diagnostic Center, Chisinau, MD, Moldova; 6 Department of Rheumatology, Republican Clinical Hospital, Chisinau, MD, Moldova; 7 VirtualScopics, Rochester, New York, United States of America; University of California, San Francisco, XX

## Abstract

The objective of this study was to compare the scope and the discriminative power of Dynamic Contrast Enhanced Magnetic Resonance Imaging (DCE-MRI) to those of semi-quantitative MRI scoring for evaluating treatments for rheumatoid arthritis (RA) in multicenter randomized clinical trials (RCTs). Sixty-one patients with active RA participated in a double-blind, parallel group, randomized, multicenter methodology study receiving infliximab or placebo through 14 weeks. The most symptomatic wrist and metacarpophalangeal joints (MCPs) were imaged using MRI. In addition to clinical assessments with DAS28(CRP), the severity of inflammation was measured as synovial leak of gadolinium based contrast agent (GBCA) using DCE-MRI (K_trans_, primary endpoint) at weeks 0, 2, 4, and 14. Two radiologists independently scored synovitis, osteitis and erosion using RA MRI Score (RAMRIS) and cartilage loss using a 9-point MRI scale (CARLOS). Infliximab showed greater decrease from baseline in DAS28(CRP), DCE-MRI K_trans_ of wrist and MCP synovium, and RAMRIS synovitis and osteitis at all visits compared with placebo (*p*<0.001). Treatment effect sizes of infliximab therapy were similar for DAS28(CRP) (1.08; 90% CI (0.63–1.53)) and MRI inflammation endpoints: wrist K_trans_ (1.00 (0.55–1.45)), RAMRIS synovitis (0.85 (0.38–1.28)) and RAMRIS osteitis (0.99 (0.52–1.43)). Damage measures of bone erosion (RAMRIS) and cartilage loss (CARLOS) were reduced with infliximab compared to with placebo at 14 weeks (*p*≤0.025). DCE-MRI and RAMRIS were equally sensitive and responsive to the anti-inflammatory effects of infliximab. RAMRIS and CARLOS showed suppression of erosion and cartilage loss, respectively, at 14 weeks. (ClinicalTrials.gov registration: NCT01313520)

## Introduction

Rheumatoid arthritis (RA) treatment options have expanded markedly over the past decade. However, as these therapies have become available, the acceptable duration for placebo control has also shortened, with rescue therapy typically offered within 14–16 weeks. The need to demonstrate both symptomatic and structure-modifying efficacy in randomized placebo controlled clinical trials (RCT) within this short time frame, along with growing difficulties recruiting RA patients into such studies, has made the use of radiography in RCTs impractical[1].

MRI has been shown to be more sensitive than radiography for detecting joint destruction in RA, and uniquely able to evaluate the up-stream inflammatory drivers of bone erosion and articular cartilage loss, namely osteitis and synovitis.

The most widely used method for monitoring bone erosion, osteitis and synovitis with MRI in RA clinical trials is RAMRIS (RA MRI Score), developed by OMERACT (Outcome Measures in Rheumatology) more than a decade ago[2]. The most widely used MRI method for evaluating cartilage loss in RA trials is the 9-point cartilage score (CARLOS) developed by Peterfy et al[3]. Dynamic contrast enhanced MRI (DCE-MRI) is a quantitative method for assessing synovitis based on the rate and magnitude of enhancement of synovial tissue by intravenously administered gadolinium-based contrast agents (GBCAs)[4–9]. However, DCE-MRI is more difficult to perform than is conventional contrast-enhanced MRI, upon which RAMRIS assessments are based, and unlike RAMRIS and CARLOS,[10] successful use of DCE-MRI based measures in multicenter RA trials has yet to be reported.

One of the challenges of DCE-MRI is achieving sufficient imaging speed to capture the change in T1 signal within inflamed synovium, while maintaining sufficient spatial resolution and anatomical coverage to delimit the entire synovial tissue from adjacent joint fluid accurately. The compromise of restricting enhancement measurements to small regions of interest (ROIs) within synovial tissue is confounded by the spatial heterogeneity of inflammation in RA. In this study, we overcame these challenges by devising a DCE-MRI technique suitable for multicenter clinical trials that imaged the entire wrist and MCP joints simultaneously and with sufficient speed to measure kinetic synovial enhancement parameters accurately. The baseline characteristics and inflammatory imaging endpoints from this study were previously used to derive a whole blood gene transcript signature that predicted a subsequent reduction in K_trans_ with infliximab[11]. In this report, we compare the short-term discriminative power and sensitivity to change of the volume transfer rate of GBCA from the blood plasma in synovium (K_trans_) with those of RAMRIS-synovitis in a randomized, controlled, multicenter trial of infliximab plus methotrexate (MTX) versus placebo plus MTX in patients with active RA. Both imaging methods similarly discriminated infliximab treatment from placebo on measures related to synovial inflammation, yet remained stable during placebo treatment. The RAMRIS and CARLOS methods had additional utility, identifying damage to bone and cartilage that could be prevented by infliximab. Appropriate MRI techniques, along with clinical measures of RA activity, should improve the characterization of drug effects on inflammation and structural damage in RA.

## Materials and methods

This was a 14-week, randomized, double-blind, placebo-controlled, methodology study (Study Protocol PO8136; S1 File) conducted from April 6, 2011 to March 29, 2012 in 3 clinical centers in Europe. The study was conducted in accordance with principles of Good Clinical Practice and was approved by the appropriate institutional review boards, the Comisia Nationala de Etica pentru Studiul Clinic al Medicamentului, Bucharest Romania and Comitetul National de Etica studiu clinic al medicamentelor si metodelor noi de tratament, Chisinau, Moldova. All subjects provided written informed consent. Key elements of the clinical protocol are available in supporting information (S1 File).

### Subjects

Male and female participants at least 18 years of age, with a diagnosis of RA for at least 6 months (based on the American College of Rheumatology [ACR] 1987 criteria), at least 6 tender and 6 swollen joints (using the 28 joint set), C-reactive protein (CRP) ≥1.0 mg/L or an Erythrocyte Sedimentation Rate ≥28 mm/hour, and who were on a stable dose of methotrexate, were recruited. All subjects were required to have RAMRIS synovitis score ≥ 1 in the radio-carpal or intercarpal joints based on centralized expert assessment of the baseline scan. The doses of all DMARDS, glucocorticoids (≤ 10 mg of prednisone or prednisolone) and NSAIDS were kept stable during the blinded portion of the study. In order to minimize risks associated with GBCA, subjects with estimated creatinine clearance of <60 mL/min or history of adverse reaction to GBCA were excluded. Patients had no contraindications to infliximab or MRI. Other requirements for enrollment included adequate hematological status, aspartate and alanine aminotransferase levels ≤2.5 times the upper limit of normal, and a clinically acceptable electrocardiogram. Patients taking oral corticosteroids were to be on a stable dose equivalent to ≤ 10 mg of prednisone (or prednisolone) per day for ≥ 2 weeks prior to the baseline visit.

### Treatment

At weeks 0, 2, 6, and 14, participants received 250 mL of either infliximab 3 mg/kg in 0.9% NaCl or identically appearing 0.9% NaCl infused over a 2-hour period, in 1:1 ratio, using computer generated randomization code prepared by the Sponsor using validated procedures and stratified by center with permuted block allocation. Study medication was prepared by an unblinded trained person using the randomization code and without other involvement in study procedures, assessments, or data recording. Treatment allocation was blinded to investigators, study staff, and subjects. Subjects continued to receive their standard dose and regimen of disease modifying antirheumatic drugs (DMARDS) (e.g. methotrexate and folate), nonsteroidal anti-inflammatory drugs (NSAID) or cyclo-oxygenase inhibitors (COXibs), and/or glucocorticoid. After 14 weeks of double-blinded therapy, participants in consultation with their physician could elect 3 months of open label infliximab treatment following the labeled dosing recommendations based on their previous randomized treatment assignment.

### Clinical assessments

DAS28(CRP) is a composite score of the number of tender joints (28 joint count), the number of swollen joints (28 joint count), patient global assessment of disease (GADP) on a 100 mm visual analog scale (VAS), and CRP (mg/dL)[12]. Joint counts were performed by examiners masked to treatment assignment who did not serve as study physicians.

### MRI assessments

MRI of the most clinically severe hand and wrist was acquired at baseline and weeks 2, 4, and 14 using a 1.5 Tesla whole body scanner (Siemens Avanto and GE Optima). The wrist, MCP and proximal interphalangeal (PIP) joints were included within a single field of view (FOV) using a commercial multi-channel knee coil[13]. An acrylic frame[10] was used to ensure fixed, reproducible positioning of the hand and wrist joints on serial MRI examinations. Two small tubes containing solutions of copper sulfate sufficient to provide T1 values of 90ms and 1080ms were made in a single batch and distributed to all clinical sites. Tubes were placed alongside the index and fifth fingers to serve as standards to verify appropriate T1 weighting, which is related to Gd concentrations for DCE-MRI measurements[14, 15].

The MRI protocol included coronal short-tau inversion recovery (STIR) and 3 dimensional T1-weighted gradient-echo with selective water excitation (3D GRE) scans. These were followed by a DCE-MRI sequence composed of 35 sequential 3D GRE (30 slices of 2.0-mm thickness, FOV of 18 cm x 13.5 cm, matrix of 192 x 144, selective water excitation; 9 sec/scan), with infusion of gadolinium diethylenetriaminepentacetate (Gd-DTPA) (Magnevist, Germany) (0.1 mM/kg, 0.2 cc/kg injected at 3 cc/sec, followed by a 20 cc saline flush) using a power injector after the sixth sequential scan. Following the DCE-MRI sequence the coronal 3D GRE scan was repeated and followed by an axial 3D GRE scan.

Small ROIs were placed manually over volumes of enhancing synovium or enhancing tissue (synovitis and osteitis) without knowledge of the treatment or the order in which examinations were acquired. Software identified enhancing tissue by comparison of pre- and post-GBCA images. A trained technologist removed enhancing tissue that represented blood vessels or skin, and the resulting enhancing tissue map underwent radiologist review. Care was taken to ensure that identical locations were selected for each study visit of an individual subject. The rate of GBCA synovial leakage was measured using a pharmacokinetic compartment model[16] that quantifies the exchange of contrast agent between the plasma and tissue extracellular space (synovium). The model requires a measure of the rate of GBCA input into the plasma, and is derived using an automated method[15,17] from T1 signal intensity in the radial artery over time. Outputs of the model include K_trans_ (sec^-1^), a rate constant that reflects the flow and permeability surface area of enhancing synovium (primary endpoint) and volume of enhancing tissue (synovitis and osteitis). The reproducibility of K_trans_ measurement ranged from 7% to 19% in tumors using identical analytical techniques[15]. One scan-rescan assessment of K_trans_ reproducibility in RA patients using this exact technique was consistent with that identified in tumors (Ashton, personal communication) and with published reliability using a similar approach[18].

RAMRIS^8^ of synovitis, osteitis and bone erosion at the wrist and MCP were determined at baseline, 2, 4 and 14 weeks by two independent radiologists blinded to visit order and treatment assignments. Cartilage loss was scored similarly but at only baseline and 14 weeks using the previously validated 9-point MRI scale (CARLOS), which also assesses the PIP joints not included in RAMRIS[9].

### Statistical methods

K_trans_ of wrist synovium was the primary endpoint of the study. Secondary endpoints included DAS28(CRP), K_trans_ of MCP synovium, and K_trans_ of enhancing tissue of the wrist plus MCPs. The DCE-MRI endpoints and the continuous clinical endpoints were compared between treatment groups using constrained Longitudinal Data Analysis (cLDA) proposed by Liang and Zeger[19]. The mean change from baseline to a given time point was estimated and tested from this model. A log transformation of the DCE-MRI parameters was performed to better meet the assumption of normality that is necessary for the cLDA. Analysis software included SAS v9, SAS Institute Inc., Cary, NC, USA and R (R Development Core Team, 2010).

Statistical significance of the dichotomous endpoints was determined using Fisher's exact test comparing placebo and treatment groups.

For RAMRIS and CARLOS, the average of the two readers’ scores were used (except in one case for which scores from only one reader were available), and statistical significance was determined using non-parametric van Elteren and Wilcoxon rank sum tests.

All analyses were conducted on the changes from baseline to Weeks 2, 4 and 14. Interpretation of the *p*-values for each endpoint was conducted in stepwise fashion (using a closed stepwise procedure) with *p*-values interpreted starting with Week 14. All *p*-values were reported as one-sided (alpha = 0.05), given that infliximab has only one direction of benefit based on these endpoints[20]. The treatment differences were estimated along with the associated 90% confidence intervals. Inter-reader agreement was assessed at baseline in terms of intra-class correlation coefficient (ICC). Smallest detectable change (SDC) thresholds for each RAMRIS feature and CARLOS were determined according to the method of Bruynesteyn et al[21].

The primary method of comparisons amongst endpoints was comparison of treatment effect size. For continuous endpoints, treatment effects of infliximab compared to those of placebo were expressed in terms of effect size (calculated from the cLDA model as difference between infliximab and placebo effects divided by the pooled standard deviation)[22]. For the RAMRIS endpoints and CARLOS, the effect sizes and 90% CI’s for the individual components were estimated using the U statistic obtained from Wilcoxon test and Newcombe's method. It was assumed that infliximab effect sizes for K_trans_ would be at least as large as those for DAS28, if the method was likely to be useful. A sample size of 26 patients per group has 80% power to yield a statistically significant (alpha = 0.05, 1-tailed, using a t-test) difference between treatments if the true underlying effect size for DAS28 is 0.7, seen in other studies of anti-TNF therapies[23, 24]. The study planned to enroll 30 patients per group to account for potential dropouts.

### Data availability

To protect the privacy and confidentiality of research participants, there are restrictions on the availability of data from this study (See Merck & Co., Inc. data sharing policy at: http://engagezone.merck.com/ds_documentation.php). Requests for access to the study data can be submitted through the EngageZone Web site or via email to dataaccess@merck.com.

## Results

**Fig 1:**
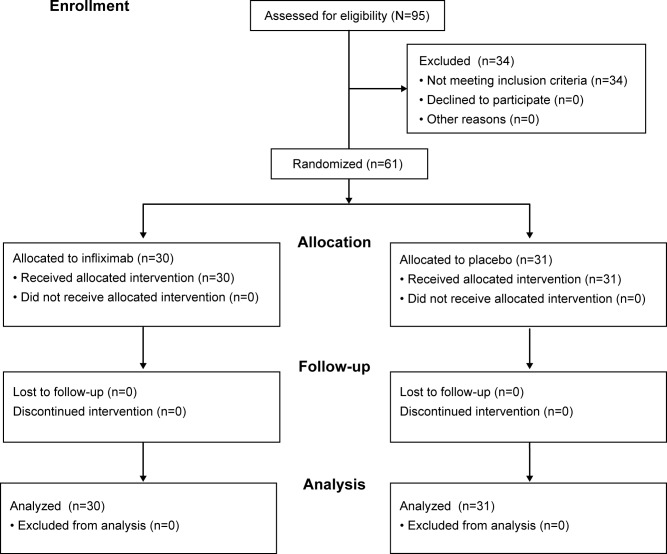
Study diagram.

Sixty-one adults with moderate to severe RA were enrolled into the study over a period of nine months (Fig 1). One patient meeting clinical criteria did not have sufficient synovitis at baseline on centralized expert reading to qualify, but was randomized to treatment in error. Since the patient was eligible on clinical grounds, the patient was not discontinued, and was included in all analyses. The cohort was 92% female with a mean age (standard deviation (SD)) of 50 (10) years. Ninety-one percent were positive for Rheumatoid Factor. Baseline characteristics are described in Table 1. The mean baseline DAS28(CRP) score was 6.2, indicating high disease activity. Baseline MRI scores also were consistent with high disease activity. There were no significant differences in any of these between the two arms, although higher RAMRIS synovitis scores in the infliximab group approached significance (*p* = 0.058). Clinical and MRI results are presented as the patients were randomized and treated. All patients completed the study.

**Table 1:** Baseline characteristics.

	Infliximab(n = 30)	Placebo(n = 31)	Total(n = 61)
Age. Years, mean (SD)	50 (10)	50 (11)	50 (10)
Gender, female, n (%)	28 (93)	27 (90)	56 (92)
DAS28(CRP), mean (SD)	6.1 (0.7)	6.2 (0.7)	6.2 (0.7)
Number of Tender Jointsmedian (IQR)	20.5 (15–23)	21 (17–25)	21 (17–23)
Number of Swollen Joints median (IQR)	12 (10–14)	12 (10–13)	12 (10–14)
CRP (mg/L), median (IQR)	9.2 (3.4–24.6)	9.1 (5.1–24.6)	9.3 (3.9–25.7)
Rheumatoid Factor Positiven (%)	26 (86.7)	28 (96.5)	54 (91.5)
Ktrans wrist enhancingsynovium (sec^-1^)median (IQR)	0.032 (0.025–0.039)	0.031 (0.025–0.042)	0.031 (0.025–0.040)
Ktrans MCP enhancingsynovium (sec^-1^)median (IQR)	0.030 (0.023–0.039)	0.031 (0.021–0.039)	0.030 (0.023–0.039)
Ktrans enhancing tissue (sec^-1^)median (IQR)	0.024 (0.021–0.032)	0.025 (0.020–0.031)	0.025 (0.021–0.031)
RAMRIS synovitismedian (IQR)	10.0 (7.5–14.5)	9 (4.0–11)	9.5 (6.5–13.0)
RAMRIS osteitismedian (IQR)	4.5 (2.5–15.0)	6.25 (1.5–19.5)	5.75 (1.5–18.0)
RAMRIS erosion median (IQR)	17.25 (11.0–24.0)	14.0 (8.5–21.5)	14.75 (9.5–22.0)
CARLOS, median (IQR)	7.0 (2.5–15.5)	15.0 (2.5–26.0)	9.5 (2.5–20.0)

IQR, Interquartile range

### Clinical outcomes

**Fig 2:**
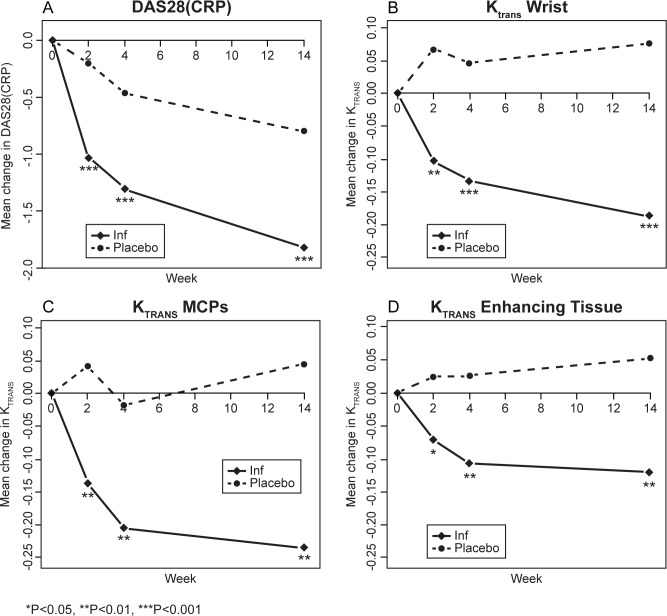
Mean changes from baseline (SE) in DAS28(CRP) and Dynamic Contrast Enhanced assessments of the wrist and metacarpophalengeal joint (MCP) in subjects with rheumatoid arthritis treated with infliximab 3 mg/kg (N = 30) or placebo (N = 31).

After only two weeks’ treatment, infliximab significantly reduced DAS28(CRP) disease activity compared with placebo (Fig 2A). At 14 weeks, infliximab-treated subjects had a least squares (LS) mean DAS28(CRP) (90% CI) that was 1.0 (0.62, 1.43) units lower than that of placebo treated patients. There were significant treatment effects of infliximab over placebo at 14 weeks in each of the components of DAS28(CRP) except VAS(GADP). As expected, placebo treatment also resulted in improvement in mean DAS28(CRP), but only approximately 40% that of infliximab treatment. Although CRP did not change with placebo treatment, swollen and tender joint counts and VAS(GADP) declined significantly by 14 weeks. ACR Responder Index also showed significant treatment differences. At 14 weeks, ACR 20 was 32.3% for placebo treated patients and 56.7% for infliximab treated patients (*p*<0.05 by Fisher’s exact test). At 14 weeks ACR 50 was 0% for placebo treated patients and 20% for infliximab treated patients (*p*<0.05 by Fisher’s exact test).

### MRI outcomes

Of 6325 bones examined for erosion and osteitis with RAMRIS, only 20 (0.32%) and 1 (0.02%) bones (all in the wrist), respectively, were not evaluable because of artifacts or other limitations in image-quality. Only 6 (0.20%) of 3,050 joints examined for cartilage loss were not evaluable because of artifacts or incomplete coverage; all of these were PIP joints at the distal edge of the field of view. All MCP and wrist joints were evaluable for assessing synovitis with RAMRIS and DCE-MRI. Inter-reader agreement was high: baseline ICCs for RAMRIS erosion, osteitis and synovitis and for CARLOS were 0.93, 0.92, 0.89 and 0.97, respectively. Sensitivity to change at the individual patient level was also high: SDCs were 1.52, 2.31, 2.24 and 1.25 units, respectively.

**Fig 3:**
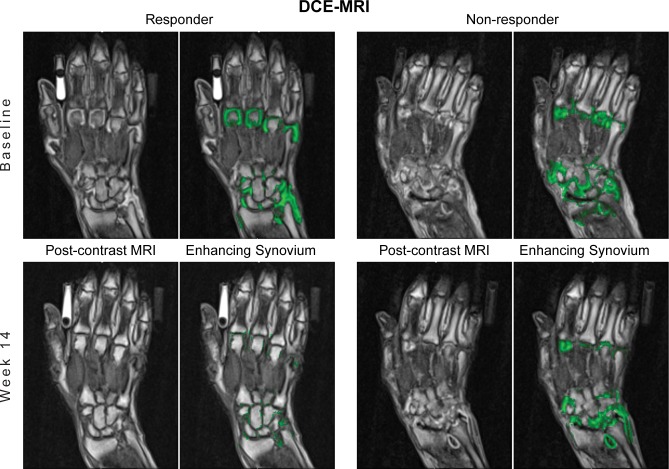
Baseline vs Week 14 DCE-MRI. Enhancing Synovium (green regions of interest) decreased dramatically from baseline to Week 14 in a clinical responder treated with infliximab (left), but was largely unchanged in a non-responder treated with placebo (right). Responder was defined as change from baseline in DAS28(CRP) >1.2 and non-responder as <0.6.

Representative DCE-MRI images are shown in Fig 3. Mean K_trans_ of synovium in the wrist and the MCPs each showed a significant treatment effect as early as 2 weeks following initiation of infliximab. This treatment effect was observed at each subsequent time point as well (Fig 2B and 2C). Placebo treatment resulted in no change in K_trans_ of wrist or MCP synovium. Mean K_trans_ of total enhancing tissue (synovitis and osteitis) in the wrist and MCPs similarly showed significant improvement at 2 weeks, 4 weeks and 14 weeks following treatment with infliximab but not placebo (Fig 2D). A secondary endpoint that is highly related to K_trans_ is IAUCBN90, the initial area under the gadolinium concentration-time curve for a tissue for the first 90 seconds after injection, which does not require the assumptions of a compartment model[15]. At the wrist, enhancing synovium measured by IAUCBN90 and K_trans_ were positively correlated at baseline (r = 0.98, p<0.001) and during each treatment (r > 0.98, p<0.001). Effects of infliximab on IAUCBN90 of wrist and MCPs were apparent by 2 weeks, and the effect size at 14 weeks for enhancing synovium at the wrist was (0.98; 90% CI (0.53, 1.42), comparable to that of DAS28.

To explore the utility of imaging to investigate individuals with low levels of RA disease activity, we performed exploratory analysis of DCE-MRI measures in subjects with DAS28(CRP) levels less than the median at baseline. In this subgroup of 31 individuals with DAS28(CRP) ≤ 6.2 at baseline, a significant difference between infliximab and placebo was seen at 14 weeks in both DAS28(CRP) (*p* = 0.010) and in K_trans_ of wrist synovium (*p* = 0.017) and in K_trans_ of MCP synovium (*p* = 0.02).

**Fig 4:**
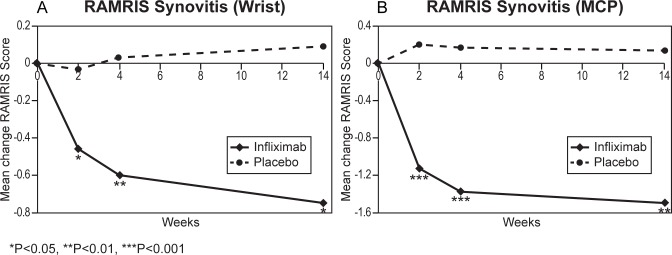
Mean changes from baseline (SE) in Rheumatoid Arthritis MRI Score (RAMRIS) of synovitis at the wrist and metacarpophalengeal joint (MCP) in subjects with rheumatoid arthritis treated with infliximab 3 mg/kg (N = 30) or placebo (N = 31).

**Fig 5:**
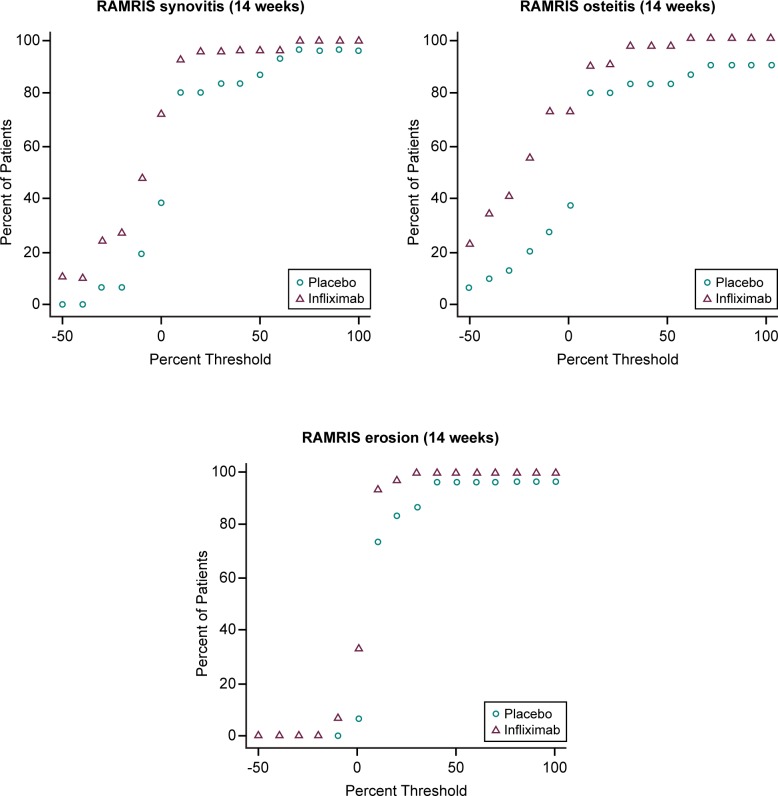
Cumulative probability of change from baseline in RAMRIS synovitis. The change in RAMRIS synovitis, osteitis and erosions between baseline and 14 weeks is shown by the percent of subjects less than the threshold of change for Placebo and Infliximab.

Infliximab significantly reduced the RAMRIS scores for both synovitis and osteitis in the wrist and MCPs as early as 2 weeks, and maintained reduction through 14 weeks (*p*<0.001, Fig 4 and Table 2). Both erosions and cartilage loss progressed in the placebo group, as expected for these measures of accumulating joint damage. The rates of progression were consistent with those reported from other RCT[25]. Change from baseline in RAMRIS erosion scores became significantly different between infliximab and placebo groups by 14 weeks. Cartilage loss was evaluated at baseline and 14 weeks only (due to project resource limitations); infliximab significantly reduced progression of cartilage loss at 14 weeks (Table 2, *p* = 0.025). Cumulative probability plots showed no outliers in any of the change data (Fig 5).

**Table 2:** MRI scoring of the wrist and MCP. Mean change from baseline (SD).

MRI score	Placebo (n = 31)			Infliximab (n = 30)		
	2W	4W	14W	2W	4W	14W
Synovitis	0.17 (0.81)	0.21 (1.10)	0.24 (1.98)	-1.6 (2.19)***	-1.98 (2.71)***	-2.30 (4.25)***
Osteitis	0.28 (0.98)	0.53 (1.49)	0.48 (3.22)	-1.2 (2.35)***	-1.43 (3.04)***	-3.10 (5.04)***
Total Inflammation	0.88 (2.95)	1.13 (3.86)	1.33 (7.51)	-5.9 (7.30)***	-7.38 (8.90)***	-10.0 (15.0)***
Erosion	0.10 (0.4)	0.27 (0.70)	0.85 (1.50)	0.08 (0.4)	0.18 (0.94)	-0.30 (1.70)**
Cartilage			0.27 (0.95)			-0.42 (1.96)*
Total Damage			1.51 (2.91)			-0.95 (4.92)*
Wrist						
Synovitis	-0.03 (0.4)	0.03 (0.4)	0.09 (1.08)	-0.46 (0.84)*	-0.6 (0.94)**	-0.75 (1.56)*
Osteitis	0.33 (0.93)	0.62 (1.35)	0.56(3.02)	-0.88 (2.21)***	-1.05 (2.72)***	-2.15 (4.04)***
Total Inflammation	0.23 (1.6)	0.67 (2.11)	0.91 (5.25)	-2.28 (3.60)***	-2.85 (4.12)***	-4.4 (7.09)***
Erosion	0.06 (0.31)	0.13 (0.47)	0.57 (1.02)	0.03 (0.26)	0.16 (0.88)	-0.13 (1.04)*
Cartilage			0.12 (0.63)			-0.14 (1.31)*
Total Damage			1.25 (2.56)			-0.68 (4.76)*
MCP						
Synovitis	0.2 (0.73)	0.17 (0.97)	0.14 (1.53)	-1.13 (1.60)***	-1.38 (1.99)***	-1.50 (2.9)**
Osteitis	-0.05 (0.66)	-0.08 (0.75)	-0.08 (0.81)	-0.28 (0.67)	-0.38 (0.92)	-0.93 (1.46)*
Total Inflammation	0.65 (2.44)	0.47 (3.07)	0.41 (4.37)	-3.68 (5.04)***	-4.55 (6.28)***	-5.53 (9.46)***
Erosion	0.03 (0.18)	0.13 (0.39)	0.28 (0.75)	0.05 (0.24)	0.01 (0.27)	-0.16 (0.82)*
Cartilage			0.02 (0.23)			-0.06 (0.48)
Total Damage			0.13 (0.73)			-0.13 (1.31)

Total Inflammation = Osteitis + 3 x Synovitis; Total Damage = Erosion + 2.5 x Cartilage

* *P*<0.05

** *P*<0.01

*** *P*<0.001

### Comparison among RA measures

At baseline, DAS28(CRP) correlated significantly with synovial K_trans_ in the wrist (Pearson correlation coefficient (90% CI) = 0.39 (0.19–0.55)) and MCPs (0.36 (0.16–0.53)) and with RAMRIS-synovitis in the wrist (0.29 (0.08–0.47)) and MCPs (0.54 (0.37–0.67)). Baseline DAS28(CRP) also correlated with baseline RAMRIS-osteitis in the MCPs (0.33 (0.13–0.51)), with baseline RAMRIS-erosion in the MCPs (0.43 (0.24–0.59)), and with baseline CARLOS (0.27 (0.04; 0.48)).

Change in DAS28(CRP) after 14 weeks of infliximab treatment correlated with change in synovial K_trans_ in the MCPs (0.33 (0.03; 058) but not the wrist and similarly with change in RAMRIS-synovitis in the MCPs (0.39 (0.10–0.62)) but not the wrist.

Synovial K_trans_ correlated with RAMRIS-synovitis scores at baseline in the wrist (0.53 (0.36–0.67)) and MCPs (0.61 (0.45–0.73)) and for change at 2 weeks, 4 weeks, and 14 weeks in the MCPs (0.34 (0.03–0.58), 0.39 (0.10–0.63) and 0.46 (0.18–0.67), respectively). Similarly synovial K_trans_ correlated with RAMRIS-osteitis scores at baseline in the wrist (0.40 (0.21–0.57)) and MCPs (0.37 (0.17–0.54)) and in the MCPs for change at 2 weeks and 4 weeks (0.48 (0.21–0.69), and 0.54 (0.27–0.72), respectively), though not at 14 weeks. Synovial K_trans_ also correlated with RAMRIS-erosion scores at baseline in the MCPs (0.26 (0.05–0.45)) but not for change, and with CARLOS at baseline in the MCPs (0.28 (0.05–0.48)) but not for change.

**Fig 6:**
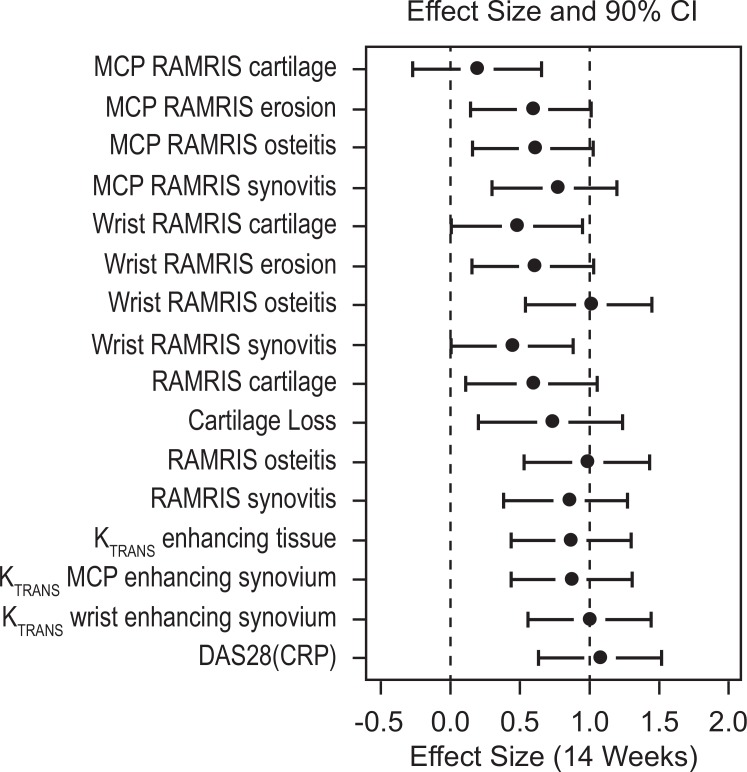
Effect sizes of treatment responses for various measures of rheumatoid arthritis for infliximab 3 mg/kg vs placebo. See statistical methods.

The effect size of DAS28(CRP) (90% CI) was 1.08 (0.63, 1.53), whereas those of K_trans_-wrist and K_trans_ -MCP were 1.00 (0.55–1.45) and 0.87 (0.43–1.31), respectively. The effect sizes of other MRI measures were close to 1 (Fig 6).

### Safety and tolerability

There were no serious adverse experiences or discontinuations for any reason. No subject required glucocorticoids to manage infusion reactions or for premedication to prevent infusion reactions. Subjects tolerated the MRI protocol well; all completed the study.

## Discussion

Clinical measures of RA used in clinical trials have several limitations. First, RA patients have a fluctuating disease course, and enrollment of patients at the apogee of disease activity may account for up to a third of the total DAS28 improvement in response to TNFα inhibitors[26]. Most studies report a substantial effect with placebo treatment on clinical endpoints[23,24], as was seen in the current investigation. DCE-MRI K_trans_ and RAMRIS synovitis and osteitis showed stable disease activity with placebo treatment, and each measure had a treatment effect size with infliximab that was quantitatively similar to the DAS28(CRP) benchmark. The robustness to placebo effects illustrates the utility of objective MRI measures for unambiguously identifying treatment effects in clinical trials using small sample sizes.

In current randomized studies, unresponsive RA patients are offered rescue treatment within 14–16 weeks, as recommended in regulatory guidance[1]. With short control treatment periods and a trend towards slowing structural progression rates[27], discriminating efficacy reliably with radiography would be challenging. We are not aware of any published RCT that demonstrates treatment efficacy with radiography in less than 24 weeks[25]. MRI has been shown in multiple RCTs to discriminate suppression of joint damage[3,28–30] and inflammation[28–35] in ≤12 weeks with relatively small numbers of patients per arm. In this study, RAMRIS and CARLOS demonstrated that infliximab suppressed bone erosion and cartilage loss within 14 weeks. To our knowledge, this is one of only two RCTs in RA to show that MRI can demonstrate slowing of cartilage loss in less than 24 weeks[32]. As pointed out, it is time for regulatory authorities to include MRI as an alternative method to demonstrate structural preservation with RA therapies[25].

A large body of evidence indicates that synovitis and osteitis are the underlying processes that drive bone erosions and cartilage loss in RA, which in turn lead to irreversible pain and physical impairment[36–38]. Quantitative MRI measures, such as K_trans_ of synovitis, and semi-quantitative MRI measures, such as RAMRIS of synovitis, osteitis and bone erosions, and CARLOS of cartilage loss, have been shown to be sensitive and reproducible measures of the inflammation and structural damage that occur in RA[3,10,25,35,37,39]. RAMRIS and CARLOS measures have been used successfully in several multi-center RCTs to demonstrate treatment efficacy[3,29,30,32–35,39,40]. However, successful use of DCE-MRI in a multi-center RCT has not yet been reported. One reason for this may be the technical challenges associated with performing DCE-MRI reproducibly across multiple time points and multiple clinical sites. We were able to perform this technique successfully in a multicenter clinical trial using a knee coil to image both the wrist and MCPs simultaneously[13] Synovial K_trans_, all RAMRIS scores and CARLOS correlated with DAS28(CRP) at baseline and correlations for change were significant in the MCPs. The correlations demonstrated in this study support the validity of these MRI endpoints as measures of clinical outcomes in RA. Within the limitations of our sample size, this study suggests that RAMRIS and K_trans_ have similar abilities to discriminate anti-inflammatory treatments. Since RAMRIS and CARLOS have broader scope than DCE-MRI and are easier to implement in multicenter clinical trials, we find no advantage to recommend the use of DCE-MRI as implemented in this study.

DCE-MRI can be interpreted in a model-free fashion, by measuring the early-enhancement rate and maximal enhancement of the enhancement curves. These empirical measurements are reliable and have been correlated with cellular infiltration and vessel density in the rheumatoid synovium[4–9], and are responsive to treatment[41–43]. While somewhat easier to measure, these measurements depends on pulse sequence and machine parameters, rendering comparisons among centers difficult. Because of our interest in methodologies to support clinical testing of RA therapies, we used a compartment model to interpret DCE-MRI data because the model parameters are independent of the imaging and injection conditions and should be more robust in multicenter trials. The DCE-MRI quantitation techniques used in this study have been validated in several multicenter clinical trials in other indications[15,44,45]. The simple compartment model has one vascular compartment and one tissue compartment and the measured signal is used to derive the constant of proportionality in the leak of GBCA from capillaries to tissue, K_trans_[16]. K_trans_ has been shown to discriminate among patient groups with early arthritis and change with treatment[9,18].

A potential limitation of this study is that K_trans_ measurements were not repeated by a second delineation of synovium. However, two individuals blinded to treatment and acquisition order were involved in setting synovial boundaries, and the method used here was previously validated for rheumatoid joints. Furthermore, statistically significant differences in K_trans_ between placebo and infliximab at weeks 2, 4 and 12 establish the responsiveness of K_trans_ when implemented in blinded fashion. The discrimination of K_trans_ endpoints is not likely to be impacted by the 0.9 mm resolution of the images in this study, given that synovium is a large structure and signal within the ROI is averaged across pixels. However, the method might be made more discriminative ROI were placed with greater reproducibility than by blinded technologists using landmarks.

## Conclusion

Both DCE-MRI and RAMRIS measures of RA inflammation are as sensitive to treatment effects with infliximab as is the standard clinical measure of RA activity, DAS28(CRP), identifying effects as soon as 2 weeks in small numbers of patients. Furthermore, MRI measures of joint damage (RAMRIS-erosion and CARLOS) can discriminate treatment effects as soon as 14 weeks. In contrast to clinical measures, however, MRI measures are not vulnerable to placebo effects. Appropriate MRI techniques, along with clinical measures of RA activity, should improve the discrimination of drug effects in reducing inflammation and structural damage in RA.

## Supporting information

S1 File Redacted study protocol. (PDF)

S2 File CONSORT checklist for clinical trials. Required checklist for published clinical trials.
(DOC)
