# Supplementary material for: Magnetic resonance imaging of the hand and wrist in a randomized, double-blind, multicenter, placebo-controlled trial of infliximab for rheumatoid arthritis: Comparison of dynamic contrast enhanced assessments with semi-quantitative scoring
Source: PLoS One. 2017 Dec 13;12(12):e0187397. doi: 10.1371/journal.pone.0187397 (PMC5728526; doi:10.1371/journal.pone.0187397)
Supplement: S1 File — (PDF) [file pone.0187397.s001.pdf]

Merck & Co., Inc. policy on posting of redacted study protocols on journal websites is described in Guidelines for Publication of Clinical Trials in the Scientific Literature on the www.merck.com website.

For publicly posted protocols, the Company redacts content that contains proprietary and private information.

This report may include approved and non-approved uses, formulations, or treatment regimens. The results reported may not reflect the overall profile of a product. Before prescribing any product mentioned in this report, healthcare professionals should consult local prescribing information for the product approved in their country.

Copyright © 2017 Merck Sharp & Dohme Corp., a subsidiary of Merck & Co., Inc.

All Rights Reserved. Not for regulatory or commercial use.

1 TITLE PAGE

05 JAN 2011 - INITIAL PROTOCOL

C O N F I D E N T I A L
STUDY PROTOCOL

Abbreviated Title	RA DCE-MRI Study
Title	A randomized clinical trial to study the effects of infliximab on clinical efficacy and hand and wrist magnetic resonance imaging (MRI) in patients with active rheumatoid arthritis (RA)(Protocol No. P08136)
Sponsor	Schering-Plough Research Institute, a Division of Schering Corporation
Sponsor's Address	2015 Galloping Hill Road<br>Kenilworth, NJ 07033, USA
IND No.	Not Applicable
EudraCT No.	2011-000079-14
SPRI Trial Physician/Director	PPD
Doc ID	5465145
Study type	Experimental Medicine; Phase 2
Current NME Development Phase	4
Date of Finalization of This Current Version of the Protocol	05 JAN 2011 Initial Protocol
Previous Version(s) of the Protocol	N/A
Protocol Template Version	3.2

CONFIDENTIAL

TRIAL PROTOCOL

THIS CONFIDENTIAL INFORMATION ABOUT AN INVESTIGATIONAL DRUG OR PRODUCT IS PROVIDED FOR THE EXCLUSIVE USE OF INVESTIGATORS OF THIS DRUG OR PRODUCT AND IS SUBJECT TO RECALL AT ANY TIME. THE INFORMATION IN THIS DOCUMENT MAY NOT BE DISCLOSED UNLESS SUCH DISCLOSURE IS REQUIRED BY APPLICABLE LAW OR REGULATIONS. SUBJECT TO THE FOREGOING, THIS INFORMATION MAY BE DISCLOSED ONLY TO THOSE PERSONS INVOLVED IN THE TRIAL WHO HAVE A NEED TO KNOW, WITH THE OBLIGATION NOT TO FURTHER DISSEMINATE THIS INFORMATION. THESE RESTRICTIONS ON DISCLOSURE WILL APPLY EQUALLY TO ALL FUTURE ORAL OR WRITTEN INFORMATION SUPPLIED TO YOU BY THE SPONSOR OR ITS AFFILIATES OR REPRESENTATIVES THAT IS DESIGNATED AS "PRIVILEGED" OR "CONFIDENTIAL".

2 SYNOPSIS

**Title of Study:** A randomized clinical trial to study the effects of infliximab on clinical efficacy and hand and wrist magnetic resonance imaging (MRI) in patients with active rheumatoid arthritis (RA) (Protocol No. P08136)		
**Abbreviated Title:** RA DCE-MRI Study		
**Study Centers:** 3 to 6		
**Duration of Study:** Approximately 12 months	**Protocol Type:** Experimental Medicine (Methodology) Phase 2	**Clinical Project Phase:** Experimental Medicine
**Study Classification:** ☐ **NA: Not applicable** ☐ **Safety** ☐ **Efficacy** ☐ **Safety/Efficacy** ☐ **Bio-equivalence** ☐ **Bio-availability** ☐ **Pharmacokinetics** X **Pharmacodynamics** ☐ **Pharmacokinetics/dynamics**		

Objectives

Primary Objective:

To compare the effect of infliximab on synovial inflammation as measured with Dynamic Contrast Enhanced (DCE)-MRI of one wrist with placebo over 14 weeks of treatment. The primary hypothesis is that the change in K^{trans} in total enhancing synovium with infliximab is larger than placebo.

Secondary Objectives:

- To compare the effect of infliximab with placebo on various MRI measures of disease activity including:
  - Other DCE-MRI endpoints including K^{trans} of total enhancing tissue of wrist; $IAUC_{90,}$ Early Enhancement Rate (EER) and maximal enhancement (ME) of total enhancing tissue and total enhancing synovium of wrist, and enhancing synovial volume of wrist and/or metacarpophalangeal (MCP)
  - RA MRI Scoring (RAMRIS) of osteitis and synovitis of wrist and MCP.
- To compare the effect of infliximab with placebo over 14 weeks of treatment on the following clinical measures of disease activity:
  - Clinical disease activity (DAS28 (CRP))
  - American College of Rheumatology criteria for 20% improvement (ACR20) and for 50% improvement (ACR50).
  - American College of Rheumatology criteria for x % of improvement over the range of x=5 to 45% (ACRx).

  (The purpose of this objective is to assess for the possibility of a failed clinical study and to benchmark imaging biomarkers against standard clinical biomarkers.)
- To compare infliximab against placebo over 14 weeks with two composite measures of RA disease activity that incorporate DAS28(CRP) with K^{trans} or RAMRIS.
- To correlate changes in clinical disease activity (DAS28(CRP)) and changes in MRI biomarkers of disease activity:
  - RAMRIS synovitis and osteitis
  - DCE-MRI parameters

Exploratory Objectives:

- To assess the sensitivity and specificity of blood mRNA signatures and DNA polymorphisms that predict clinical response to infliximab.
- To evaluate the responsiveness of endpoints available in Dynamika RA software to infliximab compared with placebo
- To compare the effect of infliximab with placebo on synovial inflammation as measured by K^{trans} derived from DCE-MRI of all segmentable synovium in wrist and all MCP in the field of view (FOV).
- To compare the effect of infliximab with placebo on RAMRIS scoring of bone erosion in wrist and MCP.
- To compare the effects of infliximab with placebo on a measure of inflammation that sums RAMRIS osteitis and synovitis scores, and a measure of damage that sums RAMRIS bone erosion and non-RAMRIS cartilage damage.
- To compare the effect of infliximab with placebo on the volume of osteitis

**Title of Study:**	A randomized clinical trial to study the effects of infliximab on clinical efficacy and hand and wrist magnetic resonance imaging (MRI) in patients with active rheumatoid arthritis (RA) (Protocol No. P08136)

Rationale

a) Therapeutic Rationale:

Infliximab is used in this placebo controlled methodology study because it is an established therapy for active RA with nearly full therapeutic impact within 3 to 4 months treatment. It is the first registered anti-tumor necrosis factor (TNF) therapy and its safety and efficacy is well understood. Infliximab is administered by IV infusion in the outpatient clinic, which facilitates compliance and masking of the treatment.

b) Study Conduct Rationale:

Composite disease activity instruments (e.g. DAS28) have improved the ability to evaluate the course of RA, but do not measure the underlying pathological process – synovitis of the RA joint. We hypothesize that MRI measures of synovial and bone marrow inflammation (osteitis), alone or in combination with DAS28, will improve the discrimination of effective drugs in active established RA, allowing objective, faster and superior development decisions of new drugs for RA. This study benchmarks two MRI disease activity measure in the wrist and hand to clinical activity change, quantified using DAS28. One MRI measure is called dynamic contrast enhanced (DCE)-MRI, and quantifies parameters that characterize vascularization of synovium during a controlled contrast injection. The second MRI measure is a validated ordinal scoring system of hand synovitis and osteitis called the Rheumatoid Arthritis MRI Score (RAMRIS) system. This study will also evaluate the feasibility of using DCE-MRI at multiple centers capable of enrolling rheumatoid arthritis patients to drive program decisions, since there are no publications of DCE-MRI in multicenter trials in RA.

c) Study Design Rationale:

RA patients have a fluctuating disease course and most trials demonstrate a strong placebo effect. Clinical disease activity at enrollment is required for any of the imaging methods described herein to show a treatment effect, and this is also the case for improvement in clinical disease activity. Consequently, randomized, placebo controlled, physician and patient-masked studies are the only study design from which the adequacy of biomarkers can be fully assessed. RA patients with established, active disease despite methotrexate (MTX) treatment, and with evidence of MRI wrist synovitis will participate. RAMRIS and DCE-MRI have sufficient criterion validation (correlation with gold-standard disease activity metrics, histological correlation, or alignment with other valid imaging modalities), and can be measured with precision, and are incorporated at multiple time points to fully characterize the kinetics of imaging biomarker responses. It is highly desirable to compare the responsiveness of DCE-MRI biomarkers to DAS28, so we select the sample size based on an estimated treatment effect size of 0.7 to 0.8 on DAS28.

d) Dose Rationale:

In this study, infliximab is used at the dose of 3 mg/kg on the labeled schedule of 0, 2, 6 weeks and every 8 weeks thereafter. The clinical efficacy of this dose and schedule of infliximab has been amply demonstrated ([1]). The primary time point for the study is after the 4th treatment at 14 weeks, a time at which most patients can be evaluated for response to anti-TNF therapy. The Sponsor of the study will make open-label infliximab available for optional use for patients completing the study for a maximal duration of 16 weeks.

Overview:

This study is a randomized, placebo-controlled, parallel-group, multi-site, double-blind study of infliximab (SCH 215596) in patients with rheumatoid arthritis to be conducted in conformance with Good Clinical Practices.

Title of Study: A randomized clinical trial to study the effects of infliximab on clinical efficacy and hand and wrist magnetic resonance imaging (MRI) in patients with active rheumatoid arthritis (RA) (Protocol No. P08136)

Study Plan:

Patients with active RA on a stable dose of methotrexate (MTX) meeting study eligibility criteria will be randomized to receive either infliximab 3 mg/kg or placebo for 14 weeks, with doses administered intravenously over a 2-hour period at Weeks 0, 2, 6, and 14. A baseline MRI with gadolinium based contrast agents (GBCA; e.g. Gd-DTPA) taken of one wrist and MCP joints (1-5) will be obtained and must show evidence of wrist synovitis before the patient undergoes treatment with either infliximab or placebo at Week 0. Patients will be assessed for RA activity using DAS28 by a masked joint examiner who has no other involvement with the study conduct and for adverse events before each infusion of study drug or MRI. MRI with GBCA will be obtained at Week 2, 4 and 14 in the same wrist. The 2 week MRI may be removed from the protocol procedures for all patients based on Sponsor assessment of feasibility. The study will conclude after the 14 week procedures. Adverse experiences will be collected throughout the study, and for an additional 2 weeks afterwards. Patients who receive glucocorticoids to manage infusion reactions or for premedication to prevent infusion reactions will be discontinued from this study. At the completion of week 14 procedures, after receipt of a technically adequate MRI scan and successful resolution of safety data for each patient, the treatment assignment will be unblinded to the Investigator. As a courtesy to the patients who completed the study, open-label treatment with infliximab after completion of the main study will be provided in consultation with a treating rheumatologist for up to 16 weeks and in accordance with the approved dosing instructions outlined in the SmPC. Patients previously assigned to placebo may receive a standard induction regimen at 0, 2, 6 and 14 weeks, while patients previously assigned to infliximab may receive two additional treatments every 8 weeks. The cost of these open-label supplies will be covered by the Sponsor.

Duration of Treatment:

Randomized Treatment Blinded Period: 14 weeks

Open Labeled Treatment Period with Infliximab: Up to 16 weeks

Subject Key Inclusion/Exclusion Criteria:

Male and female patients (≥18 years of age) with a diagnosis of moderate-to-severe RA with, at least 6 tender and 6 swollen joints (28 joint set), and a CRP mg/L ≥ 1.0 OR ESR ≥28 mm / hr will be selected for the study. The wrist with the greatest clinical involvement at baseline will be imaged by MRI through the entire study. Synovitis on MRI with RAMRIS score ≥ 1 in radio-carpal or intercarpal joint regions must reported by central reader for the patient to be randomized. Technical adequacy of the MRI will be monitored and patients may be asked to repeat MRI examinations. Specific disease activity criteria and MRI criteria are flexibly defined and may be altered for all patients by the Sponsor based on feasibility. Patients must have a diagnosis of RA for ≥6 months, on stable doses of methotrexate for 8 weeks, with no prior treatment with anti-TNF that were discontinued for lack of efficacy or for safety. Patients will have no contraindication to infliximab, MRI or GBCA.

Type of Blinding: Patient and physician are masked to treatment. An unblinded trained person will prepare infliximab or saline placebo for IV administration using equipment to obscure the IV bag and tubing. The unblinded trained person preparing study drug will have no other involvement in patient management and will not administer study treatment. A masked joint examiner will record the number of tender and swollen joints using the 28 joint set for the calculation of DAS28(CRP), but will have no other involvement in patient management.

Sample Size (Including Ratio of Subjects Assigned to Treatments)/Power: It is assumed that infliximab effect sizes for K^{trans} will be at least as large as those for DAS28. The sample size of 26 patients per group has 80% power to yield a statistically significant (alpha=0.05, 1-tailed) difference between treatments if the true underlying effect size is 0.7. The study plans to enroll 30 patients per group to account for potential dropouts.

Subject Replacement Strategy: Subjects who discontinue may be replaced at the discretion of the sponsor in consultation with the Investigator.

Randomization: Patients will be randomized in a 1:1 ratio to receive either infliximab or placebo.

Stratification: By center

Test Product, Dose, Mode of Administration: Infliximab (SCH 215596) 3 mg/kg (IV) in 250 ml of 0.9% sodium chloride, infused over a 2-hour period. Additional doses at 2 and 6 and 14 weeks after the first infusion.

Reference Therapy, Dose, Mode of Administration: 250 ml of 0.9% sodium chloride used as placebo is administered IV over a 2-hour period, following the same schedule as described for infliximab.

**Title of Study:**	A randomized clinical trial to study the effects of infliximab on clinical efficacy and hand and wrist magnetic resonance imaging (MRI) in patients with active rheumatoid arthritis (RA) (Protocol No. P08136)

Criteria for Evaluation:

Safety: Adverse events, vital sign measurements (heart rate, blood pressure, respiratory rate, temperature), clinical laboratory tests (hematology with complete blood count and chemistry.)

PD:

Disease Activity, DAS28(CRP): components are the number of tender joints (28 joint count), the number of swollen joints (28 joint count), a Patient Global Assessment of Diease Status (GADP) on a 100 mm visual analog scale (VAS), and CRP (mg/L). The formula for determining DAS28(CRP) is as follows:

$$DAS28(CRP) = 0.56*\sqrt{(TJC28)} + 0.28*\sqrt{(SJC28)} + 0.36*\ln(CRP+1) + 0.014*GADP\ (VAS) + 0.96,$$

with the following 28 joints: Finger Proximal Interphalangeal Joints (8), thumb interphalangeal joint (2), metacarpophalangeal (MCP) (10), wrists (2) (includes carpometacarpal, intercarpal, and radiocarpal), elbows (2), shoulders (2), and knees (2). Joint counts by independent masked examiner.

American College of Rheumatology responder criteria (ACR20): requires that both tender and swollen joint counts improve by at least 20% from baseline during the course of a trial, as well as 20% improvement of at least 3 of the other ACR/European League Against Rheumatism (EULAR) core set measures (pain, patient's and physician's global assessment, physical disability, and CRP). Similar definitions are used for ACR50 and discrete and continuous definitions of ACRx.

Scoring of Synovium, Osteitis, Bone Erosion, and Cartilage Damage:

- RAMRIS Synovitis is scored 0-3 in 8 locations (0-24 total)
- RAMRIS Osteitis is scored 0-3 in 25 locations (0-75 total)
- RAMRIS Erosion is scored 0-10 in 25 locations based on the percentage of volume of bone eroded 1= 1-10%, 2=11-20%, etc); Total scores for erosion (0-250)
- Cartilage is scored 0-4 in 25 locations based on the extent of cartilage destruction (0=normal, 0.5=equivocal thinning, 1.0=mild but definitive thinning, 1.5= <25% thinning, 2.0= 25%-75% thinning, 2.5= >75% loss, 3.0= complete cartilage loss, 3.5= partial ankylosis, 4.0= complete ankylosis) with total score for cartilage (0-100).

DCE-MRI:

- K^{trans} of the wrist. K^{trans} reflects flow and permeability surface area of enhancing tissue and enhancing synovium.
- $IAUC_{90}$ reflects flow and permeability surface area and the contrast agent distribution volume in the first 90 seconds after injection, normalized on an individual patient basis.
- The volume of synovial enhancing tissue
- Early Enhancement Rate (EER) (%/sec) and Maximal Enhancement (ME).
- The number of voxels showing contrast uptake, contrast plateau, and contrast washout, alone and in combination.

PK: Not applicable

PG: The effect of the following genetic polymorphisms on clinical response to infliximab will be investigated (see **Section 6.3**):

- v-maf musculoaponeurotic fibrosarcoma oncogene homolog B (MAFB) on chromosome 20 (rs6028945 & rs6071980)
- type I interferon gene (IFNk) on chromosome 9; (rs7046653)
- paraoxonase I (PON1) gene on chromosome 7 (rs854555, rs854548, rs854547)
- TNFα gene promoter, known as the 308 G/A variant (TNF-308) (rs1800629)

The effect of the following gene transcript signatures at baseline on clinical response to infliximab will be investigated:

- Julia 8-gene Signature
- Bienkowska 8-gene Signature
- Sekiguchi 18-gene Signature
- Tanino 300-gene Signature
- Lequerré 8-gene Signature
- Koczan 8-gene Signature
- IFN 5-gene Signature

Title of Study: A randomized clinical trial to study the effects of infliximab on clinical efficacy and hand and wrist magnetic resonance imaging (MRI) in patients with active rheumatoid arthritis (RA) (Protocol No. P08136)

Statistical Methods:

Safety: Adverse events, vital sign measurements, and safety laboratory parameters will be listed for each patient and tabulated by treatment and summarized using descriptive statistics

PD: The primary metric for assessing treatment effect at Weeks 2, 4 and 14 is change from baseline. The baseline MRI scan of the first 6 to 10 patients randomized to the protocol will be investigated to determine the likelihood that the primary DCE-MRI parameter, K^{trans}, can be measured with a patient specific Arterial Input Function (AIF) in the majority of patients. If these conditions are not met, then a population AIF will be used to compute K^{trans}. If satisfactory data cannot be collected, then the order of MRI endpoints ($IAUC_{90}$, EER, and RAMRIS) will potentially be reordered to accommodate either data with technically satisfactory collection or to accommodate more tolerable MRI acquisitions. The DCE-MRI parameter, K^{trans}, will be compared among the treatment groups via constrained Longitudinal Data Analysis (cLDA) according to Liang and Zeger. This model assumes a common mean across treatment groups at baseline and different mean for each treatment at each of the post-baseline time points. In this model, the response vector consists of baseline and the values observed at each post-baseline time point. The time is treated as a categorical variable so that no restriction is imposed on the trajectory of the means over time. The treatment mean in terms of the mean change from baseline to a given time point will be estimated and tested from this model.

Normality and homogeneity of error variance assumptions will be assessed and appropriate data transformation applied if departures from these assumptions are observed.

Statistical significance of the dichotomous endpoints (ACRx) will be determined using chi-square test comparing placebo and treatment groups. Similarly to other continuous endpoints, the continuous form of ACRx will be analyzed by cLDA. We will use cLDA as with other continuous endpoints.

P-values and 90% CI's will be computed. These analyses will be carried out for change from baseline at each of weeks 2, 4 and 14. Interpretation of p-value testing for each endpoint will be made in step-down fashion (using closed stepwise procedure) with week 14 interpreted first. Only if significant treatment difference is observed for week 14, then treatment difference testing of week 4 and then week 2 will be examined. Significance test will be carried out at alpha=0.05, 1-sided for the primary and other study endpoints.

Effect sizes and associated 90% CI's will be computed to assess precision of each endpoint.

Composite Endpoints: Analysis of secondary objectives will be carried out aimed at determining whether a composite of K^{trans} and DAS28 endpoints or RAMRIS inflammation scores and DAS28 has improved precision for discrimination between active treatment from placebo. Precision of the composite will be measured as effect size (infliximab minus placebo divided by pooled SD). Prior to analysis for composites, the individual endpoints (components of the composite) will be standardized using z-scores. The methods used for building composites include O'Brien's global statistics and the first principal component computed from individual endpoints after z-score standardization. For example, O'Brien's global statistics is the average of standardized z-scores across the measures. Similar calculation will be done for a composite of RAMRIS synovitis, osteitis and DAS28.

PK: Not applicable

PG: Classification model for prediction of responders and non-responders from gene signatures and single nucleotide polymorphisms (SNPs) will be built using multivariate classifiers (logistic regression, random forest). The model will be validated using external cross-validation to account for over-optimism of the (internal) model building. In addition, the SNPs will be tested for their odds ratio of risk allele in responder vs. non responder groups. Prior to dichotomization of the clinical scores, regression model using random forest will be developed to predict DAS28(CRP) scores on the continuous scale. Again, external (nested) cross validation will be used to validate the model.

Interim analysis: No formal interim analysis will be performed.

Day and Visit Structure: See Section 2.2, Study Flow Chart for further details.

Key Words: arthritis, rheumatoid; magnetic resonance imaging

2.1 Study Design Diagrams

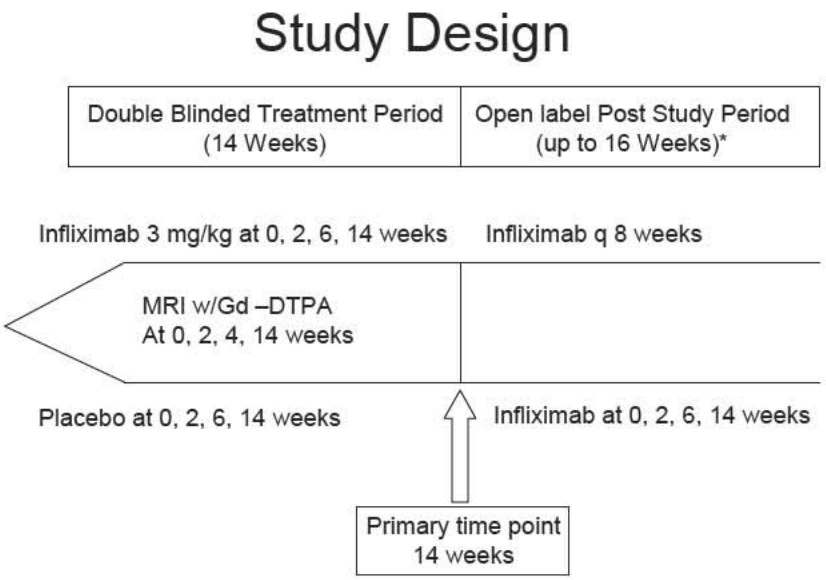

* For subjects who completed the study at Week 14 requiring infliximab treatment

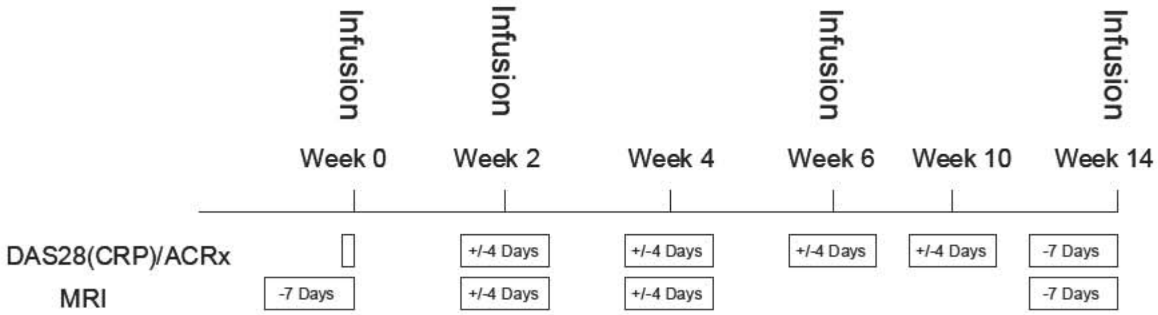

Permissible visit windows for key endpoints.

DAS28(CRP)/ACRx components* and MRI are always collected before Infusion of study medication.

* DAS28(CRP)/ACRx components: Tender Joint Count, Swollen Joint Count, CRP, Investigator Global Assessment, and Patient Assessments (Pain, Disease Status, and HAQ).

2.2 Study Flow Chart

	Screening		Double Blinded Treatment Period						Discontinuation	F/U
Visit Number	1	2	3	4	5	6	7	8	Discontinuation	Phone call
Day (Relative to First Dose of Study Drug)	-14 to -1	-7 to -1	1	14	28	42	70	98	1 to 98	112
Week	-2 to 0	-1 to 0	0	2	4	6	10	14	0 to 14	16
Clinic and MRI visit must occur within this time frame (+/-calendar days)	N/A	N/A	-7	+/-4	+/-4	+/-4	+/-4	-7	N/A	N/A
Explain Study and Obtain Informed Consent(s)[a]	X									
Review Inclusion/Exclusion Criteria	X		X							
Demographic Profile	X									
Medical History	X									
Prior Medications	X									
Physical Exam	X									
Body Height (cm) and Weight (kg)	X									
Complete Blood Count (CBC)[b]	X					X		X	X	
Chemistry panel[b]	X					X		X	X	
Urinalysis	X									
Pregnancy Test (Serum BHCG)[c]	X									
Pregnancy Test (Urine BHCG)[c]			X	X		X		X	X	
FSH Test[d]	X									
HIV/HBsAg/HCV[e]	X									
Drug Screen[e]	X									
TB Evaluation[f]	X		X	X	X	X	X	X	X	
TB screening (Skin Test or QuantiferonTB Gold) [f]	X									
Screening Number Assignment	X									
Randomization Number Assignment			X							
Provide Subject Identification Card (see 9.1.3)			X							
ECG (12-Lead)	X									
Vital Signs (BP, Pulse Rate, Temp.)[g]	X		X	X	X	X	X	X	X	
ACR Criteria for Diagnosis of RA[h]	X									
Tender Joint Count (28 joint set)[ij]	X		X	X	X	X	X	X	X	
Swollen Joint Count (28 joint set)[ij]	X		X	X	X	X	X	X	X	
Health Assessment Questionnaire (HAQ) [i]			X	X	X	X	X	X	X	
Patient Global Assessment of Disease Activity -GADP(VAS) [i]			X	X	X	X	X	X	X	

	Screening		Double Blinded Treatment Period						Discontinuation	F/U
Visit Number	1	2	3	4	5	6	7	8	Discontinuation	Phone call
Day (Relative to First Dose of Study Drug)	-14 to -1	-7 to -1	1	14	28	42	70	98	1 to 98	112
Week	-2 to 0	-1 to 0	0	2	4	6	10	14	0 to 14	16
Clinic and MRI visit must occur within this time frame (+/-calendar days)	N/A	N/A	-7	+/-4	+/-4	+/-4	+/-4	-7	N/A	N/A
Patient Global Assessment of Pain (VAS) [i]			X	X	X	X	X	X	X	
Physician Global Assessment of Disease Activity (VAS) [i]			X	X	X	X	X	X	X	
CRP[i]	X		X	X	X	X	X	X	X	
ESR[k]	X									
Rheumatoid Factor			X							
DNA Sample for Responder Analysis (Genetic Polymorphisms)			X							
RNA Sample for Responder Analysis (Gene Expression Signatures)			X							
Optional Samples for Future Use										
DNA Sample for Pharmacogenomic Analysis[l]			X							
RNA sample for Pharmacogenomic Analysis[l]			X	X	X	X	X	X	X	
Plasma Sample for Archive (miRNA profiling)[l, m]			X	X	X	X	X	X	X	
Serum Sample for Archive[l, m]			X	X	X	X	X	X	X	
Blood for high dimensionality cytometry[n]			X		X					
DCE-MRI (wrist and MCP joints)[o]		X		X	X			X	X	
Treatment Administration (IV) [p]			X	X		X		X		
Record AEs and Concomitant Meds	X		X	X	X	X	X	X	X	X

a. These include the main study consent, informed consent for pharmacogenetic sample, and future use consent.

b: On treatment days, blood samples should be drawn predose.

c: For women of childbearing potential only.

d: For post-menopausal women only.

e: Performed at investigator discretion.

f: At prestudy/screening, subjects must satisfy all TB criteria including a negative skin test with PPD or a QuantiFERON-TB Gold test (**Appendix 2**). At Weeks 0, 2, 4, 6, 10, and 14, subjects must show no signs or symptoms suggestive of active TB upon medical history and/or physical examination, and have had no recent close contact with a person with active TB or, if there has been such contact, will be referred to a physician specializing in TB to undergo additional evaluation.

g: Oral temperature measurement is preferred, but axillary temperature is also acceptable.

h. Refer to **Appendix 3**.

i. Results from these assessments will be used by the Sponsor to calculate DAS28(CRP) and ACR20, ACR50 and ACRx. At Week 2, 4, and 14, the Tender and Swollen Joint Count, CRP, GADP, and other Investigator and Patient assessments may be performed **before or after** the MRI as long as they are performed within the permissible time frame. These assessments must be performed **before** the administration of study medication.

j. Tender and Swollen Joint Counts (28 joint set) will be performed by a masked joint examiner with no other role in the clinical care of the patient. The same masked joint examiner should be used for all assessments of an individual patient. Baseline Tender and Swollen Joint Counts must occur before administration of study medication at Week 0 (Visit 2).

	Screening		Double Blinded Treatment Period						Discontinuation	F/U
Visit Number	1	2	3	4	5	6	7	8	Discontinuation	Phone call
Day (Relative to First Dose of Study Drug)	-14 to -1	-7 to -1	1	14	28	42	70	98	1 to 98	112
Week	-2 to 0	-1 to 0	0	2	4	6	10	14	0 to 14	16
Clinic and MRI visit must occur within this time frame (+/-calendar days)	N/A	N/A	-7	+/-4	+/-4	+/-4	+/-4	-7	N/A	N/A

k. At Screening, an ESR by Westergren method may be used to satisfy the inclusion criteria in **Section 7.3.1**.

l. Informed consent for pharmacogenomic samples must be obtained before the DNA, RNA, Plasma, and serum future use samples are collected. DNA sample for analysis should be obtained on Day 1 on randomized subjects only, or at a later date as soon as the informed consent is obtained.

m. Future use consent must be signed before these samples are collected.

n. High dimensionality cytometry assays are contingent upon operational availability as described in **Section 7.6**.

o. Screening MRI must be obtained within 7 days prior to Baseline and approved by Sponsor or Sponsor's agent before initiating study treatment at Week 0. If subject is eligible, the screening MRI will be used as the baseline MRI at Week 0. An MRI is required at the time of study discontinuation unless an MRI was obtained within 4 weeks prior. At the scheduled timepoints, the MRI must be obtained **before** the administration of study medication.

p. At all visits, study drug is administered **after** performance of Tender and Swollen Joint Count, serum for CRP is obtained, and after the GADP(VAS) and other investigator and patient questionnaires are completed **AND after** the MRI is collected. Study medication is prepared and hung on an IV pole with obscuring drape by an unmasked trained person with no other role in study conduct.

3 TABLE OF CONTENTS

1 TITLE PAGE 1
2 SYNOPSIS 3
2.1 Study Design Diagrams 8
2.2 Study Flow Chart 9
3 TABLE OF CONTENTS 12
3.1 List of Appendices 16
4 LIST OF ABBREVIATIONS AND DEFINITIONS OF TERMS 17
5 INTRODUCTION 20
5.1 Background 20
5.1.1 Class or Type of Drug Being Studied/Description of Drug 21
5.2 Rationale 21
5.2.1 Study Conduct Rationale 21
5.2.2 Study Design Rationale 22
5.2.3 Dose Rationale 34
6 STUDY OBJECTIVES 34
6.1 Primary Objective 34
6.2 Secondary Objectives 35
6.3 Exploratory Objectives 35
7 INVESTIGATIONAL AND ANALYSIS PLAN 36
7.1 Design of the Study/Methodology 36
7.1.1 Screening 39
7.1.2 Baseline and Treatment Visits 39
7.1.3 Study Completion/ Follow-Up 40
7.2 Participation in and Completion of the Study 40
7.3 Study Population 41
7.3.1 Subject Inclusion Criteria 42
7.3.2 Subject Exclusion Criteria 46
7.3.3 Subject Discontinuation Criteria 48
7.3.4 Replacement of Subjects 50
7.4 Treatments 50
7.4.1 Study Treatments 51
7.4.1.1 Treatments Administered 51
7.4.1.2 Timing of Dose for Each Subject 52
7.4.1.3 Method of Treatment Assignment, Randomization, and/or Stratification 52
7.4.1.4 Management of Blinding of Study Treatments 53
7.4.1.5 Investigational Product 54

7.4.1.5.1 Identity of Investigational Product(s) 54
7.4.1.5.2 Source 54
7.4.1.5.3 Labeling, Storage and Dispensing 54
7.4.1.5.4 Investigational Product Accountability 55
7.4.1.5.5 Modification of Dose and/or Administration of Investigational Product for a Subject 56
7.4.2 Non-Study Treatments 56
7.4.2.1 Prior and Concomitant Medications 57
7.4.2.1.1 Medications, Supplements, and Other Substances from Baseline and During the Study 57
7.4.2.1.2 Medications Allowed During the Study 57
7.4.2.2 Dietary, Tobacco, Alcohol, Caffeine, and Other Restrictions 58
7.4.2.2.1 Exercise 58
7.4.3 Procedures for Monitoring Subject Compliance 58
7.5 Blood Sampling 59
7.6 Study Procedures 59
7.7 Study Assessments and Analysis 68
7.7.1 Pharmacodynamics/Safety/ Pharmacogenomic 68
7.7.1.1 Pharmacodynamics 68
7.7.1.2 Interpharmacodynamic (PD-PD) and Safety-PD Analysis 70
7.7.1.3 Pharmacogenomics Analysis 70
7.7.1.4 PD/PGx, PK/PGx and Safety/PGx Analysis 71
7.7.1.5 Other Assessments 71
7.7.2 Safety Monitoring and Assessments 71
7.7.2.1 Definition of Terms 71
7.7.2.1.1 Adverse Event 71
7.7.2.1.2 Serious Adverse Event 72
7.7.2.1.3 Closely Monitored Event 72
7.7.2.1.4 Overdose 73
7.7.2.1.5 Product Quality Complaint 73
7.7.2.1.6 Planned Hospitalization 73
7.7.2.1.7 Medication Error 73
7.7.2.1.8 Potential Medication Error 74
7.7.2.1.9 Incidents 74
7.7.2.2 Monitoring 74
7.7.2.2.1 Monitoring Adverse Events 74
7.7.2.2.2 Monitoring Laboratory Assessments 75
7.7.2.3 Assessment of Adverse Events 75
7.7.2.3.1 Assessment of Severity 75

7.7.2.3.2 Assessment of Causality ... 76
7.7.2.3.3 Reference Safety Information (RSI) for the Assessment of Expectedness of Adverse Events ... 76
7.7.2.3.4 Known Potential Toxicities of Study Drug ... 76
7.7.2.3.5 Known Adverse Events Relating to the Underlying Clinical Condition ... 77
7.7.2.4 Reporting Safety Observations by the Investigator to the Sponsor ... 77
7.7.2.4.1 Expedited Reporting of Safety Observations by the Investigator to the Sponsor ... 77
7.7.2.4.2 Expedited Reporting by the Sponsor to a Regulatory Health Authority ... 79
7.7.2.5 Discontinuation, Treatment Interruption, and Unblinding of Blinded Treatment Due to Safety Observations ... 80
7.7.2.5.1 Discontinuation ... 80
7.7.2.5.2 Temporary Interruption of Treatment for a Subject ... 80
7.8 Criteria for Early Termination of the Trial ... 80
8 STATISTICAL ANALYSIS AND REPORTING PLANS ... 81
8.1 Data Sets ... 81
8.2 Demographic and Other Baseline Characteristics ... 81
8.3 Pharmacodynamic Analyses ... 81
8.3.1 Pharmacodynamics ... 81
8.3.2 Pharmacodynamic-Pharmacodynamic Analysis ... 86
8.4 Determination of Sample Size/Power/Level of Significance ... 86
8.5 Interim Analysis ... 87
8.6 Safety ... 87
8.6.1 Adverse Events ... 87
8.6.2 Clinical Laboratory Tests ... 87
8.6.3 Vital Signs ... 88
8.6.4 Physical Examination ... 88
8.6.5 Electrocardiogram ... 88
8.6.6 Other Safety ... 88
8.7 Other Analyses ... 88
9 ADHERENCE TO ETHICAL, REGULATORY, AND ADMINISTRATIVE CONSIDERATIONS ... 89
9.1 Ethical Conduct of the Study ... 89
9.1.1 Independent Ethics Committee or Institutional Review Board ... 89
9.1.2 Subject Information and Consent ... 90
9.1.3 Subject Identification Card ... 91

9.1.4 Registration of the Trial 92
9.2 Reporting Trial Data to the Sponsor 92
9.2.1 Data Collection Forms 92
9.2.2 Preparing Case Report Forms for All Subjects 93
9.2.3 Preparing Case Report Forms for Subjects Who Fail Screening 93
9.3 Publications and Other Rights 94
9.3.1 Rights to Publish by the Investigator 94
9.3.2 Use of Proprietary or Confidential Information in a Publication 95
9.3.3 Use of Trial Information in a Publication 95
9.3.4 Authorship of Publications 96
9.4 Trial Documents and Records Retention 96
10 INVESTIGATORS AND TRIAL ADMINISTRATIVE STRUCTURE 98
10.1 Sponsor 98
10.2 Investigators 98
10.2.1 Selecting Investigators 98
10.2.2 Financial Disclosure Requirement 98
10.2.3 Clinical Study Report Coordinator Investigator 99
10.3 Central Organizations 99
11 REFERENCES 100

3.1 List of Appendices

		Page
**Appendix 1**	DNA Sampling and Pharmacogenetic Analysis Procedures	108
**Appendix 2**	Tuberculosis Screening	115
**Appendix 3**	ACR Classification Criteria for Diagnosis of Rheumatoid Arhritis	124
**Appendix 4**	Health Assessment Questionnaire	126
**Appendix 5**	Infliximab Package Leaflet	129

4 LIST OF ABBREVIATIONS AND DEFINITIONS OF TERMS

Table 1 Glossary of Abbreviations and Terms

Protocol No. P08136

(S)AE	All Adverse Events, including Serious Adverse Events
ACR	American College of Rheumatology
AE	Adverse Event
AIF	Aterial Input Function
ALT	Alanine aminotransferase (SGPT)
AST	Aspartate aminotransferase (SGOT)
AUC	Area Under the Concentration-time curve
BARDS	Biostatistics and Research Decision Sciences
BHCG	Beta Human Chorionic Gonadotropin
BP	Blood Pressure
CBC	Complete Blood Count
CFR	Code of Federal Regulations
CHF	Congestive Heart Failure
CI	Confidence Interval
cLDA	constrained Longitudinal Data Analysis
CLIA	Clinical Laboratory Improvement Amendments
CRF	Case Report Form
CRP	C-Reactive Protein
CSR	Clinical Study Report
CTD	Clinical Trial Directive
DAS28	Disease Activity Score using the 28 tender and swollen joint counts
DAS28(CRP)	DAS28 calculated using C-Reactive protein
DAS28(ESR)	DAS28 calculated using Erythrocyte Sedimentation Rate
DCE-MRI	Dynamic Contrast Enhanced Magnetic Resonance Imaging
DMARD	Disease Modifying Anti-Rheumatic Drug
DNA	Deoxyribonucleic Acid
DS DNA	Antibodies to double stranded DNA
ECG	Electrocardiogram
eCRF	Electronic Case Report Form
EDC	Electronic Data Capture
EDTA	Edetic Acid
EER or REE	Early Enhancement Rate
ELSTIC	Early and Late Stage Technical Issue Committee
ERC	Ethics Review Committee
ES	Effect Size
ESR	Erythrocyte Sedimentation Rate
EU	European Union
EULAR	European League Against Rheumatism
FDA	Food and Drug Administration, USA
FOV	Field of view
FSH	Follicle Stimulating Hormone

Reporting Form 1727 and Reporting Form 1727 Completion Guide and Instructions	The Schering-Plough Research Institute collection form used to report serious adverse events (SAE) or other events to Global Pharmacovigilance (GPV). SAE information can also be provided by means of a suitable alternative as long as it contains the equivalent information and is approved by GPV.
GADP	Patient Global Assessment of Disease Status
GBCA	Gadolinium Based Contrast Agents
GCP	Good Clinical Practice
Gd-DTPA	Gadolinium with a chelating agent, diethylenetriamine penta-acetic acid, used as imaging contrast agent
GPV	Global Pharmacovigilance, A sub-unit of SPRI that is responsible for worldwide safety surveillance of all Schering marketed and investigational drugs, biologics and devices.
HAQ	Health Assessment Questionnaire
HBsAg	Hepatitis B Surface Antigen
HCV	Hepatitis C Virus
HIV	Human Immunodeficiency Virus
$IAUC_{90}$	Initial Area Under the Curve
IAUCBN90	Blood-normalized $IAUC_{90}$
ICH	International Conference on Harmonisation of Technical Requirements for Registration of Pharmaceuticals for Human Use
ICMJE	International Committee of Medical Journal Editors
IEC	Independent Ethics Committee
IFX	Infliximab
IMP	Investigational Medicinal Product
IND	Investigational New Drug Application; legal instrument in the USA that allows study of unapproved, investigational new drugs in human subjects
Investigational Product	The drug, biologic, and/or device being investigated in the current trial
IRB	Institutional Review Board
IRE	Initial Rate of Enhancement
IUD	Intrauterine Device
IV	Intravenous(ly)
K^{trans}	primary DCE-MRI parameter
LDH	Lactate Dehydrogenase
MCP	Metacarpophalangeal
ME, or RE	Maximal Enhancement, or Maximal Relative Enhancement
MRI	Magnetic Resonance Imaging
MTP	Metatarsophalangeal
MTX	Methotrexate
NA, N/A	Not applicable
NSAID	Non-steroidal Anti-Inflammatory Drug
OLT	Open Label Trial
PBMC	Peripheral Blood Mononuclear Cells
PD	Pharmacodynamics
PGx	Pharmacogenomics
PIP	Proximal Interphalangeal
PK	Pharmacokinetics
POC	Proof of Concept
PQC	Product Quality Complaint
RA	Rheumatoid Arthritis
RAMRIS	Rheumatoid Arthritis Magnetic Resonance Imaging Scoring

RBC	Red Blood Cell
RNA	Ribonucleic Acid
ROC	Receiver Operating Characteristics Curve
ROI	Region of Interest
RSI	Reference Safety Information
SAE	Serious Adverse Event
SD	Standard Deviation
SGOT	Serum glutamic oxaloacetic transaminase (AST)
SGPT	Serum glutamic pyruvic transaminase (ALT)
SJC	Swollen Joint Count
SmPC	Summary of Product Characteristics
SNPs	Single Nucleotide Polymorphisms
SOP	Standard Operating Procedure
SPRI	Schering-Plough Research Institute (the research arm of the sponsor, Schering-Plough Corporation)
SRM	Standardized Response Mean
TB	Tuberculosis
TJC	Tender Joint Count
TNF	Tumor Necrosis Factor
VAS	Visual Analog Scale
WBC	White Blood Cell

5 INTRODUCTION

5.1 Background

Evaluation of disease activity in rheumatoid arthritis (RA) is complex, with no single marker reflecting all aspects. In the past 20 years, composite disease activity instruments (e.g. DAS28) have improved the ability to evaluate the course of RA. DAS28 combines measures of swollen and tender joint counts in a 28 joint set, serum C-reactive protein (CRP), and global health questionnaire into a more responsive metric. However, DAS28 is a surrogate marker of the underlying pathological process – synovitis of the RA joint. This synovitis leads to bone and cartilage destruction, causes pain and ultimately leads to joint destruction and physical impairment.

The efficiency of proof of concept (POC) trials in RA may be improved by the incorporation of sensitive imaging biomarkers of joint synovitis to complement clinical change using validated instruments like DAS28. This is a methodology study designed to assess two MRI biomarkers to improve clinical decision-making for new drug development. Imaging endpoints that satisfy criterion validity as a measure of disease activity, that are objective, and that are at least as discriminatory as clinical endpoints to treatment effects will improve the assurance of development decisions. A placebo-controlled, randomized clinical trial to benchmark MRI biomarkers against DAS28 at time points less than 12 weeks in established RA has never been published yet potentially offers several benefits for novel drug development. First, the addition of another objective endpoint to DAS28 provides greater assurance in development decisions. Currently objective endpoints such as C-Reactive Protein (CRP) are heavily weighted in efficacy assessments, yet typically do not offer sufficient power in small, early clinical studies. Second, it may be useful to combine imaging biomarkers with DAS28 to create a composite endpoint to support smaller sample sizes or greater power with a fixed sample size. This was done in MK-0000 P-88 in prespecified fashion. Since the correlation between imaging biomarkers and DAS28 is only moderate, the introduction of new information from imaging increased the treatment difference substantially from effect size ~1 for either DAS28 or imaging biomarkers alone to about 1.6 - 2 for the composite, with the understanding that imaging composite measures with DAS28 are not fully validated. Third, it may be possible to use imaging to study treatment effects in populations with less severe disease activity, which may be important as efficacious treatments are increasingly utilized. This approach was successfully explored by identifying treatment effects using imaging biomarkers in patients enrolled in MK-0000 P88 with baseline DAS28 values at and below the median values for the enrolled population.

5.1.1 Class or Type of Drug Being Studied/Description of Drug

Infliximab, at the dose recommended for initial therapy in RA will be used in this study at a dose of 3 mg/kg with an induction regimen of 0, 2, 6 wks, and an additional infusion at 14 weeks. Infliximab is a chimeric mouse-human IgG1κ monoclonal antibody to human Tumor Necrosis Factor α (TNF) ([1]).

5.2 Rationale

5.2.1 Study Conduct Rationale

Information produced from this methodology study will be applied to the large potential pipeline for RA, a high priority disease area. The precise way it will be utilized depends on the prior level of information regarding the mechanism. Programs are expected to take advantage of a positive imaging biomarker study by either utilizing the information to reduce sample size or to improve the certainty of decisions, incorporated into Go-No-Go criteria, as long as the costs and complexity of MRI do not outweigh larger sample sizes. Beyond NO-GO decisions, imaging biomarkers may be useful to establish the clinically active dose-range. Establishing dose-response in RA trials is difficult using DAS28 and ACR endpoints. Group sizes of 40-70 are typically used, but are still not powered to statistically distinguish amongst doses. If a convincing trend is not seen, then several doses may need to be confirmed in phase 3, or additional work might be required to establish the minimally non-efficacious dose ([2,3,4]). Very few imaging studies have investigated dose response in RA. MK-0000 Protocol 088 investigated 2 doses of prednisone on clinical and power Doppler ultrasound in an adaptive design. The higher dose of prednisone had similar treatment effect on imaging biomarkers and DAS28. The lower dose of prednisone did not show statistically significant effects on either DAS28 or the prespecified imaging endpoint. However, a prespecified combination endpoint of DAS28 with ultrasound measures did demonstrate a treatment effect at 14 and 7 days, as did 3 of 8 secondary ultrasound endpoints at 14 days and 7 of 8 secondary ultrasound endpoints at 7 days, some with effect sizes of ~2, indicating substantial pharmacological activity of the 7.5 mg dose. Neither the DAS nor any individual ultrasound endpoint demonstrated statistical separation between doses, but the higher dose had numerically higher effects than the lower dose on 6 of 8 imaging endpoints and DAS28 at 14 days. In the phase 3 GO-FORWARD and GO-BEFORE studies, 50 and 100 mg of golimumab were investigated in active RA using MRI. There were no clear differences between these doses on MRI endpoints, but these doses are likely at the top of the dose-response curve for the mechanism. Finally, Kalden-Nemeth studied two doses of infliximab (1 and 10 mg/kg) and showed numerical differences between doses on a number of DCE-MRI endpoints in a

handful of patients after 4 weeks ([5]). In this study we will not investigate dose-response.

MRI is capable of identifying subclinical synovitis, implying that MRI may be uniquely suited to measure changes in disease activity in patients with lower levels of disease activity ([6]). This finding is pertinent to drug development since the activity of RA patients appears lower than in the pre-biological era ([7]). The greater availability of potent disease modifying agents will continue to challenge the ability to identify biological naïve subjects with high degrees of disease activity, the preferred population for assessment of disease activity in early clinical drug development. These trends are likely to continue; thus, identifying an imaging biomarker with sufficient dynamic range to be useful in patients lacking high degrees of symptomatic arthritis may enhance the future conduct of RA proof-of-concept studies. In support of this notion MK-0000 Protocol 088 identified treatment effects using power Doppler ultrasound in subjects with baseline DAS below the median enrolled in that small study. A similar approach can be taken in this study, though there is no plan to intentionally study patients with less active disease.

5.2.2 Study Design Rationale

This study will examine the discriminative power of several MRI measures of RA inflammation and to benchmark these imaging biomarkers to clinical activity measured using DAS28. We concentrate on inflammation because bone and joint destruction are less critical in early drug development, given the shorter periods of clinical studies during early development. A large body of work indicates synovitis and osteitis are the underlying processes that explain subsequent bone erosions in RA ([8,9]). The imaging biomarkers incorporated in this study are the Rheumatoid Arthritis MRI Score (RAMRIS) system, and parameters regarding the vascularization of synovium obtained during a time course of contrast injection, a technique called dynamic contrast enhanced magnetic resonance imaging (DCE-MRI). Moreover, this study will also demonstrate the feasibility of using DCE-MRI at multiple centers capable of enrolling rheumatoid arthritis patients to drive program decisions. Only RAMRIS scoring has been successfully employed in placebo controlled multicenter RA trials, but the shortest timepoints reported in such a rigorous study design are at 12 weeks. Therefore there is a need for methodology studies incorporating more sensitive imaging biomarkers to define the reproducibility and the treatment effect size at short time points (< 12 weeks) and benchmark these endpoints against clinical disease activity measures.

Rationale for Study Design, Population, Treatment and Effect Size

Clinical disease activity at enrollment is required for any of the imaging methods described herein to show a treatment effect, and this is also the case for improvement in clinical disease activity. RA patients have a fluctuating disease course, and enrollment of patients at an apogee of disease activity probably explains up to a third of the total DAS28 improvement in response to TNFα inhibitors ([10]). Consequently, randomized, placebo controlled studies are the only study design from which definitive determinations of efficacy can be reached. The relative performance of imaging biomarkers could certainly be explored in open label treatment studies. However, when trying to compare effect sizes of imaging biomarkers against clinical disease measures (e.g. DAS28), it is essential to have a reference control arm and patient and physician masking to treatment assignment. The available evidence suggests all the MRI imaging biomarkers mentioned here are adequately sensitive to treatment effects, and for avoidance of doubt, a placebo control arm will allow a clear decision to be made whether the cost and complexity of imaging endpoints is worthwhile in RA study paradigms. Because patients may improve in many ways with infliximab therapy, this study will use a joint examiner masked to treatment assignment to record the number of tender and swollen joints using the 28 joint set for the calculation of DAS28(CRP), but will have no other involvement in patient management, including administration of study drug or recording of AE.

Current treatment paradigms in RA emphasize prompt, thorough treatment of active disease, ideally to clinical remission, and increasingly introduced as soon as a diagnosis can be reached. True placebo controlled trials cannot be conducted. Instead patients with persistent disease activity on background treatment with DMARDs such as methotrexate (MTX) are randomized to placebo or active therapy for a short period of time. Anti-TNFα inhibitors form the most commonly prescribed, highly active initial biological therapy in such patients. Treatment effects are prompt, with CRP response by 2 weeks, swollen joint counts by 6 weeks, and a plateau in clinical response using ACR20 criteria by 14 weeks ([11]). In this study we plan to use infliximab will be administered according to a standard approved dosing regimen of 3 mg / kg at 0, 2, and 6 weeks (induction), with one additional infusion 8 weeks later at 14 weeks, which is the primary time point for the study. Infliximab is indicated in addition to methotrexate therapy. At the end of the double-blind portion of this study, the study will close. The Sponsor will make available a limited amount open-label infliximab therapy for the use of patients with their treating physician.

This study will enroll RA patients with established, active disease despite DMARD treatment, typical of those that participate in therapeutic trials of new therapeutics. Patients will be on a stable dose of methotrexate, as required for infliximab therapy. Patients will have sufficient disease activity using tender and swollen joint count criteria. Because this is a MRI study, patients will be required to have at least one wrist swollen at baseline and have evidence of synovitis, as determined by a central reader, prior to randomization.

As described below, the best validated MRI scoring system, RAMRIS, is at least as responsive to therapy as DAS28, and we hypothesize that some MRI measures are even more responsive. It is highly desirable to compare the

responsiveness of DCE-MRI biomarkers to DAS28, so we select the sample size based on DAS28. Reasonable estimates for treatment effects for infliximab treatment are derived from more recently approved TNF inhibitors or other potent biological agents in active RA patients with inadequate response to DMARDs, which are summarized in the Statistical Analysis Plan. Based on these considerations we choose to power this study based on an estimate treatment effect size of 0.7 to 0.8.

Rationale for Methods and Endpoints

The major consideration in choosing MRI endpoints to support the need of early drug development are a certain degree of criterion validation (correlation with gold-standard disease activity metrics, histological correlation, or alignment with other valid imaging modalities), and, critically, evidence that the endpoints are precise and highly discriminative of treatment effects. The MRI endpoints discussed here appear to meet these needs. Moreover, they demonstrate change with several efficacious agents, suggesting they will be general purpose tools in RA drug development.

As described below, the RAMRIS imaging biomarker system is validated and can be implemented in multicenter trials. There is abundant data in established RA at 12 weeks, but little data useful for our purposes from earlier time points. Studies in patients within 6 months of diagnosis suggest MRI may be responsive at shorter timepoints, as reported by Quinn at 4 weeks for bone marrow edema ([12]), but this population (i.e., patients with early poor-prognosis RA) is not available for study for investigational agents in early drug development. The chief deficit of RAMRIS is its ordinal scoring system, which may limit the ability to observe true, rapid improvements in synovitis. DCE-MRI shows much promise, with several reports of significant change after 1 to 4 weeks. There is a single randomized clinical trial using DCE-MRI and it is not possible to estimate a treatment effect size. It is possible that publication bias against negative studies precludes a balanced assessment of the utility of DCE-MRI in RA. If this study achieves its primary objectives there is the possibility of dramatically smaller, shorter clinical POC studies. If RAMRIS scoring shows, as expected, similar effect size as DAS28 with earliest effect detected difference at ~12 weeks, then we will accrue operational experience with MRI in RA and will have a dataset on which the franchise can optimize decision-making rules using imaging endpoints or composite clinical-imaging endpoints

RAMRIS scoring of osteitis and synovitis

The RAMRIS (Rheumatoid Arthritis Magnetic Resonance Imaging Scoring) system is the most mature semi-quantitative system for RA MRI, a well-defined, reproducible measurement system of one wrist and MCP joints suitable for multi-center use, utilize scoring of bone erosions, bone marrow edema (osteitis) and synovial volume. Cartilage loss or joint destruction or tenosynovitis is not yet a valid component of RAMRIS. The RAMRIS sequences utilize a set of T1-weighted images before and after intravenous GBCA (typically 0.1 mmol/kg) to identify enhancing synovitis. Bone marrow edema is assessed on pre-contrast fat suppressed T2-weighted coronal images. Synovitis is scored 0-3 in distal radioulnar, radiocarpal, intercarpal-carpometacarpal and 1-5 MCP joints. Osteitis is scored 0-3 in the fraction of bone involved within 1 cm of joint line of carpal bones, distal radial, distal ulnar and metacarpalbases. Expert readers examine image sets simultaneously from multiple timepoints blinded to treatment and acquisition order. Scoring can take an hour per patient-timepoint ([13]). Maximum total scores for erosion, osteitis and synovitis are 250, 75 and 24, respectively.

The reliability of RAMRIS components shows high intra-reader reliability (ICC >0.9) and in good hands, substantial inter-reader reliability ([14,15]). Comparisons in early and late RA show high correlations between MR synovial membrane thickening and enhancement post-gadolinium, and histological inflammatory changes ([16,17,18,19]). Osteitis correlates with pain, CRP and histological osteitis ([20]). Bone erosions scores using RAMRIS have been validated against radiographic skeletal erosions and are detected more sensitively in a shorter period of time. MRI using RAMRIS scoring systems has been successfully employed in numerous multicenter studies of RA therapeutics. Correlation of change with RAMRIS score with clinical status is low, suggesting that RAMRIS provides additional unique information on disease status. In the golimumab MRI dataset the correlation between change in DAS28 and change in RAMRIS synovitis score was very modest, about 0.2 (data on file).

Selected References of Treatment Effects using RAMRIS Scoring

<table>
<tr><th>Reference</th><th>Treatment</th><th>Patient No.</th><th>Design</th><th>Time points</th><th>Results</th></tr>
<tr><td>Quinn et al., 2005</td><td>Infliximab</td><td>20</td><td>RPCT</td><td>0, 4, 14, 54 wk</td><td>↓synovitis 14, 54 wk
Synovitis at 14 wk ES = 1.0
↓osteitis 4, 14, 54 wk
↓erosions 54 wk
↓DAS28 ACR CRP 14 wk</td></tr>
<tr><td colspan="6">Conducted in patients with early RA (mean duration ~6 mo), a population that is difficult to enroll. All patients started MTX at study entry, explaining some of early response from baseline. Change from baseline within group in synovitis at 0, 14, 54 wk: (IFX 5.5 to 3.4 to 3.8) vs (PBO 6.2 to 5.9 to 6.6). Note steady synovitis score for PBO. The MRI endpoints are evaluated with non-parametric statistics; ES inferred with normal approximation. The effect size for clinical change at 14 weeks were large: ACR20 = 0.84 ACR50 = 1.77 ([12]).</td></tr>
<tr><td>Østergaard et al., 2005</td><td>Anakinra</td><td>17</td><td>OLT</td><td>0, 12, 36 wk</td><td>↔ synovitis
↓DAS28</td></tr>
<tr><td colspan="6">Synovitis mean scores at 0, 12, 36 wks stable 11, 13 , 14. Osteitis mean scores stable 5, 5, 4, respectively. This small degree of change with anakinra is not unexpected given the small clinical changes in large trials ([21]).</td></tr>
<tr><td>Durez et al., 2007</td><td>Infliximab</td><td>45</td><td>RCT</td><td>0, 18, 52 wk</td><td>↓synovitis at 18, 54 wk ES=1.1
↓osteitis at 18 wk ES=1.1
↓ACR20, ACR50 at 18, 54 wk</td></tr>
<tr><td colspan="6">Patients with early RA (mean ~6 mo) were randomized to MTX, vs. MTX + methylprednisolone vs. MTX + infliximab, N~15 / group. Comparison is to MTX group. MTX only group had stable synovitis scores at 18 wk -1 (IQR 2) and bone marrow edema -2 (IQR 7). Non-parametric statistics were employed and effect size estimated from normal approximation at 18 wk. Bilateral wrist and MCP were imaged ([22])</td></tr>
<tr><td>Haavardsholm et al., 2008</td><td>Various anti-TNFs</td><td>36</td><td>OLT</td><td>0,3,6,12 mo</td><td>↓synovitis SRM = 0.79-0.92
↓osteitis SRM= 0.48-0.64
↓DAS28 SRM= 0.55-0.9</td></tr>
<tr><td colspan="6">Patients with established RA (mean disease duration 7.6 yr) and elevated DAS28 (mean 5.7) were initiated on anti-TNF and evaluated by MRI and clinical scores. SRM are change from baseline and are significant at all post-baseline times with comparable responsiveness to DAS28. Summary score of synovitis + osteitis + tenosynovitis had a SRM of 1.05 – 1.25 ([23]).</td></tr>
<tr><td>TASKi3
2009</td><td>Rigel
R788</td><td>73 PBO
146 Active</td><td>RPCT</td><td>0, 3 mo</td><td>↓synovitis 3 mo. ES=0.26
↓osteitis 3 mo ES=0.23
↔ DAS28 remission</td></tr>
<tr><td colspan="6">TASKi3 was conducted in biological non-responder population (press release JUL09). A significant treatment effect on clinical endpoints was not observed, and so not surprising that ES for MRI change is low in this trial. MRI mean scores at baseline and 3 months for synovitis (5.69, 6.04) and osteitis (6.26, 7.43) increased non-significantly.</td></tr>
</table>

ACT-RAY<br>2010	tocilizumab	63	RCT	0, 2, 12 wk	↓synovitis 2, 12 wk<br>↓osteitis 12 wk
MTX-IR either underwent ADD of Actemra or SWITCH from MTX to Actemra. As both treatments probably outperform the baseline MTX arm this study is not well-designed to estimate the treatment effect of a drug on MRI endpoints. 0.2T extremity MRI. Results are from both treatment groups combined. Change from baseline in synovitis (32% of pt) and osteitis (28% of pt) had decrease exceeding the smallest detectable change. Clinical results in substudy not available (EULAR abstract 2010).					
OPTIMA<br>2010	adalimumab	54	RPCT	0, 26 wk	↓synovitis 26 wk in ITT ES 0.53<br>↓osteitis 26 wk in ITT ES 0.73<br>↓erosion 26 wk in ITT<br>ACR20 ES 0.45<br>ACR50 ES 0.32<br>ACR70 ES 0.44
Conducted in patients with early RA (mean ~6 mo), a population that is difficult to enroll. MTX-naïve subjects with early RA were initially randomized 1:1 to receive oral MTX (escalated to 20 mg) weekly, plus either 40 mg ADA or placebo (PBO) for the first 26 weeks of treatment (period 1). Due to large MRI visit window, many patients received therapy before baseline MRI. 54 pt in ITT dataset and 36 in per protocol dataset. Mean synovitis scores with placebo 6.8 and 4.8 at 0, 26 wk. Mean osteitis scores with placebo 3.3 and 3.3 at 0, 26 wk (EULAR 2010).					
GO-BEFORE<br>2010	golimumab	318	RPCT	0, 12, 24, 52	↓synovitis 12wk ES 0.54, 0.52<br>↓synovitis 24 wk ES 0.37, 0.50<br>↓osteitis 12, 24 wk<br>↓erosion 24 wk<br>ACR50 24 wk ES 0.25, 0.17
MTX naïve. 4 arm study PBO + MTX, golimumab 100 mg, golimumab 50 mg + MTX, golimumab 100 mg + MTX. Significant changes are golimumab 50mg + MTX OR golimumab 100mg + MTX vs PBO + MTX, respectively. ES are approximated from data presented in Investigator Meeting slides MAY2010.					
GO-<br>FORWARD<br>2010	golimumab	240	RPCT	0, 12, 24, 52	↓synovitis 12wk ES 0.63, 0.42<br>↓synovitis 24 wk ES 0.62, 0.54<br>↓osteitis 12, 24 wk<br>ACR50 24 wk ES 0.54, 0.55
MTX-inadequate responder. 4 arm study PBO + MTX, golimumab 100 mg, golimumab 50 mg + MTX, golimumab 100 mg + MTX. ES are approximated from data presented in Investigator Meeting slides MAY2010.					

RPCT=Randomized placebo controlled trial, OLT = Open label trial with reader blinding, PBO = placebo, MTX = methotrexate, ES = Effect Size or the mean difference between change from baseline in treatment arms divided by the shared standard deviation at baseline. SRM=standardized response mean or mean change from baseline divided by SD of change score. Magnitude of ES or SRM: moderate (ES or SRM =0.50-0.80) or large (ES or SRM >0.80). Unless noted MRI is of wrist and MCP.

In summary, RAMRIS is capable of identifying treatment effects of potent biological drugs with effect sizes at least comparable to clinical change scores and it is straightforward to apply in multicenter clinical trials. There is no need to further validate the RAMRIS endpoint, which is included in this trial as a benchmark imaging endpoint. Adequate data with RAMRIS at ~12 weeks exist in established RA in order to estimate the additional utility of including MRI in treatment studies. There is a paucity of data at shorter timepoints in established RA.

In general it appears that RAMRIS scores in patients with established disease tend to remain steady in the control group. This behavior differs from clinical improvement which occurs within the control group. Inflammatory change appears to temporally track with improvement in clinical endpoints. Few studies have looked at time-points shorter than 3 months. Limitations of RAMRIS are the relative insensitivity to change, perhaps due to the ordinal scoring system.

Dynamic Contrast Enhanced MRI

DCE-MRI involves acquisition of sequential images every few seconds during and after the standardized intravenous injection of GBCA, typically with a power injector (0.1 mmol/kg). In the setting of inflammation the contrast material extravasates from the vascular space to interstitial space but does not penetrate viable cells. The resultant increase in T1-weighted signal intensity strongly relates to local areas of highly metabolically active synovial tissue – areas with associated extreme hypoxia, metabolic activity, angiogenesis, endothelial activation and upregulated cytokine release ([24,25]). Thus one would expect relationships between vascular leak and local tissue destruction in RA DCE-MRI directly assesses these processes by measuring the dynamics of contrast change.

In order to capture the rate of synovial enhancement, MRI protocols are chosen with short repetition times, typically a spoiled gradient echo sequence, allowing an entire image set to be acquired every few seconds to capture the dynamic enhancement phase. To maximize signal-to-noise, a small field of view is selected. In this study we will study one wrist as it is commonly involved in RA. In order to follow the same tissues longitudinally during treatment, it is recommended to obtain 3D images where signal from a slab of tissue is simultaneously obtained. The analysis of DCE-MRI is complex and typically involves segmentation of synovium from the bone.

DCE-MRI measures have been quantified by extracting such features as the rate of contrast change, often called the Early Enhancement Rate (EER in % / sec), the maximal Relative Enhancement (RE or ME) (compared with precontrast signal), and the extent of contrast washout. These are the common endpoints reported in the RA literature and are graphically illustrated:

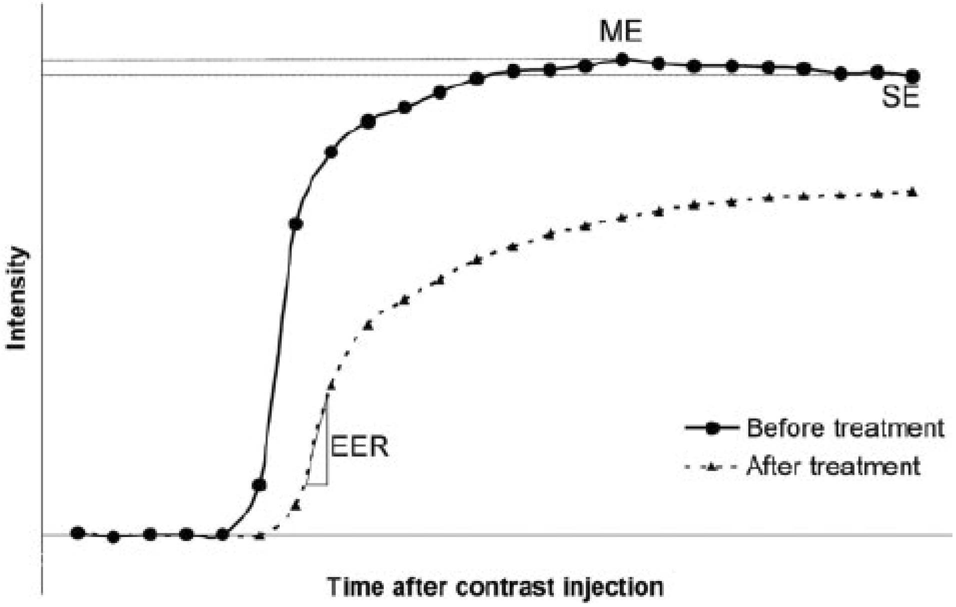

Enhancement curves from a series of DCE-MRI images of a patient with RA showing rate of early enhancement rate (EER) the maximum enhancement (ME) or Relative Enhancement (RE). Enhancement is reduced after anti-TNF-a therapy (A review by Hodgson et al.)([26]).

In an attempt to reduce variability in the relationship between observed signal and contrast agent concentrations between patients, centers and time, more rigorous approaches were developed, such as a pharmacokinetic compartment model, for instance the Toft model ([27]), that describes the exchange between the plasma space into which the Gd contrast is injected and the tissue extracellular space (the synovium). Assumptions that are required are that the relative signal enhancement curve scales linearly with contrast agent tissue concentration. The rate of exchange depends on capillary blood flow, capillary permeability and area product, volume of distribution of the contrast, and blood contrast concentration over time. The model outputs are K^{trans} (min^{-1}), the volume transfer rate from the blood plasma to the enhancing synovium; the fractional volume of enhancing synovium; and k_{ep} (min^{-1}), the volume transfer rate from the enhancing synovium to the blood plasma ([28]). In this study we will use K^{trans} in enhancing synovium as the primary endpoint. In the RA field it is not known whether the derivation of a PK compartment model will provide better sensitivity to treatment effects at the group level, but the oncology literature suggests improved precision when using individual patient arterial input functions rather than an assumed general function ([29]). One challenge of using a compartment model approach is that an arterial input function (AIF) has to be assumed or measured, for example using an automated data-driven method ([29]). Experts in DCE-MRI have reported that arterial input functions can be obtained in almost all RA patients, despite the small size of the vessels. If patient specific AIF cannot be obtained for all patients, a population AIF can be imputed. A model-free endpoint that is highly related to K^{trans} is IAUCBN90, the initial area under the Gd concentration-time curve, often for the first 90 seconds after injection, which will be used as a secondary endpoint ([29]).

Cross-sectional studies provide criteria validity for the rate of synovial enhancement on MRI. Several studies have correlated DCE-MRI with histological features. Ostergaard and colleagues found vascularity was the histology parameter with the closest correlation with the EER on DCE-MRI ([18]). The rate of maximal enhancement had very high correlation with inflammation on biopsy, while static MRI measures (e.g synovial volume) correlated more weakly with inflammation ([30]). The rate of synovial enhancement was related to fibrin exudation, cellular infiltrate, villous hypertrophy and blood vessel density but not fibrosis in RA knees. Clinical correlates (swelling tenderness or disease indices) were weak or absent ([31]). Cimmino and colleagues used a 0.2T extremity MRI to show in 36 patients with RA and 5 healthy controls that EER and maximal RE were significantly higher in patients with active RA than in those with inactive RA and controls ([32]). Szkudlarek and colleagues used DCE-MRI of the MCP to validate power Doppler ultrasound signals from the same joints. The two measures correlated with each other, but had little relationship to clinical endpoints ([33]).

Selected References using DCE-MRI in Longitudinal Clinical Studies

<table>
<tr><th>Reference</th><th>Treatment</th><th>Patient No.</th><th>Design</th><th>Time points</th><th>Results</th></tr>
<tr><td>Østergaard et al., 1996</td><td>IA methylpred-nisolone</td><td>18</td><td>OLT</td><td>0, 7, 30 d</td><td>↓ EER at 7 days -64%
↓Synovial enhancement on static MRI</td></tr>
<tr><td colspan="6">All synovium of a single slice of knee was used to measure REE. Cannot estimate clinical responsiveness as swelling or pain are either present at baseline or absent until relapse. Reduction in REE correlated with duration of clinical response. The lack of sustained response with glucocorticoid at 30 days in the group is not surprising. Usinga normal approximation and median and quartile data on figures the SRM for EER at 7 days is ~ 1.0. Same images showed a similar drop in synovial volume ([34]).</td></tr>
<tr><td>Kalden-Nemeth et al., 1997</td><td>IFX, 1, 10 mg/kg, PBO</td><td>18</td><td>RPCT</td><td>0, 4 wk</td><td>PBO No change EER
↓1 mg/kg -44% EER
↓10 mg/kg -63% EER
↓PBO → 3 or 10 mg/kg -60% EER</td></tr>
<tr><td colspan="6">Single center study part of the 4 center study that initially showed activity of IFX in RA. 4 ROI were monitored, usually wrist or knee. Change within group is from baseline. Both doses were different from placebo, but not between each other (p=0.06). ES cannot be established, but based on p values (0.001-0.005) appear as discriminative as clinical assessments ([5]).</td></tr>
<tr><td>Reese et al., 2002</td><td>MTX v. leflunamide</td><td>39</td><td>RCT</td><td>0, 4 mo</td><td>↓ EER 4 mo both treatments.
↓ ME 4 mo both treatments.</td></tr>
<tr><td colspan="6">Leflunamide had greater reduction than MTX in REE. Based on clinical performance of both treatments a difference between the two is not expected in a small sample ([35]).</td></tr>
<tr><td>Tam et al., 2007</td><td>infliximab</td><td>19</td><td>OLT</td><td>0, 14 wk</td><td>↓DAS28 0.82
↓RAMRIS synovitis 0.76
↓Vol. synovitis 0.71
↔Maximum enhancement 0.35
↓Enhancement slope 0.76</td></tr>
<tr><td colspan="6">Patients had established RA on stable doses of MTX. Maximal enhancement did not show a significant reduction in this study (p=0.07).Values presented are the SRM ([36]).</td></tr>
</table>

Hodgson et al., 2007	Infliximab	14	OLT	0, 1-2 weeks	↓ EER of synovitis<br>↓ EER of osteitis<br>↔ RAMRIS osteitis
T2-weighted images were used to measure contrast enhancement in bone marrow. Significant effects on DCE-MRI of osteitis occurred quickly after treatment, while RAMRIS osteitis did not change. Feasibility to obtain information from both synovium and bone in the same patient at one setting is uncertain. 45-68% of RA patients with active synovitis have concurrent osteitis. SRM using normal approximation: K^{trans}: 0.84; ve: 0.67; vp:1.66 ([37]).					
Fritz et al., 2009	Rituximab	10	OLT	0, 12, 26 wk	↓ EER of synovitis 26 wk<br>↔ osteitis volume<br>↓synovial volume 26 wk<br>↓ DAS28 26 and 52 wk
Rituximab used in TNF- nonresponding patients (DAS28 mean 5.4). Change from baseline is reported. Using normal approximation the SRM is ~ 0.7 for DAS28 and ~0.6 for EER at 26 wk ([38]).					

The published work is all from single-centers, suggesting that the technical protocol will need to consider issues associated with different site equipment. DCE-MRI has been developed for multicenter applications in the oncology field, and the use of phantoms has been suggested to improve the comparability of parameters between centers. We are aware of a study conducted by Amgen in 2 centers to evaluate etanercept on the effects of DCE-MRI in an open label trial (OLT) of 14 patients with RA (NCT00361634). The study, by report, was unable to identify a significant treatment effect. Questions have been raised about the magnitude of signals observed. A randomized, multicenter double blind study of tocilizumab in RA with inadequate response to anti-TNF is recruiting and uses DCE-MRI as one imaging endpoint (NCT01034397). The developers of Dynamika RA software described below are involved in multicenter studies. Therefore, it is likely that the technology can be applied across multiple centers.

Considered together, the available data suggest DCE-MRI has reasonable criterion validity and can identify changes in synovium and bone marrow edema (osteitis) due to treatment. In a number of studies, detectable changes occurred very quickly after treatment, suggesting large treatment effects. In one study, a trend towards dose-discrimination for EER suggests additional utility. The analysis methods are complicated, and improvements in measurement may also improve the discriminant capacity. The relative sensitivity of DCE-MRI endpoints to clinical endpoints will require additional experience. In this study we hypothesize that the DCE-MRI parameter, K^{trans}, will discriminate infliximab treatment effects more efficiently than DAS28 or RAMRIS synovitis.

Exploratory Endpoints

Exploratory endpoints include evaluation of the volume of synovium and DCE-MRI endpoints calculated using the Dynamika RA software. A blood RNA profile that may predict the clinical response to TNF antagonists will also be validated in this study.

Synovial volume is a continuous measure of the same feature quantified with ordinal scores in RAMRIS. The synovial membrane volume has been shown to be correlated with local clinical signs of inflammation in cross-sectional studies ([39]). The major impediment to utilizing synovial volume as a clinical trial endpoint is the analytical complexity of obtaining its measure, but semi-automated computerized methods can speed this process. The volume of enhancing synovium can be taken from MRI sequences used for RAMRIS scoring. Statistically significant reductions in synovial volume have been seen in double blind trials with time points as short as 3 months ([36,40]).

Dynamika RA software is a computer-aided semi-automated method for quantitative analysis of DCE-MRI and endpoints available in this software are included for exploratory analyses. There are four major features of Dynamika RA software. First, a motion correction algorithm is applied to remove small motions that are common in RA patients and when analysis of small structures is desired. The 3D motion correction algorithm substantially raises the number of overlapping pixels compared with 2D or un-corrected frame sets. Artifactual enhancements from tissues adjacent to high contrast voxels are reduced ([41]). Second, Dynamika applies a modeling method to each voxel with signal intensity vs. time. Each voxel is categorized in one of 4 contrast categories- no signal, continuous uptake, presence of plateau, or presence of wash-out, and assigns a color to each enhancement condition for graphical display. Third, the model on which Dynamika is based uses a noise-based approach, avoiding the use of the threshold conditions to identify enhancing synovium, and contributes to the objectivity of the results. Fourth, the parametric graphic display of the modeled contrast vs time intensity curves allows the analyst to quickly quantify the distribution and volume of inflammatory activity using the number of voxels of each contrast category. Parameters of Early Enhancement Rate (called EER) and Relative Enhancement can be computed from the models rather than average raw signal intensity vs. time curves within hand-drawn ROI. This permits the assessment of the average value in the whole wrist and metacarpophalangeal joints, largely obviating the entire issue of ROI choice. If required, the interface allows for placement of ROI for analysis and the exclusion of vessels. In addition, the number of voxels with contrast uptake, contrast plateau, and contrast washout, alone and in combination can be easily reported.

The ability to predict clinical response to infliximab is of great interest. An exploratory endpoint of this study is to assess the sensitivity and specificity of severa; blood gene expression signatures to predict, using only the baseline samples, the response at 14 weeks to infliximab as measured by DAS28(CRP). As an example, an 8-gene signature has been reported to have an 85% prediction

accuracy ([42]). Such advances require replication in other datasets. See **Section 6.3** for a list of particular gene signatures.

5.2.3 Dose Rationale

Infliximab, at the dose recommended for initial therapy in RA will be used in this study at a dose of 3 mg/kg with an induction regimen of 0, 2, and 6 weeks, and an additional infusion at 14 weeks. Infliximab is administered intravenously in 250 ml of 0.9% sodium chloride solution (normal saline) with an in-line sterile filter over a 2-hour period. This facilitates compliance and blinding of study treatment. The safety and efficacy of infliximab is well-described (SmPC). The major side effect associated with infliximab are hypersensitivity reactions and infections, including serious infections.

Infusion reactions are common and anaphylaxis may occur. Management of infusion reactions is not mandated in this protocol. However, patients who receive glucocorticoids to manage infusion reactions or for premedication to prevent infusion reactions will be **discontinued** from this study, as this may confound studying the effects of infliximab on clinical and MRI endpoints. Treatment of infusion reactions should include stopping or reducing the rate of the infusion, and optional treatment with antihistamine, acetaminophen/paracetamol, epinephrine and fluids. Several experience studies have published infusion reaction management schemes allowing almost all patients to tolerate infliximab therapy without glucocorticoids except in the case of treatment failure or angioedema, wheezing, hypotension or anaphylaxis ([1,43]).

6 STUDY OBJECTIVES

6.1 Primary Objective

To compare the effect of infliximab on synovial inflammation as measured with Dynamic Contrast Enhanced (DCE)-MRI of one wrist with placebo over 14 weeks of treatment.

Hypothesis: The change from baseline in K^{trans} in enhancing synovium with infliximab is larger than placebo over 14 weeks of therapy.

6.2 Secondary Objectives

- To compare the effect of infliximab with placebo on various MRI measures of disease activity including the change in:
  - $IAUC_{90}$ in total enhancing tissue and total enhancing synovium
  - k^{trans} in total enhancing tissue (representing largely synovium and bone)
  - Early Enhancement Rate (EER) and maximal enhancement (ME) of synovitis and osteitis of wrist
  - RA MRI Scoring (RAMRIS) of osteitis and synovitis of wrist and MCP
  - Enhancing synovial volume of wrist and/or MCP
- To compare the effect of infliximab on clinical disease activity (DAS28 (CRP), ACR20, ACR50 and ACRx) with placebo over 14 weeks of treatment. (The purpose of this objective is to assess for the possibility of a failed clinical study and to benchmark imaging biomarkers against a standard clinical biomarker.)
- To compare infliximab against placebo over 14 weeks with two composite measures of RA disease activity that incorporate DAS28(CRP) with K^{trans} or RAMRIS synovitis and osteitis,.
- To correlate changes in clinical disease activity (DAS28) and changes in MRI biomarkers of disease activity:
  - RAMRIS synovitis and osteitis
  - DCE-MRI parameters

6.3 Exploratory Objectives

- To assess the sensitivity and specificity of each of the following mRNA signatures that predict clinical response to infliximab at 14 weeks using DAS28(CRP).
  - Julia 8-gene Signature ([42])
  - Bienkowska 8-gene Signature ([44])
  - Sekiguchi 18-gene Signature ([45])
  - Tanino 300-gene Signature ([46])
  - Lequerré 8-gene Signature ([47])
  - Koczan 8-gene Signature ([48])
  - IFN 5-gene Signature ([49])

- To assess the sensitivity and specificity of each of the following genetic polymorphisms to predict, using only the baseline samples, clinical response to infliximab at 14 weeks using DAS28(CRP).
  - v-maf musculoaponeurotic fibrosarcoma oncogene homolog B (MAFB) on chromosome 20 (rs6028945 & rs6071980)
  - type I interferon gene (IFNk) on chromosome 9; (rs7046653)
  - paraoxonase I (PON1) gene on chromosome 7 (rs854555, rs854548, rs854547)
  - TNFα gene promoter, known as the 308 G/A variant (TNF-308) (rs1800629)
- To evaluate the responsiveness of endpoints available in Dynamika RA software to infliximab compared with placebo. These endpoints include EER and ME and the number of voxels showing contrast uptake, contrast plateau, and contrast washout, alone and in combination.
- To compare the effect of infliximab with placebo on synovial inflammation as measured by K^{trans} derived from DCE-MRI of all segmentable synovium in wrist and all MCP in the field of view (FOV).
- To compare the effect of infliximab with placebo on RAMRIS scoring of bone erosions in wrist and MCP.
- To compare the effects of infliximab with placebo on a measure of inflammation that sums RAMRIS osteitis and synovitis scores, and a measure of damage that sums RAMRIS bone erosion and non-RAMRIS cartilage damage.
- To compare the effect of infliximab with placebo on the volume of osteitis.

7 INVESTIGATIONAL AND ANALYSIS PLAN

7.1 Design of the Study/Methodology

Patients with active RA on a stable dose of methotrexate and with evidence of wrist synovitis on MRI that meet study eligibility criteria will participate. Patients will be randomized to receive intravenous infusion of either infliximab 3 mg/kg or placebo for 14 weeks, with infusions administered at Weeks 0, 2, 6, and 14. A MRI with intravenous GBCA taken of one wrist and MCP joints will be obtained within 7 days before the patient undergoes treatment with either infliximab or placebo and will be assessed for eligibility. Patients will be assessed for RA activity using DAS28 (an assessment of tender and swollen joints, the blood inflammation marker C-reactive protein (CRP) and patient's global assessment of disease status using a visual

analog scale (VAS)), patient assessment of pain, patient's and physician's global assessment, health assessment questionnaire (HAQ) and for adverse events before each infusion or MRI. MRI with GBCA will be obtained at Week 2, 4 and 14. The 2 week MRI may be removed from the study procedures based on Sponsor assessment of feasibility. The study will conclude after completion of procedures at Week 14. Adverse experiences will be collected though out the study, and for an additional 2 weeks afterwards. At the completion of week 14 procedures, receipt of a technically adequate MRI scan and successful resolution of safety data for each patient, the treatment assignment will be unblinded to the Investigator. The Sponsor of the study will cover the cost of open-label 3 mg / kg infliximab for the optional use of all patients completing the study in consultation with a treating rheumatologist for up to 16 weeks, as described in the infliximab dosing instructions. Patients previously assigned to placebo may receive a standard induction regimen at 0, 2, 6 and 14 weeks while patients previously assigned to infliximab may receive two additional treatments every 8 weeks. The use of infliximab during this period is optional, and the decision to treat with infliximab will be made by the patient and treating rheumatologist according to the infliximab SmPC.

Planned Flexibility in this Study

This protocol is written with some flexibility to accommodate for the inherent dynamic nature of Experimental Medicine clinical trials. The disease specific inclusion criteria, imaging endpoints, and the timing of the MRI procedure currently outlined in the protocol may be modified during the study based on newly available data. The flexibility required for these aspects of the study along with the rationale for such adaptive design are further described below.

Rheumatoid arthritis disease specific inclusion criteria are defined flexibly and may be altered for all patients going forward by the Sponsor. Specifically, the number of swollen or tender joints (28 joint set), the number of minutes of morning stiffness, CRP or ESR criteria or DAS28(CRP) threshold at screening or baseline may be altered by the Sponsor. The duration of stable DMARD and methotrexate treatment prior to baseline may be altered by the Sponsor. The presence or absence of MRI

specific criteria at baseline can be imposed, altered or removed. Specifically, the presence and severity of detectable synovitis of the wrist or MCP joints may be imposed, altered or removed by the Sponsor. Such alterations will be made by a protocol clarification letter if the study proves infeasible to run with the initially described inclusion criteria as long as the objectives of the study can be achieved.

The collection of DCE-MRI in multicenter trials has not yet been presented in the literature in RA. It may prove infeasible, either because of image quality or for patient comfort, to collect all the MRI endpoints described in this protocol. The baseline MRI scan of the first 6 to 10 patients randomized to the protocol will be investigated to determine the likelihood that the primary DCE-MRI parameter, K^{trans},, can be measured in the majority of patients. K^{trans} requires fitting the data to a compartment model, which requires an estimate of the concentration of Gd in the plasma space over time. This is called the arterial input function (AIF), and may prove difficult to measure because of the small size of the arteries in the hand. The proposed MRI sequences are lengthy, and could prove challenging for some patients with RA, though prior experience suggests the vast majority of patients will tolerate the procedure. If appropriate conditions are not met, then a population based or theoretical AIF may be imputed. If such an approach appears infeasible, then the order of other MRI endpoints ($IAUC_{90,}$ EER, ME, Dynamika and RAMRIS) may be reordered to accommodate either data with technically satisfactory collection or to accommodate shorter MRI acquisitions. This decision will be communicated by a protocol clarification letter, and changes to the analysis plan will be finalized by dated memoranda prior to breaking of the treatment blind.

Finally, the 2 week MRI may be removed from the protocol procedures based on Sponsor assessment of feasibility. Any changes made to this protocol will be detailed in a protocol clarification letter and sent to the investigators for retention. The investigators must forward a copy of this memo to the IRB/ERC. These changes will not increase the number of study procedures for a given subject during his/her participation in the entire study.

7.1.1 Screening

Within 2 weeks prior to treatment, the investigator or qualified designee shall discuss with each subject the nature of the study, its requirements, and its restrictions. Written informed consent will be obtained from each subject prior to any study-related procedures being performed, and a signed copy will be given to the volunteer. The inclusion/exclusion criteria will be reviewed, and a complete medical history, physical examination, laboratory evaluations and other assessments, delineated in **Section 2.2** and **Section 7.6** will be performed. A screening number will be assigned to each subject on completion of the study-specific informed consent.

The hand and wrist MRI with intravenous GBCA will be the last screening procedure performed. The wrist with the greatest clinical involvement at baseline will be studied throughout the trial by MRI. The screening MRI must be graded as RAMRIS synovitis ≥ 1 in either radiocarpal or intercarpal joint regions by the central reader. MRI with intravenous GBCA without adequate image quality may be repeated once, after GBCA washout. For eligible subjects, the screening MRI will serve as the baseline scan.

7.1.2 Baseline and Treatment Visits

At the Baseline visit, the investigator or designee will review the inclusion/exclusion criteria and record adverse events (AEs) and medications taken within the last 14 days. Laboratory evaluations, vital signs and other assessments, delineated in **Section 2.2** and **Section 7.6** will be performed and/or recorded. If not previously assigned, a screening number will be assigned.

The 28 joint examination at baseline by the independent masked joint examiner will serve as the basis for the computation of the baseline DAS28(CRP) endpoint.

When a subject fulfills the entry criteria, the subject will be assigned a randomization number that will be used to determine the treatment according to a randomization code (see **Section 7.4.1.3**).

7.1.3 Study Completion/ Follow-Up

On the last day of their study participation (including premature discontinuation), subjects will be discharged from the study following the collection of the last blood sample/assessment delineated in **Section 2.2** and **Section 7.6**. Approximately 2 weeks after the last study visit (including premature discontinuation), subjects will be contacted by phone to assess for potential adverse events. Subjects who completed the study at Week 14 will be offered the option to receive open label infliximab treatment following the regimen described in the infliximab product leaflet. The cost of this additional treatment is covered by the Sponsor for up to 16 weeks.

7.2 Participation in and Completion of the Study

Each subject is considered to be enrolled in the trial when the subject (or the subject’s legal representative) has provided written informed consent. All subjects will have their information entered into the screening log maintained by the study site. Those subjects, including screened subjects, who experience a serious adverse event, or have a therapeutic intervention (e.g.1, change in medication administered, dietary therapy, administration of study medication), will have information entered into the case records (e.g., eCRF).

A subject is considered to have completed the trial after he/she has completed the Week 14 joint examination, satisfactory MRI, study drug treatment and completed a follow-up contact. The follow-up contact is conducted at least 2 weeks beyond the last procedure by telephone for the collection of AE. The Week 14 MRI should be reviewed by the Sponsor or its delegate for adequate image

quality. MRI with intravenous GBCA without adequate image quality maybe repeated once, after GBCA washout.

A subject is considered to have discontinued participation after he/she has withdrawn consent or has been discontinued under the conditions specified in **Section 7.3.3**.

A subject is considered to have been lost to follow-up if the investigator is unable to contact the subject. The end of participation for a subject lost to follow-up is the last known contact (eg, visit or telephone contact).

The trial begins when the first subject is enrolled (i.e., signs the informed consent form). The trial ends when the last remaining subject has ended participation in the trial, by completing the trial, being discontinued from the trial, or being lost to follow-up.

Follow-up procedures related to pregnancy or (S)AEs may continue beyond the end of the clinical trial.

For patients completing the study assessments at Week 14, the Sponsor of the study will cover the cost of a limited supply of open-label infliximab at 3 mg/kg for optional use after the trial concludes. A patient and treating rheumatologist will elect whether or not to initiate or continue infliximab treatments. It is recommended that infliximab dosing instructions are followed in accordance with the SMPC (See also **Appendix 5**) Unused infliximab may not be used for other patients. No additional provision for infliximab is available through the Sponsor.

7.3 Study Population

Approximately 60 adult subjects with a diagnosis of Rheumatoid Arthritis will be selected for the study. Approximately 20 subjects will be enrolled at each site. There is no quota for enrollment at any given site. Subjects must meet all the inclusion criteria and none of the exclusion criteria to receive treatment assignment.

7.3.1 Subject Inclusion Criteria

The subject must meet **ALL** the criteria listed below for entry:

1. Subjects must be willing to give written informed consent for the trial and able to adhere to dose and visit schedules.

2. Subjects must be willing to give written informed consent for pharmacogenomic testing, and able to adhere to applicable visit schedules.

   Note: Subjects who are unwilling to sign the informed consent for pharmacogenomic testing may be included into the trial, however, pharmacogenomic samples must not be obtained.

Demographics

3. Subjects of either gender and of any race ≥18 years of age.

Medical History

4 Subjects must have a clinical diagnosis of rheumatoid arthritis based on the 1987 ACR clinical criteria for at least 6 months (**Appendix 3**).

   Note: Newly diagnosed subjects must have had clinical symptoms consistent with rheumatoid arthritis for at least 6 months as assessed by medical history and must fulfill the clinical criteria specified in **Appendix 3.**

5. Subjects must satisfy the following disease activity criteria:

   a. At both the prestudy/screening visit AND the baseline visit prior to randomization:

   - At least 6 tender joints AND 6 swollen joints (28 joint set: Finger Proximal Interphalangeal Joints (8), thumb interphalangeal joint (2), metacarpophalangeal (MCP) (10), wrists (2) (includes carpometacarpal, intercarpal, and radiocarpal), elbows (2), shoulders (2), and knees (2))
   - The wrist with the greatest clinical involvement by swelling or tenderness at baseline will be studied throughout the trial by MRI. (Note: this is not an inclusion criterion but is the criterion for wrist selection).

   b. At the prestudy/screening visit:

   C-reactive protein ≥ 1.0 mg/L, ***OR*** ESR ≥ 28 mm/hr

c. The baseline MRI must show evidence of synovitis in the wrist, scored as RAMRIS ≥ 1 in radio-carpal OR intercarpal joint regions as determined by the central reader.

6. Subjects must have screening laboratory tests (complete blood count [CBC], blood chemistry, and urinalysis) within the following parameters:

a. Estimated creatinine clearance of >60 mL/min based on the Cockcroft-Gault equation; the Cockcroft-Gault equation is (for females multiply result by 0.85):

$$Cl_{Cr} = \frac{(140\text{-age[yr]})(\text{body wt [kg]})}{(72)(\text{serum creatinine [mg/dL]})}$$

When creatinine is measured in micromole/litre, use the following formula:

$$Cl_{Cr} = \frac{(140\text{-age[yr]})(\text{body wt[kg]})}{(72)(\text{serum creatinine [micromol] x } 0.0113)}$$

An actual creatinine clearance, as determined by a 24-hour urine collection, may be used in place of, or in conjunction with, the Cockcroft-Gault equation.

b. Hemoglobin (hgb) >8 g/dL

c. White blood cells (WBC) > 3.5 x 10^3 cells/uL

d. Aspartate aminotransferase (AST) and alanine aminotransferase (ALT) ≤ 2.5 times upper limit of normal.

7. The Screening 12 lead ECG must be clinically acceptable.

Contraception/Post Menopausal Language

8. Women of childbearing potential and all men must agree to use a medically accepted method of contraception prior to entering the study (beginning at least 2 weeks prior to administration of the first dose of study drug, throughout the trial and for 6 weeks after the completion of the trial (at Week 14)). Acceptable methods of contraception include condoms (male or female) with or without spermicide, diaphragm or cervical cap with spermicide, medically prescribed intrauterine device (IUD), oral or injectable hormonal contraceptive, and surgical sterilization (e.g. hysterectomy, tubal ligation for women, and vasectomy for men). Women of non-childbearing potential (menopausal) do not have to use any contraceptive methods (menopause is defined as the time when there have been no menstrual periods for 12 consecutive months, with an FSH value in the postmenopausal range at the prestudy/screening visit, and no other biological or physiological cause can be identified).

9. Women of childbearing potential must demonstrate a serum β-hCG level consistent with the non-gravid state at the prestudy/screening visit.

Tuberculosis Screening

10. Subjects are considered eligible according to the following TB screening criteria:

    a. Have no history of latent or active TB prior to screening.

    b. Have no signs or symptoms suggestive of active TB upon medical history and/or physical examination.

    c. Have had no recent close contact with a person with active TB or, if there has been such contact, will be referred to a physician specializing in TB to undergo additional evaluation and, if warranted, receive appropriate treatment for latent TB prior to or simultaneously with the first administration of study agent.

    d. Within 6 weeks prior to the first administration of study agent, either have negative diagnostic TB test result according to local standards and laws (e.g. a negative tuberculin skin test or a negative QuantiFERON-TB Gold test) (**Appendix 2**).

    e. In the case of a positive TB-test without evidence of active TB, the patient is diagnosed with latent TB. Patients with latent TB can participate in this trial only if the patient agrees to and is able to receive TB prophylaxis according to local standards starting from 2 weeks prior to Baseline. **Note:** Treatment of latent TB will extend beyond the

duration of this protocol. As an example the United Kingdom recommends 6 months treatment with isoniazid 300 mg daily for 6 months. In the case of isoniazid intolerance, rifampin can be an alternative. Follow local standards and laws. The Sponsor will not provide isoniazid treatment.

f. Have a chest radiograph (both posterior-anterior and lateral views), taken within 3 months prior to the first administration of study agent and read by a qualified radiologist, with no evidence of current active TB or old inactive TB.

Concomitant Medication

11. Subjects have received methotrexate therapy for ≥ 3 months prior to the Baseline visit. The dose of methotrexate must be stable for ≥ 8 weeks prior to Baseline. In addition, subjects on methotrexate must also be taking a stable dose of folate for ≥ 4 weeks prior to Baseline. Subjects receiving ≤ 7.5 mg methotrexate (alone or in combination with other DMARDS) must have received methotrexate therapy for ≥ 8 weeks and at a stable dose for at least 8 weeks prior to baseline.

12. Subjects taking the following DMARDs in combination with methotrexate must be on a stable dose for the duration outlined below:

    a. For subjects taking D-penicillamine, hydroxychloroquine, chloroquine, oral or parenteral gold, sulfasalazine, mycophenolate mofetil, the dose must be stable for ≥ 6 weeks prior to the Baseline visit

    b. For subjects taking leflunamide, the dose must be stable for ≥ 8 weeks prior to the Baseline visit.

    c. For subjects taking Cyclosporine A and/or azathioprine, the dose must be stable for ≥ 12 weeks prior to the Baseline visit.

    Note: Any changes in stable doses of DMARDs during the course of the trial will result in discontinuation of the subject from the trial at the discretion of the SPONSOR medical monitor.

13. Subjects taking oral corticosteroids must be on a stable dose equivalent to ≤ 10 mg of prednisone (or prednisolone) per day for ≥ 2 weeks prior to the Baseline visit.

    Note: Treatment with oral corticosteroids *cannot* be initiated within the 2 weeks prior to Baseline.

14. Subjects taking ***daily*** NSAID must be on a stable dose for ≥ 2 weeks prior to the Baseline visit. The dose of NSAID must remain stable throughout the trial and until completion of trial at Week 14.

15. Subjects taking NSAID for pain on an as-needed basis must agree to discontinue NSAID use for at least 3 days and use only paracetamol for breakthrough pain for 3 days before each MRI and clinic visit (at Baseline, Weeks 2, 4, and 14). Further, these subjects must withhold taking any pain medication (including paracetamol) for at least 12 hours prior to each MRI and clinic visit.

16. Subjects who have received biological therapies (infliximab, etanercept, adalimumab, or pegsunercept) may participate, as long as the last dose of these drugs was received ≥ 3 months prior to the Baseline visit ***AND*** the reason for discontinuations was not for safety considerations ***OR*** lack of efficacy.

Diet/Activity/Other

17. Subjects agree to avoid unaccustomed strenuous physical activity (e.g., unaccustomed weight lifting, initiation of new physical therapy program) for at least 3 days before each MRI or clinic visit (at Baseline, Weeks 2, 4, and 14).

18. Subjects are able to understand and complete study questionnaires (including the use of a visual analog scale [VAS] to report responses).

7.3.2 Subject Exclusion Criteria

The subject will be excluded from entry if **ANY** of the criteria listed below are met:

1. Female subjects who are pregnant, intend to become pregnant, or are breastfeeding.

2. Subjects with inflammatory arthritis other than rheumatoid arthritis (e.g., psoriatic arthritis, systemic lupus erythematosus, spondyloarthropathy, or polymyalgia rheumatica).
3. Subjects with uncontrolled hypertension.
4. Subjects with moderate or severe congestive heart failure.
5. Subjects with a history of *or* current signs and/or symptoms of severe, progressive, or uncontrolled renal, hepatic, hematological, gastrointestinal, endocrine, pulmonary (eg, pulmonary insufficiency), cardiac, neurological, cerebral, or psychiatric disease.

6. Subjects with a history of demyelinating disease or symptoms suggestive of multiple sclerosis or optic neuritis.
7. Subjects with a history of any illness that, in the opinion of the study investigator, might confound the results of the study or pose an additional risk to the subjects by their participation in the study.
8. Subjects who had a major surgery, donated or lost one unit of blood (approximately 500 mL) within 4 weeks prior to the prestudy/screening visit.
9. Subjects who are currently participating in another clinical study or have participated in a clinical study (e.g., laboratory or clinical evaluation) within 4 weeks prior to the prestudy/screening visit.
10. Subjects who are positive for hepatitis B surface antigen, hepatitis C antibodies or HIV.
11. Subjects who are currently regular users (including “recreational use”) of any illicit drugs or have a history of drug (including alcohol) abuse within the 2 years prior to the prestudy/screening visit.
12. Subjects with a history of any tumor with the exception of adequately treated basal cell carcinoma or carcinoma in situ of the cervix.
13. Subjects with a history of any latent or active granulomatous infection including histoplasmosis, or coccidiomycosis.

    Note: Refer to **Section 7.3.1** for TB specific criteria.
14. Subjects had a non-tuberculous mycobacterial infection or opportunistic infection (eg cytomegalovirus, Pneumocystis carinii, aspergillosis) within 6 months prior to the prestudy/screening visit.
15. Subjects had a history of an infected joint prosthesis which has not been removed or replaced.
16. Subjects with a known hypersensitivity to human immunoglobulin proteins or other components of infliximab.
17. Subjects had received rituximab or natalizumab.
18. Subjects who are part of the study staff personnel or family members of the study staff personnel.

19. Patient has known claustrophobia or other contraindication to MRI. Refer to the local MRI exclusion criteria.

20. Subjects who have received any treatment listed in **Table 2** more recently than the indicated washout period prior to the Baseline visit, which, in the opinion of the investigator and sponsor, interferes with their ability to participate in the trial.

Table 2 Prohibited Medications for Entry into the Study

Prohibited Medications	Washout Period Prior to Baseline
Intra-articular injections of glucocorticoids to the wrist or MCP joints	3 months
Intra-articular corticosteroids at any site other than wrist or MCP; IM, or IV corticosteroids, including adrenocorticotropic hormone	4 weeks
Vaccination with live or live attenuated vaccines[↑]	2 weeks
Anakinra	4 weeks
Alefacept or efalizumab	3 months
Abatacept or tocilizumab	4 months
Cytotoxic agents, including chlorambucil, cyclophosphamide, nitrogen mustard, or other alkylating agents	3 months
Any investigational drug	5 half-lives

[↑] Subjects on methotrexate should not receive live or live-attenuated vaccines for at least 6 months after last study dose (at Week 14) unless recommended by a relevant specialist physician.

7.3.3 Subject Discontinuation Criteria

Subject participation may be terminated during the study for any of the following reasons:

- Serious or life-threatening adverse event (AE);
- Failure to comply with the dosing, evaluations, or other requirements of the study;
- Request of the subject (subjects have the right to discontinue treatment at any time for any reason);
- Pregnancy;
- Administrative (eg, study termination);

- An AE that occurs during or within 1 hour following an infliximab infusion, resulting in bronchospasm with wheezing, dyspnea requiring ventilatory support, or hypotension with a >40 mmHg decrease in systolic blood pressure that is associated with symptoms;
- A severe or serious delayed hypersensitivity (serum sickness-like) reaction (ie, a reaction following infliximab readministration consisting of myalgia and/or arthralgia with fever and/or rash [that does not represent signs and symptoms of other recognized clinical syndromes] or serum sickness occurring 1 to 14 days after an infliximab infusion, and which may be accompanied by other events including pruritus, facial, hand, or lip edema, dysphagia, urticaria, sore throat, and/or headache);
- Subject receives glucocorticoids for the prevention or treatment of an infusion reaction;
- Any lymphoproliferative disorder or malignancy, including skin cancer;
- Subject receives a live viral vaccination;
- New onset CHF;
- An opportunistic infection;
- A demyelinating neurological disorder;
- A diagnosis of active TB is made or suspected.

It is the right and the duty of the investigator to interrupt treatment of any subject if he/she feels that study discontinuation is necessary to protect the subject, or that there are unmanageable factors, that may interfere significantly with the study procedures and/or the interpretation of results.

If a subject prematurely discontinues, or is discontinued from the study, the primary reason for the discontinuation will be obtained and recorded on the eCRF. At the time of discontinuation, every effort should be made to perform all procedures (including the MRI evaluation) scheduled for the final visit (see **Section 2.2**, Study Flow Chart and **Section 7.6**, Study Procedures). The final MRI may be omitted if the previous MRI was obtained within 4 weeks before discontinuation.

At a minimum collect the following information when a subject discontinues:

1. The reason the subject discontinued;

2. The date of the last dose of study medication from the trial;

3. The date of the last assessment and/or contact. A follow-up contact (telephone or visit) will be arranged as appropriate;

4. (Serious) Adverse events;

5. Compliance with the test product administration as specified in this protocol;

6. Final Assessments: all procedures and evaluations scheduled for the final trial visit (**Section 2.2**, Trial Flow Chart)

7.3.4 Replacement of Subjects

After consultation between the sponsor and the principal investigator, enrollment may be extended to replace subject(s) discontinued during the study.

7.4 Treatments

Subjects randomized to infliximab will receive infliximab 3 mg/kg by intravenous (IV) infusion over a 2-hour period at Week 0, Week 2, Week 6, Week 14. Subjects randomized to placebo will receive 0.9% sodium chloride (normal saline) by intravenous (IV) infusion over a 2-hour period at Week 0, Week 2, Week 6, Week 14.

Subjects will continue to receive their standard dose and regimen of DMARDS (e.g. methotrexate and folate), NSAID or COXibs, and/or glucocorticoid (e.g. prednisone as long as dose is ≤ 10 mg per day). However, patients who receive glucocorticoids to manage infusion reactions or for premedication to prevent infusion reactions will be discontinued from the study.

Subjects complete the double blinded study period at Week 14. . At the completion of week 14 procedures, receipt of a technically adequate MRI scan and successful

resolution of safety data for each patient, the treatment assignment will be unblinded to the Investigator. The Sponsor of the study will cover the cost of open-label 3 mg / kg infliximab for the optional use of all patients completing the study in consultation with a treating rheumatologist for up to 16 weeks, per dosing instructions on the product label (see **Appendix 5**). Patients previously assigned to placebo may receive a standard induction regimen at 0, 2, and 6 weeks along with an additional infusion at 14 weeks while patients previously assigned to infliximab may receive two additional treatments every 8 weeks. The use of infliximab during this period is optional and the decision to treat with infliximab will be made by the patient and treating rheumatologist according to the infliximab SmPC.

No safety data will be collected by the Sponsor from this open-label administration of infliximab.

7.4.1 Study Treatments

7.4.1.1 Treatments Administered

Treatment A: Infliximab 3 mg / kg at weeks 0, 2, 6, 14

Treatment B: 0.9% sodium chloride at weeks 0, 2, 6, 14

Infliximab will be prepared by a trained person, unmasked to study treatment, with no other role in study conduct. Infliximab is supplied as a lyophilized vial. Each vial contains 100 mg. Infliximab is reconstituted with 10 mL of sterile water for injection, resulting in a concentration of 10 mg/mL infliximab. After addition of sterile water, swirl, DO NOT SHAKE. Infliximab 3 mg / kg diluted in 250 ml of 0.9% sodium chloride (normal saline),which may have a slightly hazy appearance. The patients weight (kg) is multiplied by the dose (3 mg / kg) to yield the desired amount of infliximab to administer, which is diluted in 250 ml of 0.9% sodium chloride (normal saline).

Example: A 70 kg person requires 70 kg * 3 mg/ kg = 210 mg of infliximab. As each vial contains 100 mg, 3 vials would be reconstituted to 10 mg / ml. The contents of 2 vials (200 mg) and 1 ml of the third vial (10 mg) would be transferred by the unblinded trained person to a 250 ml bag of 0.9% sodium chloride.

Placebo is a 250 ml bag of 0.9% sodium chloride.

The bag is hung on an IV pole and covered by a concealing drape. The IV tubing will also be covered by a concealing drape. Infliximab or placebo is administered intravenously via a filter. Infusions of infliximab should be administered over at least 2 hours. The administration of study drug is performed by study personnel blinded to treatment assignment.

No other drugs should be administered simultaneously in the same intravenous line as infliximab.

Infusion Reactions

Infusion reactions are common and anaphylaxis may occur. **However, patients who receive glucocorticoids to manage infusion reactions or for premedication to prevent infusion reactions will be discontinued from this study.** Treatment of infusion reactions should include stopping or reducing the rate of the infusion, and optional treatment with antihistamine, acetaminophen/paracetamol, epinephrine and fluids. Several experience studies have published infusion reaction management schemes allowing almost all patients to tolerate infliximab therapy without glucocorticoids except in the case of treatment failure or angioedema, wheezing, hypotension or anaphylaxis (LeQuerre et al., 2006) (SmPC).

7.4.1.2 Timing of Dose for Each Subject

Subjects will receive study medication in the clinic. At the Baseline visit, it is critical that study medication **be administered after** the collection of a technically adequate hand and wrist MRI; samples for laboratory evaluation and clinical assessments for the computation of DAS28(CRP) at baseline should also be performed prior to initiating study treatment. See **Section 2.2** and **Section 7.6**.

7.4.1.3 Method of Treatment Assignment, Randomization, and/or Stratification

The study will be stratified by center. Stratified randomization with permuted block block allocation within each stratum will be used and the allocation schedule will be

generated according to BARDS ELSTIC guidance (Guidance on Stratified Randomization at Design Stage).

On Day 1, volunteers who have met all of the eligibility requirements indicated in **Section 7.3.1 and 7.3.2** will be assigned a randomization number by the site's unblinded personnel. Subjects will then receive the study treatment corresponding to their assigned randomization number.

Randomization Nos. 001 to 060 will receive Treatment A or Treatment B.

The randomization number will be recorded in the study case record (eg, eCRF).

The unblinded trained person will be provided with the randomization number to be assigned to each replacement that corresponds to the same treatment as that received by the subject being replaced.

7.4.1.4 Management of Blinding of Study Treatments

Blinding will be preserved for the infliximab and placebo infusion treatments. Infliximab and placebo infusion treatments will be blinded to the investigator, study staff, and subject. The infusions will be prepared (according to the randomization schedule provided by the sponsor) by an unblinded trained person at each study center in accordance with **Section 7.4.1.1**, Treatments Administered. The unblinded trained person may not be involved with the study procedures, assessments, or data recording and will not reveal the randomized code to anyone. If the investigator or the sponsor's project physician/director must know immediately what treatment the subject received in this blinded study in order to provide adequate medical care, the code for that subject may be broken (ie, provided to the investigator/project physician/director by the unblinded trained person. The reason for breaking the blind is to be recorded in the study file and in the general comment section of the subject's eCRF. The sponsor must be notified immediately (if not prior to) the reason for breaking the blind. At study close, the random code should not be

returned to the sponsor, but remain at the investigational site as part of the essential trial documents.

7.4.1.5 Investigational Product

The investigator shall take responsibility for and shall take all steps to maintain appropriate records and ensure appropriate supply, handling, storage, distribution, and usage of these materials in accordance with the protocol and any applicable laws and regulations.

The following Investigational product(s) will be used in the study and will be supplied in sufficient quantities for subjects and any replacement subjects.

7.4.1.5.1 Identity of Investigational Product(s)

Refer to the SmPC and **Appendix 5** for a description of the investigational drug product(s).

7.4.1.5.2 Source

Infliximab will be purchased by the site from the same batch/lot if possible. The site will be responsible for recording the lot number, manufacturer, and expiry date of any locally purchased drugs and for forwarding this information along with the package insert to the sponsor.

7.4.1.5.3 Labeling, Storage and Dispensing

Study drug supplies must be stored in a secure, limited-access location under the storage conditions specified on the drug supply label.

Receipt and dispensing of study medication must be recorded by an authorized person at the investigator's site.

The investigator agrees neither to dispense the study drug from, nor store it at any site(s) other than those listed on the Form FDA 1572 or Investigator's Agreement. The investigator agrees that study drug(s) will be dispensed by the investigator or subinvestigator(s) named on the Form FDA 1572 or Investigator's Agreement, or their qualified designees. The investigator, subinvestigators, or qualified designees also agree that the study drug(s) will be dispensed only to study subjects who have provided written informed consent, and have met all entry criteria. Clinical supplies may not be used for any purpose other than that stated in the protocol.

An adequate quantity of study drug will be provided to replace units damaged in shipment/handling, to replace subjects meeting the entry criteria.

7.4.1.5.4 Investigational Product Accountability

Accurate and current accounting of the dispensing and return of investigational product(s) will be maintained on an ongoing basis by a member of the trial site staff:

- Investigational medicinal product(s) dispensed to each site will be recorded in the trial-specific Site Investigational Medicinal Product (IMP) Accountability Log (or equivalent document approved by the sponsor);
- Investigational medicinal product(s) dispensed to each subject will be recorded in the trial-specific Subject IMP Accountability Log (or equivalent document approved by the sponsor).

The Site IMP Accountability Log and Subject IMP Accountability Log will be verified by the sponsor's trial monitor. The original Site IMP Accountability Log and Subject IMP Accountability Log will be approved by the investigator and retained at the trial site and a copy supplied to the sponsor when the trial is complete.

As infliximab is an intravenous treatment, there are no test articles that the subject needs to return to the sites.

The following test articles may be discarded by the site immediately after use:

- Syringe;Partially used intravenous fluid bag

In the event the investigational product(s) destruction is arranged by the site, copies of the destruction records should be returned to the sponsor.

The sponsor's trial monitor will instruct the site on the return of all investigational product(s) supplies. A final inventory of the total amount of investigational product(s) received at each trial site against the amount used and returned must be recorded in the Site IMP Accountability Log. Inventory records must be readily available for inspection by the trial monitor and/or auditor, and open to government inspection at any time.

7.4.1.5.5 Modification of Dose and/or Administration of Investigational Product for a Subject

The dose and administration of the IMP to any subject may not be modified. If necessary a subject must be discontinued for the reasons described in **Section 7.3.3**.

7.4.2 Non-Study Treatments

Patients will continue to receive their standard dose and regimen of DMARDS (e.g. methotrexate and folate), NSAID or COXibs, and/or glucocorticoid (e.g. prednisone as long as dose is ≤ 10 mg per day). The Sponsor will not provide these medications.

Subjects taking daily NSAID must be on a stable dose for ≥ 2 weeks prior to the Baseline visit. The dose of NSAID must remain stable throughout the trial and until completion of trial at Week 14.

Subjects taking NSAID for pain on an as-needed basis must agree to discontinue NSAID use for at least 3 days and use only paracetamol for breakthrough pain for 3 days before each MRI and clinic visit (at Baseline, Weeks 2, 4, and 14). Further, these subjects must withhold taking any pain medication

(including paracetamol) for at least 12 hours prior to each MRI and clinic visit (at the timepoints listed above).

7.4.2.1 Prior and Concomitant Medications

All prior medication taken by the subject 14 days prior to treatment intervention and all concomitant therapy taken by the subject during the study are to be recorded on the eCRF. The identity of the therapy, the dose, route, and regimen, the dates started and stopped (or notation of "continuing" if that is the case), and the reason for use must be recorded. The use of any concomitant medication must relate to an AE or the subject's medical history.

7.4.2.1.1 Medications, Supplements, and Other Substances from Baseline and During the Study

The medications prohibited prior to Baseline and during the study are listed in **Section 7.3.2** with the subject exclusion criteria.

During non-confined study period, subjects should refrain from the use of nutraceuticals, vitamins at doses that exceed daily replacement requirements, and antacids.

7.4.2.1.2 Medications Allowed During the Study

The following medications, supplements, and other substances are allowed during the study:

Table 3 Allowed Medications

Protocol No. P08136

Medication Names
Acetaminophen (paracetamol)
Oral or injectable hormonal contraceptives
Medications necessary to treat adverse events or medical emergencies
Antihistamine, fluids, epinephrine, anti-hypertensives, and acetaminophen/paracetamol for infusion reactions

Infusion reactions may be managed with fluids, epinephrine, anti-hypertensives, antihistamine, glucocorticoid and/or acetaminophen/paracetamol. **However, patients receiving glucocorticoid for prophylaxis or treatment of infusion reactions will be discontinued from the trial.**

Note: the use of any concomitant medication must relate to the documented medical history, prophylaxis, or an adverse event of the subject.

7.4.2.2 Dietary, Tobacco, Alcohol, Caffeine, and Other Restrictions

There are no diet, tobacco, caffeine or alcohol restrictions. A history of drug (including alcohol) abuse within the previous 2 years is an exclusion criteria (**Section 7.3.2**).

7.4.2.2.1 Exercise

Subjects should continue their normal activities and must avoid unaccustomed strenuous physical activity (e.g., unaccustomed weight lifting, initiation of new physical therapy program) for at least 3 days before each MRI or clinic visit.

7.4.3 Procedures for Monitoring Subject Compliance

Infliximab is administered under medical supervision.

7.5 Blood Sampling

The table below outlines the approximate volume of blood (± 5%) to be collected from each subject. The final volume of safety samples will be determined by the site and reflected in the EC/IRB information and Informed Consent. The Sponsor will be notified of the final volume.

		Number of Blood Draws		
**Blood Test**	**Per Collection (mL)**	**Prestudy/Screening**	**Study Treatment Period**	**Total Blood Amount (mL)**
Safety – CBC	7	1	2	21
Safety – Chemistry panel	7	1	2	21
Pregnancy test (serum HCG)/FSH	5	1	n/a	5
HIV/HBsAg/HCV screen	10	1	n/a	10
CRP	2	1	6	14
ESR	2	1	n/a	2
Rheumatoid factor	2	n/a	1	2
DNA Sample (Genetic Polymorphisms)	8.5	n/a	1	8.5
RNA Sample (Gene Expression Signatures)	2.5	n/a	1	2.5
Pharmacogenomic (DNA) analysis (future use)	8.5	n/a	1	8.5
Pharmacogenomic -PaxGene RNA (future use)	2.5	n/a	6	15
Plasma archive (EDTA)	6	n/a	6	36
Serum archive	10	n/a	6	60
High dimensionality cytometry	30	n/a	2	60
			**Total amount drawn per subject**[1]	265.5

[1]Including optional samples for future use.
NOTE: Up to an additional 50 mL of blood may be obtained for additional safety and/or pharmacodynamic measurements.

Blood sampling must be done at the indicated time points in **Section 2.2 Study Flow Chart.** In case the sample is obtained more than the criteria mentioned below, or for any other sample collection/handling issue (eg, missed sample, broken sample, inappropriate sample handling, etc.) this must be recorded in the appropriate section of the eCRF.

7.6 Study Procedures

An overview of the study is provided in the Study Design Diagram in **Section 2.1**. The Study Flow Chart in **Section 2.2** summarizes the study procedures and the timing of the procedures to be performed at each visit. Individual study procedures are described below.

For details of the procedures for assessment and reporting of AEs, see **Section 7.7.2.4**, Assessment of Adverse Events and **Section 7.7.2.5**, Reporting Safety Observations by the Investigator to the Sponsor.

In order to minimize variability of evaluations, it is preferred that the same individuals perform the same types of evaluations on the same equipment for all subjects at each study site.

A list of the brand/model number for all equipment used during the trial should be recorded and placed in the site Master file. Any deviations should be noted in the comment section of the eCRF.

- **Explain Study and Obtain Written Informed Consent**

  The investigator or qualified designee will explain the study and all study requirements to the subject, answer all of his/her questions, and obtain written informed consent before performing any study-related procedure, ***including pharmacogenomic sampling and the collection of any samples for future use***. A copy of the informed consents will be given to the subject.

- **Review Inclusion/Exclusion Criteria Including Concomitant Medications**

  The inclusion and exclusion criteria will be reviewed by the investigator or qualified designee to ensure that the subject qualifies for the study. All appropriate treatment and washout times will be discussed with the subject. All medications used during the **14** days prior to first drug administration or treatment intervention, will be recorded on the eCRF.

- **Demographic Profile**

  The demographic profile required by the study eCRF and permitted by local regulations should be entered. This information may include the subject's age (at baseline), ethnicity, gender and race.

- **Medical History**

  A detailed medical history will be obtained by the investigator or qualified designee. Subject history should include information on clinically significant family and personal history, smoking history, and history of diseases (including past history of hepatitis, allergies or reactions to medications and food, seizures, previous surgery, trauma, syncope, and arrhythmias). For rheumatoid arthritis, year of onset, duration, complications, surgeries and treatments should be recorded. Any clinically relevant changes found at any time during the study will be recorded as adverse events on the eCRF.

- **Physical Examination**

  The principal investigator or designee will perform a physical examination of the following organ systems at the times specified in **Section 2.2**, Study Flow Chart: Eyes, oropharynx, thyroid, respiratory system, cardiovascular system, abdomen, skin, extremities and reflexes.

  Clinically significant changes from the ***Screening*** physical examinations will be recorded as adverse events.

- **Body Height (cm) and Weight (kg) Measurements (cm) Without Shoes**

  Body height and weight measurements should be obtained and recorded to the nearest kg and cm on the eCRF.

- **Laboratory Tests (CBC, Chemistry, Urinalysis)**

  The following will be performed according to standard laboratory procedures. All samples should be collected prior to study drug administration.

Hematology	Chemistry	Urinalysis
RBC	Sodium	pH
Hematocrit	Potassium	Specific gravity
Hemoglobin	Chloride	Protein
Platelets	Glucose	Glucose
WBC	Blood urea nitrogen (BUN) or Urea	Ketones
Neutrophils	Creatinine	Blood
Lymphocytes	Calcium	Microscopic exam only if indicated by dipstick.
Monocytes	Inorganic phosphorus	
Basophils	Total protein	
Eosinophils	Albumin	
	AST (SGOT)	
	ALT (SGPT)	
	GGT	
	Total bilirubin	
	Alkaline phosphatase	
	Uric acid	
	LDH	
	Cholesterol	
	Triglycerides	

If during the trial any laboratory result is outside the reference range and is considered to be clinically significant by the investigator, the test should be repeated at appropriate time intervals until it returns to baseline or becomes a clinically insignificant finding. Any adverse clinically significant change will be recorded on the eCRF as an Adverse Event. All laboratory tests, including urinalysis results, will be recorded on the eCRF;

- **Pregnancy Tests and FSH**

A serum pregnancy test must be performed at the Screening visit and a urine pregnancy test must be performed at the Baseline visit for females of childbearing/conceiving potential (including females with a partial hysterectomy or tubal ligation). The urine pregnancy test is also performed periodically throughout the trial as outlined in **Section 2.2.** Results must be available prior to dosing. Subjects with a positive pregnancy test at screening or prior to the first dose will not be allowed to enter into the study (screen failure). Subjects with a positive pregnancy test anytime after first dose will be discontinued from the study. Positive results will be recorded on the Adverse Event section of the eCRF. The pregnancy will be reported and monitored according to **Section 7.7.2.5**, **Reporting Safety Observations by the Investigator to the Sponsor.**

The FSH test is performed on post-menopausal women only.at the Screening visit.

- **Test for HIV Antibodies, Hepatitis B Surface Antigen and HCV**

  Tests will be performed according to standard local procedures and are performed at the discretion of the Investigator. A plan must be in place at the site(s) for the management of a positive test result according to local requirements. Results will not be recorded on the eCRF.

- **Screen for Drugs With a High Potential for Abuse**

  Urine tests will be conducted according to standard procedures and are performed at the discretion of the Investigator. In addition to commonly abused drugs, any drugs abused locally with a high prevalence, should also be tested. Positive results will be recorded on the Comment section of the eCRF, and will be reported to the sponsor.

- **Tuberculosis Screening and Evaluation**

  Tuberculosis screening includes a review of subjects' medical history, a tuberculin skin test or QuanitferonTB Gold test (**Appendix 2**) and chest x-ray.

  Local guidelines specifically directed at high risk and immunocompromised subjects should be used for screening and interpretation of positive test results. Tuberculin skin testing must be performed and read by experienced, trained, and licensed personnel according to published local guidelines, either at the participating trial site, a local public health clinic, or a primary care physician’s office. If the test was performed at any site other than the principle investigator’s facility, the results must be made available in written form as source documentation. QuantiFERON-TB Gold testing is an acceptable alternative to tuberculin skin testing.

  In the case of a positive TB-test (either positive skin test or QuantiFERON-TB Gold test or findings on chest x-ray suggestive of latent TB such as pleural scarring), a subject can participate in this trial only if the subject agrees to receive TB prophylaxis with isoniazid 300 mg once daily starting from 2 weeks prior to Baseline.

- **Screening Number Assignment**

  - Following completion of the study-specific informed consent by the subject, a screening number will be assigned to the subject by the site. Screening numbers will be recorded on the eCRF.

- **Randomization Number Assignment**

  Prior to the administration of study treatment, a randomization number will be assigned to each volunteer by the site using the randomization numbers provided in **Section 7.4.1.3**.

- **Issue or Collect Subject Identification Card**

  The investigator or qualified designee will provide the subject with a Subject Identification Card when the subject is no longer under the direct observation of the investigator/site. The investigator or qualified designee will retrieve the card from the subject at the last contact (see **Section 9.1.3** for further description of the Subject Identification Card).

- **Clinical Assessment of Electrocardiograms**

  The principal investigator/designee will record Screening ECG data and any unscheduled ECG data in full on the eCRF.

  Local ECGs

  The 12-lead ECG (12-lead at 25 mm/sec reporting rhythm, ventricular rate, PR, QRS, and QT intervals) will be performed by the principal investigator or designee after the subject has been in the supine position for at least 5 minutes.

  A commercial 12-lead electrocardiograph capable of recording, storing, and printing on full-size paper, high resolution 12-lead data, should be used. On the electrocardiogram should be recorded the date, time, subject demographics (eg, age, and sex), subject number, and study-related information (eg, protocol number and nominal time).

- **Vital Signs**

  Vital signs will be obtained by the principal investigator or designee. Subjects will be in a semi-recumbant position for at least 3 minutes. Systolic and diastolic blood pressure (mm Hg), pulse rate (bpm) and oral body temperature (°C) and the actual clock time (24:00) will be recorded on the eCRF. Any clinically significant change from baseline for vital sign measurement (not associated with another adverse event) will be recorded on the adverse event eCRF form.

All blood pressure measurements should be made by consistently using the same arm and type of equipment such as a mercury sphygmomanometer (preferred), a recently calibrated aneroid manometer, or a validated automated electronic device, with an appropriate cuff size. Auscultatory or oscillometric technique should be used with the mercury sphygmomanometer or aneroid manometer. Pulse rate should be obtained manually by radial pulse palpation over a 30 second count or by a validated automated electronic device.

Body temperature should be measured consistently using the same approach (oral or axillary). Since drinking hot or cold water (or other beverages) has a significant impact on recorded oral temperature, hot or cold beverages should not be ingested within 15 minutes of the oral temperature measurement.

If the scheduled time for vital sign measurements coincides with a blood collection, the vital signs should be performed prior to the blood collection, or at least 5 minutes afterwards. This same timing for obtaining the vital signs (before or after the blood collection) should be used for all vital sign measurements.

- **ACR Criteria for Diagnosis of RA**

  Subjects will be evaluated against the ACR Criteria through an assessment of medical history, physical examination, laboratory and radiographic findings. See **Appendix 3** for the ACR criteria.

- **Swollen and Tender Joint Count**

  The 28 joint count for pain and swelling will be used. The following 28 joints will be assessed: Finger Proximal Interphalangeal Joints (8), thumb interphalangeal joint (2), metacarpophalangeal (MCP) (10), wrists (2) (includes carpometacarpal, intercarpal, and radiocarpal), elbows (2), shoulders (2), and knees (2). Tender and Swollen Joint Counts will be performed by a masked joint examiner with no other role in the clinical care of the patient. The same masked joint examiner should be used for all assessments of an individual patient. Tender and Swollen Joint Counts must occur before initiating study treatment at the relevant visits.

- **Health Assessment Questionnaire (HAQ)**

  Subjects will complete the Health Assessment Questionnaire (HAQ). See **Appendix 4**.

- **Patient's Global Assessment of Disease Activity (GADP) Visual Analog Scale (VAS)**

  Subjects will be asked to make an overall global assessment of their disease activity on a VAS (scale of 0 to 100 mm, with 0 being very well to 100 being very poor).

- **Patient's Global Assessment of Pain Visual Analog Scale (VAS)**

  Subjects will be asked to make an overall global assessment of pain due to their disease during the past week on a VAS (scale of 0 to 100 mm, with 0 being no pain to 100 being extreme pain).

- **Physician's Global Assessment of Disease Activity Visual Analog Scale (VAS)**

  Physician's global assessment of disease activity will be recorded on a VAS (scale of 0 to 100 mm, with 0 being very well to 100 being very poor).

- **CRP, ESR, Rheumatoid Factor**

  An ESR by Westergren method or the CRP maybe used to satisfy the inclusion criteria outlined in **Section 7.3.1**. The CRP is also used to compute the DAS28(CRP). Laboratory tests for CRP and rheumatoid factor will be performed by the central laboratory.

- **Samples for Responder Analyses of Genetic Polymorphisms and Gene Expression Signatures**

  Blood samples are required from all randomized subjects for analyses of DNA polymorphisms and blood mRNA signatures. An 8.5 mL blood sample (for DNA) and a 2.5 mL blood sample (for RNA) are collected at baseline.

- **Pharmacogenomic (PGx) Samples**

  Informed consent specific for PGx sampling must be obtained prior to collection. To obtain sufficient DNA, RNA, Serum, Plasma for pharmacogenomic studies, a single 8.5 mL blood sample, 6 x 2.5 mL RNA samples, 6 x 6 mL Plasma samples, 6 x 10 mL Serum samples will be drawn at the specified time point indicated in **Section 2.2**, Study Flow Chart, into the appropriate tube provided by the sponsor (see **Appendix 1** for additional information on PGx sample management).

- **Serum Samples for Determination of Pharmacodynamic Responses (Future Use)**

  Serum samples are collected for archive at the specified time points indicated in **Section 2.2**, Study Flow Chart. Informed consent for future use must be obtained before these samples can be collected.

- **Samples for High Dimensionality Cytometry Cell Stimulation and High-Dimensionality Flow Cytometry**

  This high dimensionality cytometry assay involves stimulation of peripheral blood mononuclear cells with certain stimulants (e.g. cytokines, TLR ligands, etc.) followed by high-dimensionality flow cytometry on the stimulated cells. The method emphasizes measurement of activation states of proteins thought to be nodes in the signaling networks of various gated cell populations.

  While the method is labor intensive and technically complex, published results ([50]) and preliminary results (based on internal discussions with PPD and Nodality) suggest the method may have unique promise as a predictive platform for subtle immunologic phenomena. Furthermore, Nodality has validated SOPs for sample collection, shipping, processing, and assay; they also have a CLIA-certified laboratory.

  The site based procedure involves drawing blood from the patient. Further, the site must be able to process blood to generate and store peripheral blood mononuclear cells (PBMC). These assays may not be incorporated into the study since they are contingent on factors unable to be resolved at the time of protocol authorship.

- **Dynamic Contrast Enhanced Magnetic Resonance Imaging**

  One wrist and MCP joints will be imaged with MRI using GBCA at various timepoints in this trial. The first MRI will be performed during Screening, as part of the review of inclusion criteria. This MRI must show evidence of synovitis in the wrist, as determined by the central reader. For eligible subjects, the screening MRI will also serve as the baseline scan. **The same wrist and MCP joints will be followed and imaged at the subsequent visits.** The MRI should be performed prior to study drug administration at the relevant visits.

  For more detailed information on the acquisition of the MRI images, see the MRI manual.

- **Treatment Administration (Infliximab or Placebo Infusion)**

  Refer to **Section 7.4** for Treatments to be administered. The time of dose administration should be recorded in eCRF.

- **Record Adverse Events and Concomitant Medications**

  See **Section 7.7.2.4**, Assessment of Adverse Events, **Section 7.7.2.5**, Reporting Safety Observations by the Investigator to the Sponsor, **Section 7.4.2**, Non-study Treatments and **Section 7.3.3**, Subject Discontinuation Criteria.

7.7 Study Assessments and Analysis

7.7.1 Pharmacodynamics/Safety/ Pharmacogenomic

7.7.1.1 Pharmacodynamics

The following endpoints are studied:

Disease Activity, DAS28(CRP): components are the number of tender joints (28 joint count), the number of swollen joints (28 joint count), a Global Health index (100 mm VAS), and CRP (mg/L). The formula for determining DAS28(CRP) is as follows:

DAS28(CRP) = $0.56*\sqrt{}(TJC28) + 0.28*\sqrt{}(SJC28) + 0.36*\ln(CRP+1) + 0.014*GADP(VAS) + 0.96$,

with the following 28 joints: Finger Proximal Interphalangeal Joints (8), thumb interphalangeal joint (2), metacarpophalangeal (MCP) (10), wrists (2) (includes carpometacarpal, intercarpal, and radiocarpal), elbows (2), shoulders (2), and knees (2). Joint counts are performed by by independent masked examiner.

American College of Rheumatology responder criteria (ACR20): requires that both tender and swollen joint counts improve by at least 20% from baseline during the course of a trial, as well as 20% improvement of at least 3 of the 5 other ACR/European League Against Rheumatism (EULAR) core set measures (pain, patient's and physician's global assessment, physical disability (HAQ), and CRP).

Similar definitions are used for ACR50 and ACRx where x is between 5 and 45. There is also a continuous form of ACRx that measures the minimum of the percentage improvement of the number of tender joints, number of swollen joints, and the median percentage improvement of the five EULAR core set measures.

<u>Scoring of Synovium, Osteitis, Bone Erosion, and Cartilage Damage:</u>

- RAMRIS synovitis is scored 0-3 in 8 locations (0-24 total)
- RAMRIS osteitis is scored 0-3 in 25 locations (0-75 total)
- RAMRIS erosion is scored 0-10 in 25 locations based on the percentage of volume of bone eroded 1= 1-10%, 2=11-20%, etc); Total scores for erosion (0-250)
- Cartilage is scored 0-4 in 25 locations based on the extent of cartilage destruction (0=normal, 0.5=equivocal thinning, 1.0=mild but definitive thinning, 1.5= <25% thinning, 2.0= 25%-75% thinning, 2.5= >75% loss, 3.0= complete cartilage loss, 3.5= partial ankylosis, 4.0= complete ankylosis) with total score for cartilage (0-100).

<u>DCE-MRI</u>:

- K^{trans} of the total enhancing tissue and total enhancing synovium of the wrist. K^{trans} reflects flow and permeability surface area of enhancing synovium.
- $IAUC_{90}$ the total enhancing tissue or synovium reflects flow and permeability surface area and the contrast agent distribution volume in the first 90 seconds after injection.
- Early Enhancement Rate (EER) (%/sec) and Maximal Enhancement (ME).
- The number of voxels showing contrast uptake, contrast plateau, and contrast washout, alone and in combination using Dynamika RA software.

High Dimensionality Cytometry

This assay may be performed. See **Section 7.6.**

7.7.1.2 Interpharmacodynamic (PD-PD) and Safety-PD Analysis

Pharmacodynamic-pharmacodynamic relationships may be explored.

7.7.1.3 Pharmacogenomics Analysis

The effect of the following genetic polymorphisms on clinical response to infliximab will be investigated:

- v-maf musculoaponeurotic fibrosarcoma oncogene homolog B (MAFB) on chromosome 20 (rs6028945 & rs6071980)
- type I interferon gene (IFNk) on chromosome 9; (rs7046653)
- paraoxonase I (PON1) gene on chromosome 7 (rs854555, rs854548, rs854547)
- TNFα gene promoter, known as the 308 G/A variant (TNF-308) (rs1800629)

- The effect of the following genetic polymorphisms on clinical response to infliximab will be investigated

    - Julia 8-gene Signature ([42])
    - Bienkowska 8-gene Signature ([44])
    - Sekiguchi 18-gene Signature ([45])
    - Tanino 300-gene Signature ([46])
    - Lequerré 8-gene Signature ([47])
    - Koczan 8-gene Signature ([48])
    - IFN 5-gene Signature ([49])

Exploratory pharmacogenomics (PGx) studies may also be performed if significant Pharmacokinetic/Pharmacodynamic (PK/PD) relationships are observed or adverse events are identified. Genomic markers of disease may also be investigated. Pharmacogenetic studies will be conducted with Biostatistics design and analysis and compared to PK/PD results or clinical outcomes. Any significant PGx relationships to outcome will require validation in future clinical trials.

7.7.1.4 PD/PGx, PK/PGx and Safety/PGx Analysis

Pharmacogenomics inter-relationships may be explored.

7.7.1.5 Other Assessments

Other assessments include the Health Assessment Questionnaire ,the Patient and Physician's Global Assessment Visual Analog Scale, and the Patient's Assessment of Pain Visual Analog Scale as described in **Section 7.6**.

7.7.2 Safety Monitoring and Assessments

Safety variables to be assessed include: vital signs, ECGs, reporting of adverse events, hematology and blood chemistry.

7.7.2.1 Definition of Terms

7.7.2.1.1 Adverse Event

Per the International Conference on Harmonization (ICH), an adverse event (AE) is defined as any untoward medical occurrence in a patient or clinical investigation subject administered a pharmaceutical product and which does not necessarily have to have a causal relationship with this treatment. An AE can therefore be any unfavorable and unintended sign (including an abnormal laboratory finding, for example), symptom, or disease temporally associated with the use of a medicinal product, whether or not considered related to this medicinal product.

7.7.2.1.2 Serious Adverse Event

A serious Adverse Event (SAE) is any untoward medical occurrence or effect that at any dose:

1. Results in death;
2. Is life-threatening;
3. Requires hospitalization or prolongation of existing inpatients' hospitalization;
4. Results in persistent or significant disability or incapacity; and/or
5. Is a congenital anomaly or birth defect.

Life-threatening in the definition of serious adverse event, refers to an event in which the subject was at risk of death at the time of the event; it does not refer to an event which hypothetically might have caused death if it were more severe.

Medical judgment should be exercised in deciding whether an adverse event/reaction is serious in other situations. Important adverse events/reactions that are not immediately life-threatening, or do not result in death or hospitalization, but may jeopardize the subject or may require intervention to prevent one of the outcomes listed in the definition above, should also be considered serious.

7.7.2.1.3 Closely Monitored Event

A "closely monitored event" is a non-serious adverse event or occurrence that is designated to be of special interest and must be reported to the sponsor as though it were a serious adverse event - as described in **Section 7.7.2.5.1**.

No adverse events are considered closely monitored for this protocol.

7.7.2.1.4 Overdose

An overdose is a significant variation from the recommended/scheduled dosage for a product. In this current trial an overdose of infliximab is any dose higher than the dose specified in **Section 7.4.1.1** of this protocol.

7.7.2.1.5 Product Quality Complaint

A product quality complaint (PQC) is any written, electronic or oral communication that alleges a product defect. A Product Quality Complaint includes suspected product counterfeit, diversion or tampering. A Product Quality Complaint does not include Product Complaints alleging an Adverse Event.

7.7.2.1.6 Planned Hospitalization

A hospitalization planned by the subject prior to signing the ICF is considered a therapeutic intervention and not the result of a new SAE and should be recorded as medical history. If the planned hospitalization or procedure is executed as planned, the record in the subject's medical history is considered complete. However, if the event/condition worsens during the trial, it must be reported as an AE.

7.7.2.1.7 Medication Error

A medication error is any preventable event that may cause or lead to inappropriate medication use, including unintended accidental exposure or subject or patient harm while the medication is in the control of a health care professional, subject or patient, or consumer. Such events may be related to professional practice, clinical trials, health care products, procedures, and systems, including

prescribing, order communication, product labeling, packaging, and nomenclature; compounding, dispensing, distribution, administration, education, monitoring, and use.

7.7.2.1.8 Potential Medication Error

A potential medication error is an individual case safety report of information or complaint about product name, labeling, or packaging similarities that does not involve a subject or patient (eg, If a subject reports that one of the investigational products looks like a different product, the report would be considered a potential medication error).

7.7.2.1.9 Incidents

Product complaints which led to or might have lead to death or serious deterioration of health/serious injury/serious illness for the user or any other person.

7.7.2.2 Monitoring

7.7.2.2.1 Monitoring Adverse Events

Subjects will be monitored for the occurrence of AEs - including SAEs - immediately after the subject has signed the informed consent form.

Subjects will be questioned and/or examined by the investigator or a qualified designee for evidence of AEs. The questioning of subjects with regard to the possible occurrence of adverse events will be generalized such as, "How have you been feeling since the first dose/yesterday/your last visit?" The presence or absence of specific AEs should not be elicited from subjects.

Subjects having AEs will be monitored with relevant clinical assessments and laboratory tests, as determined by the investigator.

AEs, actions taken as a result of AEs, and follow-up results must be recorded in the Case Report Forms (Reporting Trial Data to the Sponsor; **Section 9.2**), as well as in the subject's source documentation. Follow-up laboratory results should be filed with the subject's source documentation.

For all AEs that require the subject to be discontinued from the trial and SAEs, relevant clinical assessments and laboratory tests will be repeated as clinically appropriate, until final resolution or stabilization of the event(s).

7.7.2.2.2 Monitoring Laboratory Assessments

The clinical laboratory values will reviewed by the investigator for significance and consideration as an AE.

7.7.2.3 Assessment of Adverse Events

7.7.2.3.1 Assessment of Severity

Where the determination of adverse event severity rests on medical judgment, the determination of severity must be made with the appropriate involvement of a medically-qualified investigator.

The severity of AEs will be graded according to the following definitions:

Mild: awareness of sign, symptom, or event, but easily tolerated;

Moderate: discomfort enough to cause interference with usual activity and may warrant intervention;

Severe: incapacitating with inability to do normal daily living activities, or significantly affects clinical status, and warrants intervention;

7.7.2.3.2 Assessment of Causality

A medically-qualified investigator must assess the relationship of any AE (including SAEs) to the use of the investigational product, as unlikely related, possibly related, or probably related, based on available information, using the guidelines listed below:

Unlikely related: no temporal association, or the cause of the event has been identified, or the drug, biological, or device cannot be implicated based on available information;

Possibly related: temporal association, but other etiologies are likely to be the cause; however, involvement of the drug, biological, or device cannot be excluded based on available information;

Probably related: temporal association, other etiologies are possible, but unlikely based on available information.

7.7.2.3.3 Reference Safety Information (RSI) for the Assessment of Expectedness of Adverse Events

The Reference Safety Information (RSI) for assessing the expectedness of an adverse event for infliximab in this current trial is to be the most recent SmPC.

7.7.2.3.4 Known Potential Toxicities of Study Drug

The following AEs were reported during clinical trials and/or during postmarketing experience with infliximab:

- Infusion-related reactions, including dyspnea, urticaria, hypotension, flushing, and headache, occurring after the onset of the infusion or within the 1-to 2-hour observation period following the end of infusion
- Delayed hypersensitivity reactions (myalgia and/or arthralgia with fever and rash within 14 days of infusion)

- Anaphylactic-like reactions, including laryngeal/pharyngeal edema, severe bronchospasm, seizure
- Infections, including sepsis and TB
- CHF (new onset or worsening)
- Positive anti-nuclear antibody titer/double-stranded DNA – these titers may be associated with symptoms suggestive of lupus-like syndrome such as fever, malaise, arthralgia, myalgia, pleurisy, and mild weight loss.
- Lymphoproliferative disorders/malignancy
- Immunogenicity

Refer to the approved labeling for additional information on AEs related to toxicities observed to date.

7.7.2.3.5 Known Adverse Events Relating to the Underlying Clinical Condition

Some common AEs associated with rheumatoid arthritis include: arthralgia, synovitis, fatigue, morning stiffness, weakness, anemia, infections. Any new occurrence of such an event or exacerbation of a preexisting condition must be reported as an AE.

7.7.2.4 Reporting Safety Observations by the Investigator to the Sponsor

7.7.2.4.1 Expedited Reporting of Safety Observations by the Investigator to the Sponsor

Any occurrence of the following events or outcomes in a subject in the trial must be reported expeditiously by the investigator or qualified designee to the sponsor's Global Pharmacovigilance (GPV representative or designee using the Safety Data Reporting Form 1727 provided by the sponsor/designee **within 1 working day of becoming aware of the event**.

1. SAE (including SAEs associated with overdose, medication errors, pregnancy, exposure during pregnancy or lactation – including the pregnancy of a male subject's female partner who has provided written informed consent to provide information regarding pregnancy);

2. Death;

3. Planned hospitalizations (not previously reported in the medical history);

4. Closely monitored event;

5. Incidents associated with the device;

6. Cancer.

Any occurrence of a product quality complaint by a subject in the trial must be reported expeditiously by the investigator or qualified designee to the sponsor or designee using the Investigational Medicinal Product Quality Complaint Form provided by the sponsor/designee **within 1 working day of becoming aware of the event**.

Any occurrence of the following events or outcomes in a subject in the trial must be reported expeditiously by the investigator or qualified designee to the sponsor or designee using the Safety Data Reporting Form 1727 **within 5 working days of becoming aware of the event**.

1. Pregnancy, exposure during pregnancy or lactation NOT associated with an SAE– including the pregnancy of a male subject's female partner who has provided written informed consent to provide information regarding pregnancy;

2. Medication error;

3. Potential medication error; and

4. Overdose.

If the investigator is unsure about when to report an observation from the lists above, the event or outcome should be reported to the sponsor or designee using the Safety Data Reporting Form 1727 within 1 working day.

Any observation reported to the sponsor or designee via the Safety Data Reporting Form 1727 that is also an AE, is to be recorded in the CRF (**Section 9.2**), as well as in the subject's source documentation along with any actions taken as a result of AE and follow-up results.

If an autopsy is performed, the de-identified autopsy report must be provided to the sponsor within 1 working day of the results being available.

The Safety Data Reporting Form 1727 requires that the investigator assess causality of the event relative to the investigational product administered in the trial (Causality is described in **Section 7.7.2.3.2**).

Investigators are to follow the requirements for reporting to the Sponsor information regarding AEs, SAEs, closely monitored AEs, pregnancy exposure, lactation exposure, overdoses, medication errors (including potential), preplanned hospitalizations, investigational medicinal product quality complaints, and subject deaths.

7.7.2.4.2 Expedited Reporting by the Sponsor to a Regulatory Health Authority

GPV will monitor data for safety. The Sponsor will manage the expedited reporting of relevant safety information to concerned health authorities, competent authorities, and IRBs/IECs in accordance with local laws and regulations.

7.7.2.5 Discontinuation, Treatment Interruption, and Unblinding of Blinded Treatment Due to Safety Observations

7.7.2.5.1 Discontinuation

See **Section 7.3.3** for the criteria by which a subject must be discontinued. Should a subject be discontinued from the trial, complete the visit activities as specified for discontinuation in the Trial Flow Chart in **Section 2.2**.

7.7.2.5.2 Temporary Interruption of Treatment for a Subject

A Subject may not temporarily interrupt and then restart treatment. The investigator is to discontinue a subject as necessary according to the criteria provided in **Section 7.3.3**.

7.8 Criteria for Early Termination of the Trial

The clinical trial may be stopped if the extent (incidence and/or severity) of emerging effects/clinical endpoints is such that the risk/benefit ratio to the trial population as a whole is unacceptable. The clinical trial may also be stopped for administrative reasons.

In addition, further recruitment in the trial or at (a) particular site(s) may be stopped due to insufficient compliance with the protocol, GCP and/or other applicable regulatory requirements, procedure-related problems, or the number of discontinuations for administrative reasons is too high.

8 STATISTICAL ANALYSIS AND REPORTING PLANS

8.1 Data Sets

All-Subjects-Evaluable group

The All-Subjects-Evaluable group will consist of all subjects from the All-Subjects-Treated group for whom at least one pharmacodynamic parameter can be calculated according to the protocol and who did not have any protocol violation interfering with pharmacodynamics.

8.2 Demographic and Other Baseline Characteristics

Demographic variables (eg, sex, race, age, weight, elbow breadth) will be listed and summarized using descriptive statistics for the entire study population.

8.3 Pharmacodynamic Analyses

8.3.1 Pharmacodynamics

METHODS: The primary metric for assessing treatment effect at Weeks 14, 4, and 2 is change from baseline. Currently, K^{trans} is considered a primary metric, provided that technically adequate MRI scans are obtained from the first 6 to 10 patients enrolled in this study. If a patient-specific Arterial Input Function (AIF) cannot be measured, then a population AIF may be substituted for patient-specific AIF.

Further, the order of the MRI endpoints may be changed to accommodate data with technically satisfactory collection or to accommodate more tolerable MRI acquisitions.

Primary endpoint will be compared among the treatment groups via a constrained Longitudinal Data Analysis (cLDA) according to Liang and Zeger ([51]). This model assumes a common mean across treatment groups at baseline and different mean for each treatment at each of the post-baseline time points. In this model, the response vector consists of baseline and the values observed at each post-baseline time point. Time is treated as a categorical variable so that no restriction is imposed on the trajectory of the means over time. The analysis model will also adjust for the center. The treatment mean in terms of the mean change from baseline to a given time point will be estimated and tested from this model.

For the primary endpoint, sensitivity analysis will be performed by including treatment by center interaction in the model to verify if the treatment effect is consistent across sites. Additional covariates, for example disease severity, may be included in the model for exploratory purposes. Normality and homogeneity of error variance assumptions will be assessed and appropriate data transformation applied if departures from these assumptions are observed.

Statistical significance of the dichotomous endpoints (ACRx) will be determined using Fisher exact test comparing placebo and treatment groups.

P-values and 90% CI's will be computed. These analyses will be carried out for change from baseline at each of weeks 14 and 6. Interpretation of p-value testing for each endpoint will be made in step-down fashion (using closed stepwise procedure) with week 14 interpreted first. Only if significant treatment difference is observed for week 14, then treatment difference testing of week 4 and then week 2 will be examined. Significance test will be carried out at alpha=0.05, 1-sided for the primary and other study endpoints.

Effect sizes and associated 90% CI's will be computed to assess precision of each endpoint.

COMPOSITE ENDPOINTS: Two composite measures of RA disease activity that incorporate DAS28(CRP) with K^{trans} or RAMRIS synovitis and osteitis will be used to improve discrimination between active treatment from placebo. Precision of the composites will be measured as effect size (infliximab minus placebo divided by pooled SD). Prior to analysis for composites, the individual endpoints (components of the composite) will be standardized using z-scores. The methods used for building composites include O'Brien's global statistics and the first principal component computed from individual endpoints after z-score standardization. For example, O'Brien's global statistics is the average of standardized z-scores across the measures.

GENE SIGNATURES – RESPONDER ANALYSIS:

For this analysis, the responder (good response) and non-responder (moderate or bad response) status for each patient will be inferred by DAS28-ESR EULAR criteria on the change in disease activity measured by DAS28-ESR EULAR response at 14 weeks.

Definition of response: response is defined as both: (1) change in disease activity from baseline and (2) the level of disease activity attained during follow-up. In particular, EULAR response states are classified as follows: good responders are patients with an improvement of >1.2 and a present score of 3.2; moderate responders are patients with an improvement of >0.6 to 1.2 and a present score of 5.1, or an improvement of >1.2 and a present score of >3.2; non-responders are any patients with an improvement of 0.6, or patients with an improvement of >0.6 to 1.2 and a present score of >5.1 ([52]). DAS28-defined remission is classified as a score of <2.6.

Then for each gene signature, a prediction rule will developed with the baseline expression values of the multiple genes in the signature as explanatory variables using a multivariate classifier. Random forest (R package developed by Biometrics Research, Merck) or logistic regression will be used for this purpose. The sensitivity and specificity will be evaluated on the prediction rule. Receiver operating characteristic (ROC) curves will be drawn. The performance of the gene signature will be quantified by the area under the ROC curve. The classifiers will be validated

using external cross-validation approach to correct for the over-optimism of an internal validation used for model building ([53]).

In addition, the gene signatures will be tested for their mean difference of expression values between responder and non-responder groups using a combination of a fixed sequence step-down procedure and Holm's step-down procedure to control the overall alpha level at 0.05. Among the seven gene signatures, the Julia 8-gene Signature will be tested first at alpha=0.05, two-sided. If the test result is non-significant, we will stop and the remaining six signatures remain non-significant for the purpose of conclusions. If the "Julia" signature is shown to be significant, then the other six signatures will be tested by Holm's step-down procedure to control the overall alpha=0.05. More specifically, the p-values from the six signatures will be ordered from the smallest to the largest, $p(1) \le p(2) \le p(3) \le p(4) \le p(5) \le p(6)$, where p(i) is the ith largest p-value for $1 \le i \le k$. Here k=6. If p(1) < alpha / k= 0.0083, the corresponding signature is significant. We will move on to compare p(2) with alpha / (k-1)= 0.01. If the corresponding signature is significant, we will continue to compare p(3) with alpha / (k-2)= 0.0125. If the corresponding signature is significant, we will continue to compare p(4) with alpha / (k-3)= 0.016. If the test result is significant, we will compare p(5) with alpha / (k-4) = 0.025. And if again that is significant, we will test the last signature with the largest p-value, i.e. to compare p(6) with alpha=0.05. If p(6) < 0.05, we can make a conclusion that all signatures are statistically significant. In this Holm's procedure, whenever a non-significant test occurs, the procedure stops for conclusion purposes, and the remaining tests remain non-significant for the purposes of conclusions. For example, if the first signature yields p(1) < 0.0083, the second with p(2) > 0.01, and the third p(3) < 0.0125, the fourth p(4) < 0.016, the fifth p(5) < 0.025, and the sixth p(6) < 0.05, then for the purposes of conclusions, a difference in the expression of the gene signature between responder/non-responder groups can only be concluded at overall alpha=0.05 for the first signature. The p(3) < 0.0125, p(4) < 0.016, p(5) < 0.025 and p(6) < 0.05 difference for the rest of the signatures (3rd to 6th) would be hypothesis generating and not significant at the overall alpha=0.05 level.

In addition to the classification models for the prediction of classes of responder non-responders obtained by dichotomization of the clinical scores, regression model based on random forest will be also developed to predict DAS28(CRP) scores on the continuous scale. The model will be validated using external (nested) cross-validation. Cross-validated (out-of-bag) MSE and R^2 and variable importance for each gene in a particular signature will be reported for this analysis.

<u>Genetic Polymorphisms (Single Nucleotid Polymorphisms SNPs) –Responder Analysis</u>

First, the responder (good response) and non-responder (moderate or bad response) status for each patient will be inferred by DAS28-ESR EULAR criteria on the change in disease activity measured by DAS28-ESR EULAR response at 14 weeks as carried out for the gene signatures before.

Then a logistic regression model on the responder status will be fitted for each of the SNP with term SNP genotype and any other relevant covariates. An additive inheritance model will be used as the primary genetic model. Dominant, recessive and general (categorical) model will also be considered for sensitivity analysis. For each candidate SNP, the odds ratio of the risk allele being 1 in responder vs. non-responder group will be tested. For each significant SNP, a prediction rule will be developed using the univariate analysis. Multivariate classifier using the SNP signature with more than 1 SNP will also be fitted with the SNPs as the independent variables. The sensitivity and specificity will be evaluated on those prediction rules. Receiver operating characteristic (ROC) curves will be drawn for those genetic polymorphisms. The performance of the SNP signature will be quantified by the area under the ROC curve. Similarly to the analysis of gene signatures, external cross-validation will be applied to correct for the over-optimism as an internal validation.

There are four genes (seven SNPs) to be tested for its odds ratio of risk allele in responder versus non-responder groups. Multiplicity will be adjusted by Holm correction to control the overall type I error rate at level 0.05.

In addition to the classification models for the prediction of classes of responders vs non-responders, obtained by dichotomization of clinical scores, a regression model based on random forest will be also developed to predict DAS28(CRP) scores on the continuous scale. The model will be validated using external (nested) cross-validation. Cross-valided (out-of-bag) MSE and R^2 and variable importance for each SNP will be reported for this analysis.

8.3.2 Pharmacodynamic-Pharmacodynamic Analysis

Exploratory pharmacodynamic/pharmacodynamic relationships and analyses will be conducted if deemed appropriate.

8.4 Determination of Sample Size/Power/Level of Significance

Reasonable estimates for treatment effects for infliximab treatment are derived from more recently approved TNF inhibitors or other potent biological agents in active RA patients with inadequate response to DMARDs. A human anti-IL-17 antibody was used in patients with active RA despite DMARDs. About 40% of the population was utilizing methotrexate. Key inclusion criteria were ≥ 6 tender and ≥ 6 swollen joints (28-joint assessment) as well as either C-reactive protein ≥ 1.5 mg/dl or ESR ≥ 28 mm/hour. The mean DAS28 for the 2 mg/kg dose declined from 5.8 ± 0.86 to 3.4 at week 10 with an effect size of approximately 0.54 ([54]). Certolizumab pegol is a TNF inhibitor. In the RAPID1 trial certolizumab pegol was dosed 400 mg at week 0, 2 and 4 then 200 mg every 2 weeks in methotrexate inadequate responders. Key inclusion criteria were ≥ 9 tender and ≥ 9 swollen as well as CRP ≥ 1.5 mg/dl or ESR ≥ 30 mm/hr. Baseline DAS28(ESR) was about 7.0. The change from baseline was 3.3 ± 1.3 in the 200-mg certolizumab group, as compared with 2.4 ±1.3 in the placebo group for an ES of approximately 0.7 at 52 weeks. DAS28 results were not available at week 12, but the clinical benefit, based on ACR responses appears very durable from week 12 though week 52 ([55]). In the GO-FORWARD trial, golimumab had an effect size of from 0.5 to 0.6 on ACR or DAS28 remission endpoints in patients with inadequate response to MTX. These studies

were enrolled at a large number of clinical sites, where it is difficult to standardize clinical assessment. We believe in a smaller study we should be able to standardize examination practices to achieve slightly higher treatment effect sizes. Using these references as a guide we choose to power this study based on an estimate treatment effect size of 0.7 to 0.8.

POWER: It is assumed that infliximab effect sizes for K^{trans} will be at least as large as those for DAS28. The sample size of 26 patients per group has 80% power to yield a statistically significant (alpha=0.05, 1-tailed) difference between treatments if the true underlying effect size is 0.7.

8.5 Interim Analysis

No formal interim analysis is planned.

8.6 Safety

8.6.1 Adverse Events

All AEs noted during the study will be listed. The number of subjects reporting each AE (by treatment group) and severity will also be presented. Treatment-emergent and treatment-related AEs will be tabulated by body system/organ class.

8.6.2 Clinical Laboratory Tests

The results of hematology and blood chemistry will be listed for each subject. Laboratory values outside the normal ranges will be flagged.

8.6.3 Vital Signs

Systolic and diastolic blood pressures, heart rate, and body temperature will be listed for each subject.

8.6.4 Physical Examination

The results of the physical examinations at Screening will be listed in the medical history listing. Post-baseline findings of the physical examinations that meet the criteria of an AE (**Section 7.7.2.2**) will be listed in the relevant AE listings.

8.6.5 Electrocardiogram

The results of the ECG will be listed for each subject. If applicable, summary statistics of ECG results will be provided.

8.6.6 Other Safety

Not applicable.

8.7 Other Analyses

Data collected from the Health Assessment Questionnaire and the Physician's Global Assessment Visual Analog Scale will be reviewed.

9 ADHERENCE TO ETHICAL, REGULATORY, AND ADMINISTRATIVE CONSIDERATIONS

The study must be conducted in accordance with Good Clinical Practice (GCP) as outlined in the International Conference on Harmonisation of Technical Requirements for Registration of Pharmaceuticals for Human Use (ICH) Guidelines, E6 Good Clinical Practice: Consolidated Guidance and other applicable laws and regulations. In addition, the study must be conducted in accordance with: (i) the USA Code of Federal Regulations (CFR) if the study is conducted under a USA IND, regardless of the country involved; (ii) the European Union (EU) Clinical Trial Directive (CTD) and local regulations if the study is conducted in the EU; and (iii) any specific local regulations if the study is conducted elsewhere.

9.1 Ethical Conduct of the Study

9.1.1 Independent Ethics Committee or Institutional Review Board

Prior to initiation of the trial at any site, the trial, including the protocol, informed consent, and other trial documents must be approved by an appropriate Institutional Review Board (IRB) or Independent Ethics Committee (IEC). The IRB/IEC must be constituted according to applicable regulatory requirements. As appropriate, amendments to the protocol must also be approved by the IRBs/IECs before implementation at the sites, unless warranted to eliminate an immediate hazard. The IRB/IEC approval should be obtained in writing, clearly identifying the trial, the documents reviewed (including informed consent), and the date of the review. The trial as described in the protocol (or amendment), informed consent, and other trial documentation may be implemented only after all the necessary approvals have been obtained and the sponsor has confirmed that it is acceptable for the investigator to do so.

In the event that the IRB/IEC requires changes in the protocol, the sponsor shall be advised and must approve the changes prior to implementation. The investigator shall not modify the trial described in the protocol once finalized and after approval by the IRB/IEC without the prior written approval of sponsor.

In countries where the investigator submits the trial protocol and statement of informed consent to the IRB/IEC, the investigator or qualified designee will forward the approvals to the sponsor.

9.1.2 Subject Information and Consent

The details of the protocol must be provided in written format and discussed with each potential subject, and written informed consent must be obtained for all subjects before any trial-related procedure is performed. In obtaining informed consent, the information must be provided in language and terms understandable to the subject. The subject, or the subject's legal representative, must give their written consent to participate in the trial. The signed and dated consent form itself must be retained by the investigator as part of the trial records. A copy of the signed and dated consent form must be given to the subject. The consent form must include all of the required elements of informed consent in accordance with ICH Guidelines E6 and local laws. In addition, the sponsor specifically requests that the consent form identify it as the sponsor and state that use of the investigational product(s) is experimental and the side effects of the investigational product(s) are not completely known. The consent form must be approved by the appropriate IRB/IEC and sponsor before trial initiation at a trial site. Any subsequent changes to the approved informed consent form must be reviewed and approved by the appropriate IRB/IEC and sponsor before implementation.

9.1.3 Subject Identification Card

A Subject Identification Card will be provided to each subject to carry on his or her person (eg, in a wallet) at all times while the subject is participating in the trial, if required in section 2.2. The card is to be shown to caregivers in the event of an emergency.

At a minimum, the card must contain the following information:

1. Protocol number;
2. The subject's protocol identification number;
3. A statement identifying the card-carrier as a participant in a clinical trial (eg, "This person is participating in a clinical research trial.");
4. A statement indicating the person might be taking an investigational drug (eg, "This person is taking an experimental drug which could have interactions with other medications, or placebo"); and
5. Contact information in the event of an emergency or hospitalization. The contact information on the card is to be the investigator or a designated site contact, rather an contact from within the sponsor;

The cards may also include other trial-specific information to assist with treatment decisions in the event of an emergency, such as types of concomitant therapies that may, or may not be, permitted as part of emergency treatment. As with any other information provided to subjects, the Subject Identification Card must be approved by the IRB/IEC. Monitors will request that Investigators provide Subject Identification Cards to each subject. Investigators will be asked to request that subjects carry the cards with them while they are participating in the trial.

The Investigator/site should collect the cards at the end of the trial and retain them with other clinical trial documents.

9.1.4 Registration of the Trial

The trial will be registered by the sponsor on appropriate free public web sites such as www.clinicaltrials.gov, which is a service of the United States National Institutes of Health, and EudraCT, https://eudract.ema.europa.eu/.

9.2 Reporting Trial Data to the Sponsor

9.2.1 Data Collection Forms

The Sponsor will provide the site with data collection forms, be they Case Report Forms (CRF), either in paper format or electronic Case Report Forms (eCRF); diaries; Electronic Data Capture (EDC) screens; or other appropriate data collection forms as the trial requires. The investigator is to provide subject data according to the Sponsor's instructions, in the designated data collection form, compliant with GCP practices. The Sponsor will also provide the site with instructions for assisting other parties - such as a central laboratory - to collect data. As instructed by the Sponsor, a designated central laboratory may collect data in a database and provide the completed database to sponsor. All data collection forms and the databases from the trial are the exclusive property of sponsor.

The investigator must maintain records and data during the trial in compliance with all applicable legal and regulatory requirements. Each data point must be supported by a source document at the trial site. Any records or documents used as the source of information (called the "subject source data") are to be retained for review by authorized representatives of the sponsor or a regulatory agency. For example, if CRF pages or EDC screens will be used as source documents for specific information, then a copy of the completed CRF pages or EDC screen should be retained by the site.

The investigator will ensure that there are sufficient time, staff, and facilities available for the duration of the trial to conduct and record the trial as described in the protocol and according to all applicable guidances, laws, and regulations.

All data collection forms such as CRFs, diaries; EDC screens; electronic database entries, should be completed as soon as possible after the evaluation has occurred. All dates appearing on the sponsor's subject data collection forms for laboratory tests, cultures, and other data collected, must be the dates on which the specimens were obtained, or the procedures performed.

9.2.2 Preparing Case Report Forms for All Subjects

The Sponsor must not collect subject names, initials, or other personal information that is beyond the scope of the trial from any subject. Subjects are not to be identified by name or initials on the CRF or any trial documents. The only acceptable identification for a subject that may appear on a CRF or trial document is the unique subject identification number. The investigator must maintain contact information for each participant so that all can be quickly contacted by the investigator, if necessary.

All entries into CRFs are the responsibility of the investigator and must be completed by the investigator or a qualified designee. The investigator will attest in writing at the beginning of the trial that his/her electronic signature is the legally binding equivalent of a written signature and will acknowledge by entering his/her electronic signature that he/she has verified the accuracy of the recorded data.

9.2.3 Preparing Case Report Forms for Subjects Who Fail Screening

Data are to be collected from the time the informed consent form is signed until the subject is determined to have failed screening. A CRF with a minimum of the following information must be completed for subjects who fail screening:

(1) demographics, (2) subject status, (3) reason for screen failure, and (4) serious adverse events.

9.3 Publications and Other Rights

9.3.1 Rights to Publish by the Investigator

The investigator has the right to publish or publicly present the results of the trial in accordance with this **Section 9.3** of the protocol. In the event that the protocol is a part of a multi-site trial, it is understood that it is the intent of the sponsor and the investigator to initially only publish or present the trial results together with the other sites, unless specific written permission is obtained in advance from the sponsor to publish separate results. The sponsor shall advise as to the implications of timing of any publication in the event clinical trials are still in progress at sites other than the investigator's site.

The investigator agrees not to publish or publicly present any interim results of the trial without the prior written consent of the sponsor. The investigator further agrees to provide to the sponsor 45 days prior to submission for publication or presentation, review copies of abstracts or manuscripts for publication (including, without limitation, slides and texts of oral or other public presentations and texts of any transmission through any electronic media, eg, any computer access system such as the Internet, World Wide Web, etc) that report any results of the trial. The sponsor shall have the right to review and comment with respect to publications, abstracts, slides, and manuscripts and the right to review and comment on the data analysis and presentation with regard to the following concerns:

1. proprietary information that is protected by the provisions contained in **Section 9.3.2**;
2. the accuracy of the information contained in the publication; and
3. to ensure that the presentation is fairly balanced and in compliance with FDA regulations.

If the parties disagree concerning the appropriateness of the data analysis and presentation, and/or confidentiality of the sponsor's confidential information, investigator agrees to meet with the sponsor's representatives at the clinical trial site or as otherwise agreed, prior to submission for publication, for the purpose of making good faith efforts to discuss and resolve any such issues or disagreement.

9.3.2 Use of Proprietary or Confidential Information in a Publication

No publication or manuscript shall contain any trade secret information of the sponsor or any proprietary or confidential information of the sponsor and shall be confined to new discoveries and interpretations of scientific fact. If the sponsor believes there is patentable subject matter contained in any publication or manuscript submitted for review, the sponsor shall promptly identify such subject matter to investigator. If sponsor requests and at sponsor's expense, investigator shall use its best efforts to assist sponsor to file a patent application covering such subject matter with the USA Patent and Trademark Office or through the Patent Cooperation Treaty prior to any publication.

9.3.3 Use of Trial Information in a Publication

Investigator is granted the right subject to the provisions of this protocol to use the results of all work provided by investigator under this protocol, including but not limited to, the results of tests and any raw data and statistical data generated for

investigator's own teaching, research, and publication purposes only. Investigator/Institution agrees, on behalf of itself and its employees, officers, trustees, and agents, not to cause said results to be knowingly used for any commercial purpose whatsoever except as authorized by the sponsor in writing.

9.3.4 Authorship of Publications

Authors of publications must meet the International Committee of Medical Journal Editors (ICMJE) guidelines for authorship and must satisfy the 3 criteria that follow:

1. Authors must make substantial contributions to the conception and design of the trial, acquisition of data, or analysis of data and interpretation of results;

2. Authors must draft the publication or, during draft review, provide contributions (data analysis, interpretation, or other important intellectual content) leading to significant revision of the manuscript with agreement by the other authors;

3. Authors must provide written approval of the final draft version of the publication prior to submission.

All contributors who do not meet the 3 criteria for authorship should be listed in an acknowledgments section within the publication, if allowed by the journal, per the ICMJE guidelines for acknowledgment.

9.4 Trial Documents and Records Retention

During the trial and after termination of the trial – including after early termination of the trial – the investigator must maintain copies of all documents and records relating to the conduct of the trial. This documentation includes, but is not limited to, protocols, CRFs and other data collection forms, advertising for subject participation, adverse event reports, subject source data, correspondence with health authorities and IRBs/IECs, consent forms, investigator's curricula

vitae/biosketch, monitor visit logs, laboratory reference ranges, and laboratory certification or quality control procedures and laboratory director curriculum vitae. Subject files and other source data must be kept for the maximum period of time permitted by the hospital, institution or private practice, or as specified below. The sponsor must be consulted if the investigator wishes to assign the files to someone else, remove them to another location, or is unable to retain them for the specified period.

The investigator must retain trial records for the amount of time specified by applicable laws and regulations. At a minimum, trial records must be retained for the amount of time specified by ICH Guidelines or the EU Good Clinical Practices Directive, or applicable local laws, whichever is longer:

1. The ICH Guidelines specify that records must be retained for a minimum of 2 years after a marketing application for the indication is approved (or not approved) or 2 years after notifying the appropriate regulatory agency that an investigation is discontinued.

2. The European Union (EU) Commission Directive 2003/63/EC which requires that Essential Documents (including Case Report Forms) other than subjects' medical files, are retained for at least fifteen (15) years after completion or discontinuation of the trial, as defined in the protocol.

   All trial documents shall be made available if required by relevant health authorities. The investigator should consult with the sponsor prior to discarding trial and/or subject files.

Sponsor will retain all sponsor-required documentation pertaining to the trial for the lifetime of the investigational product. Archived data may be held on microfiche or electronic record, provided that a back-up exists and that a paper copy can be obtained from it, if required.

10 INVESTIGATORS AND TRIAL ADMINISTRATIVE STRUCTURE

10.1 Sponsor

The sponsor of this trial is indicated in **Section 1**, Title Page.

10.2 Investigators

10.2.1 Selecting Investigators

Only investigators qualified by training and experience to perform this clinical trial are selected. The sponsor will contact and select all investigators (ie, the legally responsible party[ies] at each trial site), who, in turn, will select their staff.

10.2.2 Financial Disclosure Requirement

In connection with the clinical trial described in the protocol, the investigator certifies that, if asked, the investigator will read and answer the Certification/Disclosure Form or equivalent document truthfully and to the best of investigator's ability. Investigator also certifies that, if asked, the investigator will have any other applicable party(ies) (eg, subinvestigators) read and answer the Certification/Disclosure Form as a condition of their participation in the trial.

If the financial interests reported on the Certification/Disclosure Form change during the course of the trial or within 1 year after the last subject has completed the trial as specified in the protocol, the investigator and the other applicable party(ies) are obligated to inform the sponsor of such financial change.

10.2.3 Clinical Study Report Coordinator Investigator

A Clinical Study Report (CSR) will be prepared by the sponsor or its qualified designee to describe the results of the trial. One of the investigators shall be selected by the sponsor to review the CSR and provide approval of the final CSR in writing. The investigator chosen to review and approve the CSR is to be called the CSR Coordinating Investigator. A second investigator shall be selected as the Alternate CSR Coordinating Investigator. The Alternate CSR Coordinating Investigator is to review and approve the CSR should the first CSR Coordinating Investigator be unable to do so. The sponsor is to select the CSR Coordinating Investigator and Alternate CSR Coordinating Investigator from the investigators using the following criteria:

1. Must be the Principal Investigator at a trial site actively enrolling subjects and participating in the trial;
2. Must be willing and capable of completing the necessary reviews and providing approval of the CSR in writing.

10.3 Central Organizations

Central organizations may be contracted to perform site qualification, study monitoring, imaging data analyses, and other study related tasks. A central laboratory will be used for the measurement of CRP.

11 REFERENCES

1. Remicade® Summary of Product Characteristics

2. Kremer JM, Bloom BJ, Breedveld FC, Coombs JH, Fletcher MP, Gruben D, et al. The Safety and Efficacy of a JAK Inhibitor in Patients With Active Rheumatoid Arthritis: Results of a Double-Blind, Placebo-Controlled Phase IIa Trial of Three Dosage Levels of CP-690,550 Versus Placebo. Arthritis Rheum 2009; 60(7):1895–1905.

3. Genovese MC, Becker JC, Schiff M, Luggen M, Sherrer Y, Kremer J, et al. Abatacept for rheumatoid arthritis refractory to tumor necrosis factor alpha inhibition. N Engl J Med. 2005 Sep 15;353(11):1114-23. Erratum in: N Engl J Med. 2005 Nov 24;353(21):2311.

4. Furst DE, Fleischmann R, Kopp E, Schiff M, Edwards C 3rd, Solinger A, Macri M; 990136 Study Group. A phase 2 dose-finding study of PEGylated recombinant methionyl human soluble tumor necrosis factor type I in patients with rheumatoid arthritis. J Rheumatol. 2005 Dec;32(12):2303-10.

5. Kalden-Nemeth D, Grebmeier J, Antoni C, Manger B,Wolf F, Kalden JR. NMR monitoring of rheumatoid arthritis patients receiving anti-TNF- α monoclonal antibody therapy. Rheumatol Int 1997; 16:249-255.

6. Brown AK, Conaghan PG, Karim Z, Quinn MA, Ikeda K, Peterfy CG, Hensor E, Wakefield RJ, O'Connor PJ, Emery P. An explanation for the apparent dissociation between clinical remission and continued structural deterioration in rheumatoid arthritis. Arthritis Rheum. 2008 Oct;58(10):2958-67.

7. Uhlig T, and Kvien TK. Is rheumatoid arthritis really getting less severe? Nature Reviews Rheumatology 5, 461-464 (August 2009).

8. Hetland ML, Ejbjerg B, Hørslev-Petersen K, et al. MRI bone oedema is the strongest predictor of subsequent radiographic progression in early rheumatoid arthritis: Results from a 2-year randomised controlled trial (CIMESTRA). Ann Rheum Dis 2009; 68: 384-390.

9. Conaghan P, O'Connor P, McGonagle D, Astin P, Wakefield R, Gibbon W, et al. Elucidation of the Relationship Between Synovitis and Bone Damage: A Randomized Magnetic Resonance Imaging Study of Individual Joints in Patients With Early Rheumatoid Arthritis. Arthritis Rheum 2003; 48(1):64-71.

10. Greenwood MC , Rathi J, Hakim AJ, Scott DL, Doyle DV. Regression to the mean using the disease activity score in eligibility and response criteria for prescribing TNF-α inhibitors in adults with rheumatoid arthritis. Rheumatology 2007;46:1165–1167.

11. Maini R., et al. Infliximab versus placebo in rheumatoid arthritis patients receiving concomitant methotrexate: a randomized phase III trial. Lancet 1999; 354: 1932–39.

12. Quinn MA et al. Very early treatment with infliximab in addition to methotrexate in early, poor-prognosis rheumatoid arthritis reduces magnetic resonance imaging evidence of synovitis and damage, with sustained benefit after infliximab withdrawal: results from a twelve-month randomized, double blind, placebo-controlled trial. Arthritis Rheum 2005;52:27–35.

13. Østergaard M, Peterfy C, Conaghan P, McQueen F, Bird P, Ejbjerg B, et al. OMERACT Rheumatoid Arthritis Magnetic Resonance Imaging Studies: Core Set of MRI Acquisitions, Joint Pathology Definitions, and the OMERACT RA-MRI Scoring System. J Rheumatol 2003; 30:1385-1386.

14. McQueen F, Lassere M, Edmonds J, Conaghan P, Peterfy C, Bird P, et al. OMERACT Rheumatoid Arthritis Magnetic Resonance Imaging Studies: Summary of OMERACT 6 MR Imaging Module. J Rheumatol 2003;30:1387-92.

15. Haavardsholm EA, Østergaard M, Ejbjerg BJ, Kvan NP, Uhlig TA, Lilleas FG, Kvien TK. Reliability and Sensitivity to Change of the OMERACT Rheumatoid Arthritis Magnetic Resonance Imaging Score in a Multireader, Longitudinal Setting. Arthritis Rheum 2005; 52(12):3860-3867.

16. Konig H, Sieper J, Wolf KJ. Rheumatoid arthritis: evaluation of hypervascular and fibrous pannus with dynamic magnetic resonance imaging enhanced with Gd DTPA. Radiology 1990;176:473-7.

17. Gaffney K, Cookson J, Blake D, Coumbe A, Blades S. Quantification of rheumatoid synovitis by magnetic resonance imaging. Arthritis Rheum 1995;38:1610-7.

18. Østergaard M. Different approaches to synovial membrane volume determination by magnetic resonance imaging: Manual versus Automated Segmentation. British Journal of Rheumatology 1997;36:1166-1177.

19. Ostendorf B, Peters R, Dann P, et al. Magnetic resonance imaging and miniarthroscopy of metacarpophalangeal joints. Arthritis Rheum 2001;44:2492-502.

20. McQueen FM, Gao A, Ostergaard M, King A, Shalley G, Robinson E, Doyle A, Clark B, Dalbeth N High-grade MRI bone oedema is common within the surgical field in rheumatoid arthritis patients undergoing joint replacement and is associated with osteitis in subchondral bone. Ann Rheum Dis. 2007 Dec;66(12):1581-7.

21. Østergaard M, et al.. Magnetic resonance imaging for accelerated assessment of drug effect and prediction of subsequent radiographic progression in rheumatoid arthritis: a study of patients receiving combined anakinra and methotrexate treatment. Ann Rheum Dis. 2005 Oct;64(10):1503-6.

22. Durez P, Malghem J, Toukap AN, Depresseux G, Lauwerys BR, Westhovens R, et al. Treatment of Early Rheumatoid Arthritis: A Randomized Magnetic Resonance Imaging Study Comparing the Effects of Methotrexate Alone, Methotrexate in Combination With Infliximab, and Methotrexate in Combination With Intravenous Pulse Methylprednisolone. Arthritis Rheum 2007 Dec;56(12):3919-3927.

23. Haavardsholm EA, Østergaard M, Hammer HB, Bøyesen P, Boonen A, van der Heijde D, Kvien TK. Monitoring anti-TNFa treatment in rheumatoid arthritis: responsiveness of magnetic resonance imaging and ultrasonography of the dominant wrist joint compared with conventional measures of disease activity and structural damage. Ann Rheum Dis 2009;68:1572–1579.

24. Taylor PC, Sivakumar B. Hypoxia and angiogenesis in rheumatoid arthritis. Current Opinion in Rheumatology 2005; 17:293-298.

25. Ng CT, Biniecka M, Kennedy A, McCormick J, Fitzgerald O, Bresnihan B, Buggy D, Taylor CT, O'Sullivan J, Fearon U, Veale DJ. Synovial tissue hypoxia and inflammation in vivo. Ann Rheum Dis. 2010 Jul;69(7):1389-95.

26. Hodgson RJ, O'Connor P, Moots R. MRI of rheumatoid arthritis—image quantitation for the assessment of disease activity, progression and response to therapy. Rheumatology 2008;47:13–21 (Review).

27. Tofts PS, Brix G, Buckley DL, et al. Estimating kinetic parameters from dynamic contrast-enhanced T(1)-weighted MRI of a diffusable tracer: standardized quantities and symbols. J Magn Reson Imaging 1999; 10(3):223–232.

28. Hodgson R et al. Dynamic contrast enhanced MRI of bone marrow oedema in rheumatoid arthritis. 2008 Ann Rheum Dis. 2008 Feb;67(2):270-2.

29. Ashton E, Raunig D, Ng C, Kelcz F, McShane T, Evelhoch J. Scan-rescan variability in perfusion assessment of tumors in MRI using both model and data-derived arterial input functions. J Magn Reson Imaging. 2008 Sep;28(3):791-6.

30. Østergaard M, Stoltenberg M, Lovgreen-Nielsen P, Volck B, Sonne-Holm S, Lorenzen I. Quantification of synovistis by MRI: Correlation between dynamic and static gadolinium-enhanced magnetic resonance imaging and microscopic and macroscopic signs of synovial inflammation. Magnetic Resonance Imaging 1998;16(7): 743–754.

31. Tamai K, Yamato M, Yamaguchi T, Ohno W. Dynamic magnetic resonance imaging for the evaluation of synovitis in patients with rheumatoid arthritis. Arthritis Rheum 1994 Aug;37(8):1151-1157.

32. Cimmino MA, Innocenti S, Livrone F, Magnaguagno F, Silvestri E, Garlaschi G. Dynamic Gadolinium-Enhanced Magnetic Resonance Imaging of the Wrist in Patients With Rheumatoid Arthritis Can Discriminate Active From Inactive Disease. Arthritis Rheum 2003 May;48(5):1207–1213.

33. Szkudlarek M, Court-Payen M, Strandberg C, Klarlund M, Klausen T, Østergaard M. Power Doppler Ultrasonography for Assessment of Synovitis in the Metacarpophalangeal Joints of Patients With Rheumatoid Arthritis: A Comparison With Dynamic Magnetic Resonance Imaging. Arthritis Rheum 2001 Sep;44(9): 2018–2023.

34. Østergaard M, Stoltenberg M, Henriksen O, Lorenzen I. Quantitative assessment of synovial inflammation by dynamic gadolinium-enhanced magnetic resonance imaging. A study of the effect of intra-articular methylprednisolone on the rate of early synovial enhancement. Br J Rheumatol 1996;35:50–9.

35. Reece RJ, Kraan MC, Radjenovic A et al. Comparative assessment of leflunomide and methotrexate for the treatment of rheumatoid arthritis, by dynamic enhanced magnetic resonance imaging. Arthritis Rheum 2002;46:366–72

36. Tam LS, Griffith JF, Yu AB, Li TK, Li EK. Rapid improvement in rheumatoid arthritis patients on combination of methotrexate and infliximab: clinical and magnetic resonance imaging evaluation. Clin Rheumatol 2007; 26:941–946.

37. Hodgson R et al. Dynamic contrast enhanced MRI of bone marrow oedema in rheumatoid arthritis. 2008 Ann Rheum Dis. 2008 Feb;67(2):270-2.

38. Fritz, J et al. Longitudinal changes in RA after rituximab administration assessed by quantitative and DCE 3T MR imaging Eur Radiol (2009) 19:2217-2224.

39. Østergaard M, Hansen M, Stoltenberg M, Lorenzen I. Quantitative assessment of the synovial membrance in the rheumatoid wrist: An easily obtained MRI score reflects the synovial volume. Brxitish Journal of Rheumatology 1996;35:965-971.

40. Østergaard M, Hansen M, Stoltenberg M, Gideon P, Klarlund M, Jensen KE, Lorenzen I. Magnetic resonance imaging-determined synovial membrane volume as a marker of disease activity and a predictor of progressive joint destruction in the wrists of patients with rheumatoid arthritis. Arthritis Rheum 1999 May; 42(5):918–929.

41. Kubassova OA, Boyle RD, Radjenovic A. Quantitative Analysis of Dynamic Contrast-Enhanced MRI Datasets of the Metacarpophalangeal Joints. Acad Radiol 2007; 14:1189-1200.

42. Julia, A et al. An eight-gene blood expression profile predicts the response to infliximab in rheumatoid arthritis. PLoS One. 2009 Oct 22;4(10):e7556.

43. LeQuerre, T et al. Management of Infusion Reactions to Infliximab in Patients with Rheumatoid Arthritis or Spondyloarthritis: Experience from an Immunotherapy Unit of Rheumatology. J Rheumatol 2006;33:1307–14

44. Bienkowska JR, Dalgin GS, Batliwalla F, Allaire N, Roubenoff R, Gregersen PK, Carulli JP. Convergent random forest predictor: Methodology for predicting drug response from genome-scale data applied to anti-TNF response. Genomics 2009; 94(6):423-32.

45. Sekiguchi N, Kawauchi S, Furuya T, Inaba N, Matsuda S, Ando S, et al. Messenger ribonucleic acid expression profile in peripheral blood cells from RA patients following treatment with an anti-TNF-_ monoclonal antibody, infliximab Rheumatology 2008;47(6):780-8.

46. Tanino M, Matoba R, Nakamura S, Kameda H, Amano K, Okayama T, et al. Prediction of efficacy of anti-TNF biologic agent, infliximab, for rheumatoid arthritis patients using a comprehensive transcriptome analysis of white blood cells. Biochem Biophys Res Commun 2009; 387(2):261-5.

47. Lequerré T, Gauthier-Jauneau A, Bansard C, Derambure C, Hiron M, Vittecoq O, et al. Gene profiling in white blood cells predicts infliximab responsiveness in rheumatoid arthritis. Arthritis Research & Therapy 2006, 8:R105.

48. Koczan D, Drynda S, Hecker M, Drynda A, Guthke R, Kekow J, Thiesen HJ. Molecular discrimination of responders and nonresponders to anti-TNFalpha therapy in rheumatoid arthritis by etanercept. Arthritis Research & Therapy 2008, 10:R50.

49. Van Baarsen LG, Wijbrandts CA, Rustenburg F, Cantaert T, van der Pouw Kraan TC, Baeten DL, et al. Regulation of IFN response gene activity during infliximab treatment in rheumatoid arthritis is associated with clinical response to treatment. Arthritis Research & Therapy 2010, 12:R11.

50. Kotecha N, Flores NJ, Irish JM, Simonds EF, Sakai DS, Archambeault S, et al. Single-cell profiling identifies aberrant STAT5 activation in myeloid malignancies with specific clinical and biologic correlates. Cancer Cell. 2008 Oct 7;14(4):335-43.

51. Liang KY, Zeger SL. Longitudinal data analysis of continuous and discrete responses for pre-post designs. Sankya: The Indian Journal of Statistics, 62, 134-148, 2000.

52. Van Gestel et al. Development of the European League against rheumatism response criteria for rheumatoid arthritis. Comparison with the preliminary ACR and the WHO/International league against rheumatism criteria. Arthritis Rheum. 1996;39(1): 34-40.

53. Harrell FE. 2001 Regression modeling strategies, Springer, 2001.

54. Genovese MC, Van den Bosch F, Roberson SA, Bojin S, Biagini IM, Ryan P, Sloan-Lancaster J. LY243982, a humanized anti-interleukin-17 monoclonal antibody, in the treatment of patients with rheumatoid arthritis: A phase I randomized, double-blind, placebo-controlled proof-of-concept study. Arthritis Rheum, 2010, 62(4), 929-39.

55. Keystone E, Heide D, Mason D, Landewe R, Vollenhoven RV, Combe B, et al. Certolizumab pegol plus methotrexate is significantly more effective than placebo plus methotrexate in active rheumatoid arhtritis: findigs of a fifty two week, phase III, multicenter, randomized, double-blind, placebo controlled parallel-group study. Arhtritis Rheum, 2008, 58(11):3319-29.

Appendix 1 DNA Sampling and Pharmacogenetic Analysis Procedures

1. Definitions

a. Pharmacogenomics: The investigation of variations of DNA and RNA characteristics as related to drug response.

b. Pharmacogenetics: A subset of pharmacogenomics, pharmacogenetics is the influence of variations in DNA sequence on drug response.

c. Genomic Biomarkers: A measurable DNA and/or RNA characteristic that is an indicator of normal biologic processes, pathogenic processes, and/or response to therapeutic or other interventions.

d. DNA: Deoxyribonucleic acid.

e. RNA: Ribonucleic acid.

2. Summary of Procedures for Pharmacogenomics

a. Subjects for Enrollment: All subjects enrolled in the current clinical trials will be considered for enrollment.

b. Consent

Informed consent for biosamples (ie, DNA, RNA, protein, etc) will be obtained during screening for protocol enrollment from all subjects or legal guardians, at an outpatient visit, or during an inpatient stay by the investigator or his or her designate.

Subjects are not required to participate in the pharmacogenomic sub-study in order to participate in the main trial.

3. Scope of Pharmacogenomic Study

The DNA, RNA, serum, and plasma samples collected in the current trial will be used to study various genetic causes for how subjects may respond to a drug. The DNA, RNA, serum, and plasma, samples will be stored to provide a resource for future studies conducted by Schering-Plough focused on the study of genes responsible for how a drug enters and is removed by the body, how a drug works, other pathways a drug may interact with, or other aspects of disease. All samples will be used by Schering-Plough or designees and research will be monitored and reviewed by a committee of our scientists and clinicians.

4. Techniques to Collect Samples

Blood, plasma, serum samples will generally be obtained for all study participants. Blood samples for both DNA and RNA isolation will usually be obtained at a time when the subject is having blood drawn for other study purposes.

5. Confidential Subject Information for Pharmacogenomic Analysis

Samples will be collected and sent to the laboratory designated for the trial where they will be processed (ie, DNA or RNA extraction, etc) following the Schering-Plough approved policies and procedures for sample handling and preparation.

When samples are collected for a specific genotype or expression analysis, this analysis will be detailed in the main body of the clinical protocol. These samples will be processed, analyzed, and the remainder of the sample will be destroyed. The results of these analyses will be reported along with the other study results. A separate sample will be obtained from subjects in these protocols for storage in the biorepository for future analyses.

To maintain privacy of information collected from samples obtained for storage and future analysis, Schering-Plough has developed secure policies and procedures to maintain subject privacy. At the clinical site, a unique Code will be placed on the blood sample for transfer to the storage facility. The Code is a random number used only to identify the biosample of each subject. No other personal identifiers will appear on the sample tube. The first Code will be replaced with a Sample Code (eg, Genetic Sample Code for DNA sample, Serum Sample code for serum sample) at the Central Laboratory or at the Schering-Plough designated facility. This sample is now a single coded sample. The Sample Code is stored separately from all previous sample identifiers. A secure code, hereinafter referred to as a “first coding key”, will be utilized to match the Sample Code to the original blood code and subject number to allow clinical information collected during the course of the study to be associated with the biosample. This “first coding key” will be transferred by the central laboratory or Schering-Plough designated facility under secure procedures to the Schering-Plough group designated as the entrusted keyholder to maintain confidentiality of the biosamples. The Sample Code will be logged into the primary biorepository database, and in this database this identifier will not have identifying demographic data or identifying clinical information (ie, race, sex, age, diagnosis, lab values) associated with it. The sample will be stored in a designated repository site with secure policies and procedures for sample storage and usage.

For DNA samples, a Storage Code will replace the Sample Code at the Schering-Plough designated facility. The DNA sample is now a double coded sample. This storage code will be stored separately from all previous sample identifiers. The second secure key referred to as a “second coding key” file will be transferred by the Schering-Plough designated facility under secure procedures to the Schering-Plough entrusted keyholder. Samples with the second code are sometimes referred to as de-identified samples. The use of the second code provides additional confidentiality and privacy protection for subjects over the use of a single code. Access to both coding keys is needed to link any data or samples back to a subject identifier.

The “keys” could be utilized to reconstruct the link between genetic information and identifiable clinical information, at the time of analysis. This linkage would not be possible for the investigator conducting the analysis, but may only be done by the Schering-Plough entrusted keyholder under strict security policies and procedures. The Schering-Plough entrusted keyholder will link the information, conduct the analysis, then issue an anonymized data summary on the initially single or double coded samples to the investigator conducting the genomic analysis. The only circumstance by which genomic information would be linked to clinical information would be those situations mandated by health authorities (eg, EMEA, FDA), whereby this information would be directly transferred to the health authority. Once the link between subject’s identifiers and the unique codes is deleted, it is no longer possible to trace the data and samples back to individual subjects through the coding keys. Anonymization is intended to prevent subject re-identification.

6. Biorepository Sample Usage

Samples obtained for the Schering-Plough Biorepository will be used for analyses using good scientific practices. Exploratory analyses will not be conducted under highly validated conditions. The scope of research performed on these samples is limited to the investigation of the variability in inherited biomarkers that may correlate with a clinical phenotype in subjects.

Genomic analysis utilizing the DNA,RNA, plasma, serum samples may be performed by the sponsor, or an additional third party (eg, a university investigator) designated by the sponsor. The investigator conducting the analysis will be provided with a double (single) coded sample. Reassociation of analysis results with corresponding clinical data will only be conducted by the Schering-Plough entrusted keyholder. Any contracted third party genomic analysis will conform to the specific genomic analysis outlined in the clinical protocol. DNA, RNA, plasma, serum sample remaining with the third party vendor after genomic analysis will be returned to the sponsor or destroyed and documentation of destruction will be reported to Schering-Plough.

Consent form signed by the subject will be kept under secure storage for regulatory reasons. Information contained on the consent form alone cannot be traced to any samples, test results, or medical information once the specimens have been rendered de-identified. Laboratory personnel performing the genomic testing will not have access to the informed consent document, nor will they be able to identify subjects from the double (single) coded specimens. Specimens will be identified to the laboratory only by the Sample double (single) code. Subjects who decline to sign the informed consent document for the sub-study will not have the sample collected or stored, nor will they be discontinued from the main study unless the pharmacogenomics sample is specifically required for study enrollment.

A template of each site's informed consent will be stored in the Sponsor's clinical document repository. Each consent will be assessed for appropriate sample permissions. The tracking number on this document will be used to assign sample permissions for each sample in the entrusted keyholder's Sample Database.

7. Withdrawal From the Biorepository and Pharmacogenomic Database

Subjects may withdraw their consent to store the blood, plasma, and serum sample or the DNA or RNA derived from it. Subjects can also request that their sample be destroyed at any time. If samples can be identified in any way (ie, are not anonymized samples), subjects may withdraw consent for banking samples at any time by contacting the investigator responsible for administering their initial informed consent. At that time, subject samples will be removed from the biorepository. Any DNA, RNA, or other biologic samples will be destroyed, destruction will be documented, and sample database information deleted. However, any analyses performed or data obtained from the samples prior to the subject withdrawing consent will not be deleted.

8. Retention of Data and Biosamples

It is anticipated that data generated from processed samples collected during the course this study will be retained for an indefinite period. DNA specimens will be maintained for potential analysis for 20 years from the acquisition. Samples will be destroyed according to Schering-Plough policies and procedures and this destruction will be documented in the repository database.

9. Data Security

Pharmacogenomic and other research databases are accessible only to authorized sponsor and study administrator research personnel and/or designated collaborators and are only stored and accessible as anonymized data. Database user authentication is highly secure, and is accomplished using network security policies and practices based in international standards (eg, ISO17799) to protect against unauthorized access. The Schering Corporation entrusted key holder maintains control over access to all sample data. These data are collected for pharmacogenomic research purposes only as specified in the clinical protocol and will not be used for any other purpose without explicit consent from the research subject.

10. Reporting of Data to Subjects

There is no definitive requirement in either authoritative ethical guidelines or in relevant laws/regulations globally that research results have to be, in all circumstances, returned to study participant. Some guidelines advocate a proactive return of data in certain instances.

No information obtained from exploratory laboratory studies will be reported to the subject or family, and this information will not be entered into the clinical database maintained by Schering-Plough on subjects. Principle reasons not to inform or return results to the subject include: lack of relevance of data, limitations of predictive capability of research data, concerns of misinterpretation of data, absence of good clinical practices standards in exploratory research.

If any exploratory results are definitively associated with clinical significance for subjects while the Schering-Plough clinical trial is still ongoing, investigators will be contacted with information as to how to offer genomic testing (paid for by Schering-Plough) to subjects enrolled and will be advised that genomic counseling should be made available for all who choose to participate.

If any exploratory results are definitively associated with clinical significance after completion of a clinical trial, Schering-Plough will publish the results without revealing specific subject information, inform all sites who participated in the Schering-Plough clinical trial, and post the anonymized results on our website or other accredited website(s) that allow for public access (eg, Disease-societies who have primary interest in the results) in order that physicians and patients may pursue genomic testing if they wish to do so.

11. Gender, Ethnicity, and Minorities

Although many diagnoses differ in terms of frequency by ethnic population and gender, every effort will be made to recruit all subjects diagnosed and treated on Schering-Plough clinical trials for pharmacogenomic sampling. When studies with samples are conducted and subjects identified to serve as controls, every effort will be made to group samples from subjects and controls to represent the ethnic and gender population representative of the disease under current investigation.

12. Risks Versus Benefits of Pharmacogenomic Testing

For pharmacogenomic testing, risks to the subject have been minimized. Risks include those associated with venipuncture to obtain the whole blood sample. This sample will be obtained at the time of routine blood samples drawn for clinical reasons.

Data privacy concerns of the subject have been strictly protected against with Schering-Plough security, policies and procedures. Data privacy risks are largely limited to rare situations involving possible breach of confidentiality. In this highly unlikely situation there is risk that the information, like all medical information, may be misused.

It is necessary for subject-related data (ie, ethnicity, diagnosis, drug therapy and dosage, age, toxicities, etc) to be reassociated to double (single) coded samples at the time of data analysis. These subject data will be kept in a separate, secure Schering-Plough database, and all samples will be stripped of subject identifiers. No information concerning results obtained from genotyping or biomarker studies conducted with samples from the biorepository will be entered into clinical records, nor will it be released to outside persons or agencies, in any way that could be tied to an individual subject.

13. Self-Reported Ethnicity

Subjects who participate in pharmacogenomic study will be asked to provide self-reported ethnicity. Subjects who do not wish to provide this data may still participate in the pharmacogenomic study.

14. Questions

Any questions related to the genomic informed consent, genomic sampling, genomic sample handling, or genomic sample storage should be e-mailed directly to PPD

Appendix 2 Tuberculosis Screening

Tuberculosis Screening

As part of the TB Screening, an intradermal tuberculin skin test (Mantoux) or QuantiFERON-TB Gold test must be performed **within 6 weeks** prior to the first administration of study agent. Multiple puncture tests (Tine and Heaf) are not acceptable methods of testing.

Tuberculin Skin Testing

Administering the Mantoux Tuberculin Skin Test

The Mantoux tuberculin skin test (CDC, 2000) is the standard method of identifying persons infected with Mycobacterium tuberculosis. Multiple puncture tests should not be used to determine whether a person is infected. Multiple puncture tests are not reliable because the amount of tuberculin injected intradermally cannot be precisely controlled. Tuberculin skin testing is both safe and reliable throughout the course of pregnancy. The Mantoux tuberculin test is performed by placing an intradermal injection of 0.1 mL of tuberculin into the inner surface of the forearm. The test must be performed with tuberculin that has at least the same strength as either 5 tuberculin units (TU) of standard purified protein derivative (PPD)-S or 2 TU of PPD-RT 23, Statens Seruminstitut, as recommended by the World Health Organization. PPD strengths of 1 TU or 250 TU are not acceptable (Menzies, 2000). Using a disposable tuberculin syringe with the needle bevel facing upward, the injection should be made just beneath the surface of the skin.

This should produce a discrete, pale elevation of the skin (a wheal) 6 mm to 10 mm in diameter. To prevent needle stick injuries, needles should not be recapped, purposely bent or broken, removed from disposable syringes, or otherwise manipulated by hand. After they are used, disposable needles and syringes should be placed in punctureresistant containers for disposal. Institutional guidelines

regarding universal precautions for infection control (eg, the use of gloves) should be followed. A trained health care worker, preferably the investigator, should read the reaction to the Mantoux test 48 to 72 hours after the injection. Subjects should never be allowed to read their own tuberculin skin test results. If a subject fails to show up for the scheduled reading, a positive reaction may still be measurable up to 1 week after testing. However, if a subject who fails to return within 72 hours has a negative test, tuberculin testing should be repeated.

The area of induration (palpable raised hardened area) around the site of injection is the reaction to tuberculin. For standardization, the diameter of the induration should be measured transversely (perpendicular) to the long axis of the forearm. Erythema (redness) should not be measured. All reactions should be recorded in millimeters, even those classified as negative.

Interpreting the Tuberculin Skin Test Results

In the US and many other countries, the most conservative definition of positivity for the tuberculin skin test is reserved for immunocompromised patients, and this definition is to be applied here, even though the subjects entering this study may or may not be immunocompromised at Baseline. The purpose of using this conservative definition of positivity is to maximize the likelihood of detecting latent TB.

In the US and Canada, an induration of 5 mm or greater in response to the intradermal tuberculin skin test is considered to be a positive result and evidence for either latent or active TB.

In countries outside the United States and Canada, country-specific guidelines **for immunocompromised patients** should be consulted for the interpretation of

tuberculin skin test results. If no local guidelines for immunocompromised patients exist, US guidelines must be followed.

References

Centers for Disease Control and Prevention. Core curriculum on tuberculosis: What the clinician should know (Fourth Edition). Atlanta, GA: Department of Health and Human Services; Centers for Disease Control and Prevention; National Center for HIV, STD, and TB Prevention; Division of Tuberculosis Elimination; 2000:25-86.

Menzies RI. Tuberculin skin testing. In: Reichman LB, Hershfield ES (eds). *Tuberculosis, a comprehensive international approach.* 2nd ed. New York, NY: Marcel Dekker, Inc;

QuantiFERON-TB Gold Testing

Background

The Mantoux tuberculin skin test (CDC, 2000) is the currently accepted, standard method of identifying persons infected with Mycobacterium tuberculosis. However, the tuberculin skin test has less specificity due to cross-reactivity with BCG and environmental mycobacteria, and reduced sensitivity, especially in immunocompromised persons (ATS, 2000, Huebner et al, 1993, Jasmer et al, 2002, Shafer and Edlin, 1996).

Variability in application and interpretation of the tuberculin skin test is also associated with the inaccuracy of the test (Pouchot et al, 1997). False-positive tuberculin skin test reactions lead to inappropriate treatment with long-term antibiotics, and false-negative tuberculin skin test reactions can result in morbidity

and mortality from active TB that could have been prevented. Because of these tuberculin skin test inadequacies, research has focused on new testing methods for detecting TB infection.

The (IFN-γ) based blood QuantiFERON-TB Gold test is one of the new interferon-γ assays for TB Screening (Cellestis, 2004). It utilizes the recently identified M. tuberculosis-specific antigens ESAT-6 and CFP—10 in the standard format, as well as TB7.7(p4) in the In-Tube format, to detect in vitro cell-mediated immune responses in infected individuals. The QuantiFERON-TB Gold assay measures the amount of IFN-γ produced by sensitized T-cells when stimulated with the synthetic M. tuberculosisspecific antigens. In M. tuberculosis infected persons, sensitized T lymphocytes will secrete interferon-γ in response to stimulation with the M. tuberculosis-specific antigens and, thus, the QuantiFERON-TB Gold test should be positive. Because the antigens used in the test are specific to M. tuberculosis and not found in BCG, the test is not confounded by BCG vaccination, unlike the tuberculin skin test. However, there is some cross-reactivity with the 3 Mycobacterium species M. kansasii, M. marinum, and M. szulgai. Thus, a positive test could be the result of infection with one of these 3 species of Mycobacterium, in the absence of M. tuberculosis infection. In a study of the QuantiFERON-TB Gold test in subjects with active TB, sensitivity has been shown to be approximately 89% (Mori et al, 2004). Specificity of the test in healthy BCG vaccinated individuals has been demonstrated to be more than 98%. In contrast, the sensitivity and specificity of tuberculin skin test was noted to be only about 66% and 35% in a study of Japanese patients with active TB and healthy BCG-vaccinated young adults.

However, sensitivity and specificity of the tuberculin skin test depends on the population being studied, and the tuberculin skin test performs best in healthy young adults that have not been BCG-vaccinated. The sensitivity of the tuberculin skin test can decrease significantly in immunocompromised individuals. In asymptomatic HIV-positive persons who developed active TB, the sensitivity of the tuberculin skin test has been reported to be only 40 to 60% (Shafer and Edlin, 1996).

There are yet no published studies of the performance of the QuantiFERON-TB Gold assay in immunosuppressed populations, but studies are ongoing and preliminary data suggest that sensitivity is maintained and better than the tuberculin skin test (personal communication, Cellestis). There has been 1 published study using a similar IFN-γ based blood test for M. tuberculosis infection (T Spot-TB test), which demonstrated a sensitivity of 90% in HIVpositive persons with active TB (Chapman et al, 2002). The ability of the new IFN-γ-based tests to detect latent infection has been more difficult to study due to the lack of a gold standard diagnostic test; however, several TB outbreak studies have demonstrated that the new tests correlated better with the degree of exposure that contacts had to the index TB case than the tuberculin skin test (Brock et al, 2004, Ewer et al, 2003). Although the performance of the new IFN-γ-based blood tests for active or latent M. tuberculosis infection have not been well-validated in the immunosuppressed population, experts believe these new tests will be at least as sensitive, if not more, and definitely more specific than the tuberculin skin test (Barnes, 2004).

Performing the QuantiFERON-TB Gold Test

The QuantiFERON-TB Gold test is available in 2 formats, a standard method and an In-Tube method. The 2 formats differ logistically in the manner in which they are performed. In addition, the In-Tube format contains 1 additional M. tuberculosis-specific antigen, TB7.7(p4), which is thought to increase the specificity of the test. Results of the QuantiFERON-TB Gold test using either format are acceptable for subjects to enter this trial; however, only the In-Tube format will be provided for this study.

Published studies have only used the QuantiFERON-TB Gold test with the standard method; however, the manufacturing company Cellestis has performed some unpublished studies that demonstrate the comparability of the 2 formats (personal

communication, Cellestis). Both formats of the QuantiFERON-TB Gold test are currently approved for commercial use in Europe and certain other countries in which this trial will be conducted. The QuantiFERON TB Gold test with the standard format was recently approved by the FDA for use in the US. The In-Tube format will be submitted to the FDA for review in the next few years. To perform the test using the In-Tube format, blood is drawn through standard venipuncture into supplied tubes that already contain the M. tuberculosis-specific antigens. Approximately 3 tubes will be needed per patient, each requiring 1 mL of blood. One tube contains the M.Tuberculosis-specific antigens, while the remaining tubes contain positive and negative control reagents. Thorough mixing of the blood with the antigens is done by shaking the tubes. The blood is then incubated for 16 to 24 hours at 37°C, after which tubes are centrifuged for approximately 5 to 10 minutes at 1500 to 2200 g. Following centrifugation, plasma may be harvested from each tube and placed in aliquot tubes, or the centrifuged tubes themselves may be shipped to the central laboratory at 2°C to 8°C. The central laboratory will perform an enzyme-linked immunosorbent assay (ELISA) to quantify the amount of IFN-γ present in the plasma using spectrophotometry and computer software analysis.

Interpreting the QuantiFERON-TB Gold Test Results

The central laboratory will analyze and report results for each subject, and sites will be informed of the results prior to randomization. Subjects who have a positive QuantiFERON TB Gold test must receive the same treatment and evaluations for latent TB infection as subjects who have a positive tuberculin skin test to be enrolled in this trial.

References

American Thoracic Society (ATS). Targeted tuberculin testing and treatment of latent tuberculosis infection. *Am J Respir Crit Care Med* 2000;161:S221-S247.

Barnes PF. Diagnosing latent Tuberculosis infection: Turning glitter to gold [editorial]. *Amer J Respir Crit Care Med* 2004;170:5-6.

Brock I, Weldingh K, Lillebaek T, et al. Comparison of tuberculin skin test and new specific blood test in tuberculosis contacts. *Am J Respir Crit Care Med* 2004;170:65-69.

Cellestis. QuantiFERON-TB Gold clinicians guide and QuantiFERON-TB Gold In-Tube Method package insert. Downloaded from www.cellestis.com, September 2004.

Centers for Disease Control and Prevention. Core curriculum on tuberculosis: What the clinician should know (Fourth Edition). Atlanta, GA: Department of Health and Human Services; Centers for Disease Control and Prevention; National Center for HIV, STD, and TB Prevention; Division of Tuberculosis Elimination; 2000:25-86.

Chapman ALN, Munkanta M, Wilkinson KA, et al. Rapid detection of active and latent tuberculosis infection in HIV-positive individuals by enumeration of Mycobacterium tuberculosis-specific T cells. *AIDS* 2002;16:2285-2293.

Ewer K, Deeks J, Alvarez L, et al. Comparison of T-cell-based assay with tuberculin skin test for diagnosis of Mycobacterium tuberculosis infection in a school tuberculosis outbreak. *Lancet* 2003;361:1168-73.

Huebner RE, Schein MF, Bass JB JR. The tuberculin skin test. *Clinical Infectious Diseases* 1993;17(6):968-975.

Jasmer RM, Nahid P, and Hopewell PC. Latent tuberculosis infection. *New Engl J Med* 2002;347(23):1860-1866.

Mori T, Sakatani M, Yamagishi F, et al. Specific detection of tuberculosis infection: An interferon-γ-based assay using new antigens. *Am J Respir Crit Care Med* 2004;170:59- 64.

Pouchot J, Grasland A, Collet C, et al. Reliability of tuberculin skin test measurement. *Ann Intern Med* 1997;126(3):210-214.

Shafer RW and Edlin BR. Tuberculosis in patients infected with Human Immunodeficiency Virus: Perspective on the past decade. *Clinical Infectious Diseases* 1996;22:683-704.

Appendix 3 ACR Classification Criteria for Diagnosis of Rheumatoid Arthritis

ACR Classification Criteria for Diagnosis of Rheumatoid Arthritis

Using history, physical examination, laboratory and radiographic findings, **4 of the following must be present with 1 through 4 present a minimum of 6 weeks**:

- Morning Stiffness ≥1 hour
- Arthritis of 3 or more of the following joints: right or left PIP, MCP, wrist, elbow, knee, ankle, and MTP joints
- Arthritis of wrist, MCP, or PIP joint
- Symmetric involvement of joints
- Rheumatoid nodules over bony prominences, or extensor surfaces, or in juxta-articular regions
- Positive serum rheumatoid factor
- Radiographic changes including erosions or bony decalcifications localized in or adjacent to the involved joints.

Reference: Arnett FC, et al.: Arthritis Rheum 31:315, 1988.

Appendix 4 Health Assessment Questionnaire

Health Assessment Questionnaire

Name: ____ Date: ____________________

Please place an "x" in the box which best describes your abilities OVER THE PAST WEEK:

	WITHOUT ANY DIFFICULTY	WITH SOME DIFFICULTY	WITH MUCH DIFFICULTY	UNABLE TO DO
**DRESSING & GROOMING**				
**Are you able to**:				
Dress yourself, including shoelaces and buttons?	☐	☐	☐	☐
Shampoo your hair?	☐	☐	☐	☐
**ARISING**				
**Are you able to**:				
Stand up from a straight chair?	☐	☐	☐	☐
Get in and out of bed?	☐	☐	☐	☐
**EATING**				
**Are you able to**:				
Cut your own meat?	☐	☐	☐	☐
Lift a full cup or glass to your mouth?	☐	☐	☐	☐
Open a new milk carton?	☐	☐	☐	☐
**WALKING**				
**Are you able to**:				
Walk outdoors on flat ground?	☐	☐	☐	☐
Climb up five steps?	☐	☐	☐	☐

Please check any AIDS OR DEVICES that you usually use for any of the above activities:

- ☐ Devices used for Dressing (button hook, zipper pull, etc.)
- ☐ Built up or special utensils
- ☐ Crutches
- ☐ Cane
- ☐ Wheelchair
- ☐ Special or built up chair
- ☐ Walker

Please check any categories for which you usually need HELP FROM ANOTHER PERSON:

☐ Dressing and grooming ☐ Arising ☐ Eating ☐ Walking

Please place an "x" in the box which best describes your abilities OVER THE PAST WEEK:

	WITHOUT ANY DIFFICULTY	WITH SOME DIFFICULTY	WITH MUCH DIFFICULTY	UNABLE TO DO
**HYGIENE**				
**Are you able to**:				
Wash and dry your body?	☐	☐	☐	☐
Take a tub bath?	☐	☐	☐	☐
Get on and off the toilet?	☐	☐	☐	☐
**REACH**				
**Are you able to**:				
Reach and get down a 5 pound object (such as a bag of sugar) from above your head?	☐	☐	☐	☐
Bend down to pick up clothing from the floor?	☐	☐	☐	☐
**GRIP**				
**Are you able to**:				
Open car doors?	☐	☐	☐	☐
Open previously opened jars?	☐	☐	☐	☐
Turn faucets on and off?	☐	☐	☐	☐
**ACTIVITIES**				
**Are you able to**:				
Run errands and shop?	☐	☐	☐	☐
Get in and out of a car?	☐	☐	☐	☐
Do chores such as vacuuming or yard work?	☐	☐	☐	☐

Please check any AIDS OR DEVICES that you usually use for any of the above activities:

☐ Raised toilet seat ☐ Bathtub bar ☐ Long-handled appliances for reach

☐ Bathtub seat ☐ Long-handled appliances in bathroom ☐ Jar opener (for jars previously opened)

Please check any categories for which you usually need HELP FROM ANOTHER PERSON:

☐ Hygiene ☐ Reach ☐ Gripping and opening things ☐ Errands and chores

Copyright© 1980 James F. Fries, M.D. – All Rights Reserved

Appendix 5 Infliximab Package Leaflet

PACKAGE LEAFLET: INFORMATION FOR THE USER

Remicade 100 mg powder for concentrate for solution for infusion
Infliximab

Read all of this leaflet carefully before you start using this medicine.
- Keep this leaflet. You may need to read it again.
- Your doctor will also give you a Patient Alert Card, which contains important safety information you need to be aware of before and during your treatment with Remicade.
- If you have any further questions, ask your doctor.
- This medicine has been prescribed for you. Do not pass it on to others. It may harm them, even if their symptoms are the same as yours.
- If any of the side effects gets serious, or if you notice any side effects not listed in this leaflet, please tell your doctor.

In this leaflet:
1. What Remicade is and what it is used for
2. Before you are given Remicade
3. How Remicade will be given
4. Possible side effects
5. How to store Remicade
6. Further information

1. WHAT REMICADE IS AND WHAT IT IS USED FOR

Remicade contains the active substance called infliximab. Infliximab is a type of protein of human and mouse origin.

Remicade belongs to a group of medicines called 'TNF blockers'. It is used in adults for the following inflammatory diseases:
- Rheumatoid arthritis
- Psoriatic arthritis
- Ankylosing spondylitis (Bechterew's disease)
- Psoriasis
- Ulcerative colitis

Remicade is also used in adults and children 6 years of age or older for:
- Crohn's disease.

Remicade works by blocking the action of a protein called 'tumour necrosis factor alpha' (TNF_{α}). This protein is involved in inflammatory processes of the body and blocking it can reduce the inflammation in your body.

Rheumatoid arthritis
Rheumatoid arthritis is an inflammatory disease of the joints. If you have active rheumatoid arthritis you will first be given other medicines. If you do not respond well enough to these medicines, you will be given Remicade which you will take in combination with another medicine called methotrexate to:
- Reduce the signs and symptoms of your disease,
- Slow down the damage in your joints,
- Improve your physical function.

Psoriatic arthritis

Psoriatic arthritis is an inflammatory disease of the joints, usually accompanied by psoriasis. If you have active psoriatic arthritis you will first be given other medicines. If you do not respond well enough to these medicines, you will be given Remicade to:

- Reduce the signs and symptoms of your disease,
- Slow down the damage in your joints,
- Improve your physical function.

Ankylosing spondylitis (Bechterew's disease)
Ankylosing spondylitis is an inflammatory disease of the spine. If you have ankylosing spondylitis you will first be given other medicines. If you do not respond well enough to these medicines, you will be given Remicade to:

- Reduce the signs and symptoms of your disease,
- Improve your physical function.

Psoriasis
Psoriasis is an inflammatory disease of the skin. If you have moderate to severe plaque psoriasis, you will first be given other medicines or treatments, such as phototherapy. If you do not respond well enough to these medicines or treatments, you will be given Remicade to reduce the signs and symptoms of your disease.

Ulcerative colitis
Ulcerative colitis is an inflammatory disease of the bowel. If you have ulcerative colitis you will first be given other medicines. If you do not respond well enough to these medicines, you will be given Remicade to treat your disease.

Crohn's disease
Crohn's disease is an inflammatory disease of the bowel. If you have Crohn's disease you will first be given other medicines. If you do not respond well enough to these, you will be given Remicade to:

- Treat serious, active Crohn's disease,
- Reduce the number of abnormal openings (fistulae) between your bowel and your skin that have not been controlled by other medicines or surgery.

2. BEFORE YOU ARE GIVEN REMICADE

You should not be given Remicade if:

- You are allergic (hypersensitive) to infliximab (the active ingredient in Remicade) or to any of the other ingredients in Remicade (listed in section 6)
- You are allergic (hypersensitive) to proteins that come from mice
- You have tuberculosis (TB) or another serious infection such as pneumonia or sepsis
- You have heart failure that is moderate or severe.

Do not have Remicade if any of the above applies to you. If you are not sure, talk to your doctor before you are given Remicade.

Take special care with Remicade

Check with your doctor before you are given Remicade if you have:

<u>Had treatment with Remicade before</u>

- Tell your doctor if you have had treatment with Remicade in the past and are now starting Remicade treatment again.
  If you have had a break in your Remicade treatment of more than 16 weeks, there is a higher risk for allergic reactions when you start the treatment again.

<u>Infections</u>

- Tell your doctor if you have an infection even if it is a very minor one before you are given Remicade
- Tell your doctor if you have lived in or travelled to an area where infections called histoplasmosis, coccidioidomycosis, or blastomycosis are common before you are given Remicade. These infections are caused by specific types of fungi that can affect the lungs or other parts of your body
- You may get infections more easily when you are being treated with Remicade
- These infections may be serious and include tuberculosis, infections caused by viruses, fungi or bacteria, or other opportunistic infections and sepsis that may, in rare cases, be life-threatening.

Tell your doctor straight away if you get signs of infection during treatment with Remicade. Signs include fever, cough, flu-like signs, feeling unwell, red or hot skin, wounds or dental problems. Your doctor may recommend temporary discontinuation of Remicade.

Tuberculosis (TB)

- It is very important that you tell your doctor if you have ever had TB or if you have been in close contact with someone who has had or has TB
- Your doctor will test you to see if you have TB. Cases of TB have been reported in patients treated with Remicade. Your doctor will record these tests on your Patient Alert Card
- If your doctor feels that you are at risk for TB, you may be treated with medicines for TB before you are given Remicade.

Tell your doctor straight away if you get signs of TB during treatment with Remicade. Signs include persistent cough, weight loss, feeling tired, fever, night sweats.

Hepatitis B virus (HBV)

- Tell your doctor if you have hepatitis B before you are given Remicade
- Tell your doctor if you think you might be at risk of contracting HBV
- Treatment with TNF blockers such as Remicade may result in reactivation of hepatitis B virus in patients who carry this virus.

Heart problems

- Tell your doctor if you have any heart problems, such as mild heart failure
- Your doctor will want to closely monitor your heart function.

Tell your doctor straight away if you get new or worsening signs of heart failure during treatment with Remicade. Signs include shortness of breath or swelling of your feet.

Cancer and lymphoma

- Tell your doctor if you have or have ever had lymphoma (a type of blood cancer) or any other cancer before you are given Remicade
- Patients with severe rheumatoid arthritis, who have had the disease for a long time, may be at higher than average risk of developing lymphoma
- Children and adults taking Remicade may have an increased risk of developing lymphoma or another cancer
- Some children and teenage patients who have received TNF-blockers such as Remicade have developed cancers, including unusual types, which sometimes resulted in death
- Some patients with Crohn's disease or ulcerative colitis who have received Remicade have developed a rare type of cancer called Hepatosplenic T-cell Lymphoma. Most of these patients were adolescent or young adult males. This type of cancer has usually resulted in death. All of these patients had also received drugs known as azathioprine or 6-mercaptopurine.

Lung disease or heavy smoking

- Tell your doctor if you have a lung disease called Chronic Obstructive Pulmonary Disease (COPD) or if you are a heavy smoker, before you are given Remicade

- Patients with COPD and patients who are heavy smokers may have a higher risk of developing cancer with Remicade treatment.

Nervous system disease
- Tell your doctor if you have or have ever had a problem that affects your nervous system before you are given Remicade. This includes multiple sclerosis, Guillain-Barre syndrome, if you have fits or have been diagnosed with 'optic neuritis'.

Tell your doctor straight away if you get symptoms of a nerve disease during treatment with Remicade. Signs include changes in your vision, weakness in your arms or legs, numbness or tingling in any part of your body.

Abnormal skin openings
- Tell your doctor if you have any abnormal skin openings (fistulae) before you are given Remicade.

Vaccinations
- Talk to your doctor if you recently have had or are due to have a vaccine
- You should not receive certain vaccines while using Remicade.

Operations or dental procedures
- Tell your doctor if you are going to have any operations or dental procedures
- Tell your surgeon or dentist performing the procedure that you are having treatment with Remicade by showing them your Patient Alert Card.

If you are not sure if any of the above apply to you, talk to your doctor before you are given Remicade.

Using other medicines
Patients who have inflammatory diseases already take medicines to treat their problem. These medicines may cause side effects. Your doctor will advise you what other medicines you must keep using while you are having Remicade.

Tell your doctor if you are using or have recently used any other medicines, including medicines obtained without a prescription, such as vitamins and herbal medicines.

In particular, tell your doctor if you are using any of the following medicines:
- Medicines that affect your immune system
- Kineret (anakinra). Remicade and Kineret should not be used together
- Orencia (abatacept). Remicade and Orencia should not be used together.

If you are not sure if any of the above apply to you, talk to your doctor or pharmacist before using Remicade.

Pregnancy and breast-feeding
- Talk to your doctor before you are given Remicade if you are pregnant or might become pregnant
- Remicade is not recommended for use during pregnancy
- You must avoid getting pregnant when you are being treated with Remicade and for 6 months after you stop being treated with it. Make sure you use contraception during this time
- Do not breast-feed when you are being treated with Remicade or for 6 months after your last treatment with Remicade.

Driving and using machines
Remicade is not likely to affect your ability to drive or use tools or machines. If you feel tired or unwell after having Remicade, do not drive or use any tools or machines.

3. HOW REMICADE WILL BE GIVEN

How Remicade is given

- Remicade will be given to you by your doctor or nurse, in a hospital or clinic
- Your doctor or nurse will prepare the Remicade solution for injection
- The Remicade solution will be slowly injected (over a 2 hour period) into one of your veins. This will usually be in your arm. This is called an 'intravenous infusion' or drip
- You will be monitored while you are given Remicade and also for 1 to 2 hours after.

How much Remicade is given

- The doctor will decide your dose (in mg) and how often you will be given Remicade. This will depend on your disease, weight and how well you respond to Remicade
- The table below shows how often you will usually have this medicine.

1st treatment	0 weeks
2nd treatment	2 weeks after your 1st treatment
3rd treatment	6 weeks after your 1st treatment
Further treatments	Every 6 to 8 weeks depending on your disease

Rheumatoid arthritis
The usual dose is 3 mg for every kg of body weight. After the third treatment, your doctor may decide to give you Remicade over a 1 hour period.

Psoriatic arthritis, ankylosing spondylitis (Bechterew's disease), psoriasis, ulcerative colitis and Crohn's disease
The usual dose is 5 mg for every kg of body weight.

Use in children
Remicade should only be used in children if they are being treated for Crohn's disease. These children must be 6 years of age or older.

If you are given too much Remicade
As this medicine is being given by your doctor or nurse, it is unlikely that you will be given too much. There are no known side effects of having too much of Remicade.

If you forget or miss your Remicade infusion
If you forget or miss an appointment to receive Remicade, make another appointment as soon as possible.

If you have any further questions on the use of this medicine, ask your doctor.

4. POSSIBLE SIDE EFFECTS

Like all medicines, Remicade can cause side effects, although not everybody gets them. Most side effects are mild to moderate. However some patients may experience serious side effects and may require treatment. Side effects may also occur after your treatment with Remicade has stopped.

Tell your doctor straight away if you notice any of the following:

- **Signs of an allergic reaction** such as swelling of your face, lips, mouth or throat which may cause difficulty in swallowing or breathing, skin rash, hives, swelling of the hands, feet or ankles. An allergic reaction could happen within 2 hours of your injection or later. More signs of an allergic reaction that may happen up to 12 days after your injection include pain in the muscles, fever, joint or jaw pain, sore throat or headache

- **Signs of a heart problem** such as shortness of breath, swelling of your feet or changes in your heart beat
- **Signs of infection (including TB)** such as fever, feeling tired, (persistent) cough, shortness of breath, flu-like symptoms, weight loss, night sweats, diarrhoea, wounds, dental problems or burning when urinating
- **Signs of a lung problem** such as coughing, breathing difficulties or tightness in the chest
- **Signs of a nervous system problem (including eye problems)** such as fits, tingling or numbness in any part of your body, weakness in arms or legs, changes in eyesight such as double vision or other eye problems
- **Signs of a liver problem** such as yellowing of the skin or eyes, dark-brown coloured urine or pain in the upper right side of the stomach area, fever
- **Signs of an immune system disorder called lupus** such as joint pain or a rash on cheeks or arms that is sensitive to the sun
- **Signs of a low blood count** such as persistent fever, bleeding or bruising more easily or looking pale.

Tell your doctor straight away if you notice any of the above.

Common side effects (affects 1 to 10 patients in 100)
- Increase in liver enzymes (shown in blood tests taken by your doctor)
- Allergic reactions to foreign proteins
- Lung or chest infections such as bronchitis or pneumonia
- Difficult or painful breathing, chest pain
- Stomach pain, diarrhoea, feeling sick or indigestion
- Nettle-type rash (hives), itchy rash or dry skin
- Viral infections such as herpes or flu
- Balance problems or feeling dizzy
- Fever, increased sweating
- Warm, red skin (flushing)
- Feeling tired or weak
- Sinus infections
- Headache.

Uncommon side effects (affects 1 to 10 patients in 1,000)
- Circulation problems such as low or high blood pressure, shortage of blood supply, swelling or narrowing of a vein, bruising, hot flush or nosebleed
- Skin problems such as blistering, boils, eczema, warts, abnormal skin colouration or pigmentation, hair loss or swollen lips
- Severe allergic reactions (e.g. anaphylaxis), an immune system disorder called lupus
- Reactions where you have had the injection such as pain, swelling, redness or itching
- Wounds taking longer to heal, chills, or a build up of fluid under the skin causing swelling
- Swelling of the gall bladder or changes in how your liver works (shown in blood tests)
- Depression, feeling forgetful, irritable, confused, nervous, lack of interest or emotion
- Eye problems including blurred or reduced vision, red or puffy eyes or sties
- Problems sleeping
- Bacterial infections such as blood poisoning, abscess or infection under the skin (cellulitis)
- Heart problems (such as worsening of existing heart failure)
- Nervous system disorders (multiple sclerosis-like disease)
- Stomach pain or cramps, heartburn, constipation
- Fungal infections such as yeast infection
- Lung problems (such as oedema)
- Pain in the joints, muscles or back
- Kidney or urinary tract infections
- Blood problems such as anaemia

- Infections of the vagina.

Rare side effects (affects 1 to 10 patients in 10,000)
- A type of blood cancer (lymphoma)
- Bleeding or a hole around the stomach area or blockage of the intestine
- Your blood not supplying enough oxygen to your body
- Inflammation of the lining of the brain (meningitis)
- Too much fluid around the lungs (pleural effusion)
- Abnormal tissue swelling or growth
- Swelling of the liver (hepatitis)
- Fast heart beat (tachycardia).

Other side effects (the frequency is unknown)
- Cancer in children and adults
- A rare blood cancer affecting young people (hepatosplenic T-cell lymphoma)
- Psoriasis,serious skin problems such as toxic epidermal necrolysis, Stevens-Johnson Syndrome or erythema multiforme
- Serious nervous system disorders such as Guillain-Barré syndrome, optic neuritis or transverse myelitis
- A build up of fluid in the lining of the heart (pericardial effusion)
- Hepatitis B infection when you have had hepatitis B in the past
- Serious lung problems (such as interstitial lung disease)
- Infections due to a weakened immune system
- Swelling of small blood vessels (vasculitis)
- Swelling of your pancreas (pancreatitis)
- Infection with Salmonella bacteria
- Liver failure or liver damage.

If any of the side effects gets serious or if you notice any side effects that are not listed in this leaflet, please tell your doctor.

5. HOW TO STORE REMICADE

Remicade will be stored by the health professionals at the hospital or clinic. The storage details should you need them are as follows:
- Keep out of the reach and sight of children
- Do not use Remicade after the expiry date which is stated on the label and the carton. The expiry date refers to the last day of that month
- Store in a refrigerator (2°C to 8°C).
- It is recommended that when Remicade is prepared for infusion, it is used as soon as possible (within 3 hours). However, if the solution is prepared in germ-free conditions, it can be stored in a refrigerator at 2°C to 8°C for 24 hours

The solution should not be used if discoloured or if there are particles present.

6. FURTHER INFORMATION

What Remicade contains
- The active substance is infliximab. Each vial contains 100 mg of infliximab. After preparation each ml contains 10 mg of infliximab
- The other ingredients are sucrose, polysorbate 80, monobasic sodium phosphate and dibasic sodium phosphate.

What Remicade looks like and contents of the pack
Remicade is supplied as a glass vial containing a powder for concentrate for solution for infusion. The powder is a freeze-dried white pellet.
Remicade is produced in packs of 1, 2, 3, 4 or 5 vials. Not all pack sizes may be marketed.

Marketing Authorisation Holder
Centocor B.V.
Einsteinweg 101
2333 CB Leiden
The Netherlands

Manufacturer
Centocor B.V.
Einsteinweg 101
2333 CB Leiden
The Netherlands

For any information about this medicine, please contact the local representative of the Marketing Authorisation Holder:

België/Belgique/Belgien
Rue de Stalle/Stallestraat 73
B-1180 Bruxelles/Brussel/Brüssel
Tél/Tel: + 32-(0)2 370 92 11

България
Ийст Парк Трейд Център
Бул. „Н.Й.Вапцаров" 53А, ет. 2
BG-София 1407
Тел.: +359 2 806 3030

Česká republika
Ke Štvanici 3
CZ-186 00 Praha 8
Tel: +420 221771250

Danmark
Lautrupbjerg 2
DK-2750 Ballerup
Tlf: + 45-44 39 50 00

Deutschland
Thomas-Dehler-Straße 27
D-81737 München
Tel: + 49-(0)89 627 31-0

Eesti
Järvevana tee 9
EE-11314 Tallinn
Tel: + 372 654 96 86

Luxembourg/Luxemburg
Rue de Stalle 73
B-1180 Bruxelles/Brüssel
Belgique/Belgien
Tél/Tel: + 32-(0)2 370 92 11

Magyarország
Alkotás u. 53
H-1123 Budapest
Tel.:+36 1 457-8500

Malta
Triq l-Esportaturi
Mriehel
Birkirkara BKR 3000
Tel.: +35622778000

Nederland
Walmolen 1
NL-3994 DL Houten
Tel: + 31-(0)800 999 90 00

Norge
Pb. 398
N-1326 Lysaker
Tlf: + 47 67 16 64 50

Österreich
Am Euro Platz 2
A-1120 Wien
Tel: + 43 (0) 1 813 12 31

Ελλάδα
Αγίου Δημητρίου 63
GR-174 55 Άλιμος
Τηλ: + 30-210 98 97 300

España
Km. 36, Ctra. Nacional I
E-28750 San Agustín de Guadalix - Madrid
Tel: + 34-91 848 85 00

France
34 avenue Léonard de Vinci
F-92400 Courbevoie
Tél: + 33-(0)1 80 46 40 40

Ireland
Shire Park
Welwyn Garden City
Hertfordshire AL7 1TW
United Kingdom
Tel: + 44-(0)1 707 363 636

Ísland
Hõrgatún 2
IS-210 Garðabær
Sími: + 354 535 70 00

Italia
Via fratelli Cervi snc
Centro Direzionale Milano Due
Palazzo Borromini
I-20090 Segrate (Milano)
Tel: + 39-02 21018.1

Κύπρος
Οδός Αγίου Νικολάου, 8
CY-1055 Λευκωσία
Τηλ: +357-22 757188

Latvija
Bauskas 58a -401
Rīga LV-1004
Tel: + 371-7 21 38 25

Lietuva
Kęstučio g. 65/40
LT 08124 Vilnius
Tel. + 370 52 101 868

Polska
ul. Taśmowa 7
PL-02-677 Warszawa
Tel.: + 48-(0)22 478 41 50

Portugal
Rua Agualva dos Açores 16
P-2735-557 Agualva-Cacém
Tel: + 351-21 433 93 00

România
Şos. Bucureşti-Ploieşti, nr. 17-21, Băneasa Center,
et. 8, sector 1
Bucureşti, 013682-RO
Tel. + 40 21 233 35 30

Slovenija
Dunajska 22
SI-1000 Ljubljana
Tel. + 386 01 3001070

Slovenská republika
Strakova 5
SK-811 01 Bratislava
Tel.: + 421 (2) 5920 2712

Suomi/Finland
PL 86/PB 86
FIN-02151 Espoo/Esbo
Puh/Tel: + 358-(0)20-7570 300

Sverige
Box 6185
S-102 33 Stockholm
Tel: + 46-(0)8 522 21 500

United Kingdom
Shire Park
Welwyn Garden City
Hertfordshire AL7 1TW
Tel: + 44-(0)1707 363 636

This leaflet was last approved in {MM/YYYY}.

Detailed information on this medicine is available on the European Medicines Agency web site: http://www.ema.europa.eu. There are also links to other websites about rare diseases and treatments.

THE FOLLOWING INFORMATION IS INTENDED FOR MEDICAL OR HEALTHCARE PROFESSIONALS ONLY:

Instructions for use and handling – reconstitution, dilution and administration

1. Calculate the dose and the number of Remicade vials needed. Each Remicade vial contains 100 mg infliximab. Calculate the total volume of reconstituted Remicade solution required.

2. Under aseptic conditions, reconstitute each Remicade vial with 10 ml of water for injections, using a syringe equipped with a 21-gauge (0.8 mm) or smaller needle. Remove flip-top from the vial and wipe the top with a 70% alcohol swab. Insert the syringe needle into the vial through the centre of the rubber stopper and direct the stream of water for injections to the glass wall of the vial. Do not use the vial if the vacuum is not present. Gently swirl the solution by rotating the vial to dissolve the lyophilised powder. Avoid prolonged or vigorous agitation. DO NOT SHAKE. Foaming of the solution on reconstitution is not unusual. Allow the reconstituted solution to stand for 5 minutes. Check that the solution is colourless to light yellow and opalescent. The solution may develop a few fine translucent particles, as infliximab is a protein. Do not use if opaque particles, discolouration, or other foreign particles are present.

3. Dilute the total volume of the reconstituted Remicade solution dose to 250 ml with sodium chloride 9 mg/ml (0.9%) solution for infusion. This can be accomplished by withdrawing a volume of the sodium chloride 9 mg/ml (0.9%) solution for infusion from the 250-ml glass bottle or infusion bag equal to the volume of reconstituted Remicade. Slowly add the total volume of reconstituted Remicade solution to the 250-ml infusion bottle or bag. Gently mix.

4. Administer the infusion solution over a period of not less than the infusion time recommended for the specific indication. Use only an infusion set with an in-line, sterile, non-pyrogenic, low protein-binding filter (pore size 1.2 micrometer or less). Since no preservative is present, it is recommended that the administration of the solution for infusion is to be started as soon as possible and within 3 hours of reconstitution and dilution. When reconstitution and dilution are performed under aseptic conditions, Remicade infusion solution can be used within 24 hours if stored at 2°C to 8°C. Do not store any unused portion of the infusion solution for reuse.

5. No physical biochemical compatibility studies have been conducted to evaluate the co-administration of Remicade with other agents. Do not infuse Remicade concomitantly in the same intravenous line with other agents.

6. Visually inspect Remicade for particulate matter or discolouration prior to administration. Do not use if visibly opaque particles, discolouration or foreign particles are observed.

7. Any unused product or waste material should be disposed of in accordance with local requirements.
